# Structural Optimization and Structure–Activity Relationship of 4-Thiazolidinone Derivatives as Novel Inhibitors of Human Dihydroorotate Dehydrogenase

**DOI:** 10.3390/molecules24152780

**Published:** 2019-07-31

**Authors:** Fanxun Zeng, Lina Quan, Guantian Yang, Tiantian Qi, Letian Zhang, Shiliang Li, Honglin Li, Lili Zhu, Xiaoyong Xu

**Affiliations:** 1 Shanghai Key Laboratory of Chemical Biology, School of Pharmacy, East China University of Science and Technology, 130 Meilong Road, Shanghai 200237, China; 2 Shanghai Key Laboratory of New Drug Design, State Key Laboratory of Bioreactor Engineering, School of Pharmacy, East China University of Science & Technology, Shanghai 200237, China

**Keywords:** *h*DHODH inhibitors, structural optimization, structure–activity relationship, 4-thiazolidinones

## Abstract

Human dihydroorotate dehydrogenase (*h*DHODH), one of the attractive targets for the development of immunosuppressive drugs, is also a potential target of anticancer drugs and anti-leukemic drugs. The development of promising *h*DHODH inhibitors is in high demand. Based on the unique binding mode of our previous reported 4-thiazolidinone derivatives, via molecular docking method, three new series 4-thiazolidinone derivatives were designed and synthesized as *h*DHODH inhibitors. The preliminary structure–activity relationship was investigated. Compound **9** of biphenyl series and compound **37** of amide series displayed IC_50_ values of 1.32 μM and 1.45 μM, respectively. This research will provide valuable reference for the research of new structures of *h*DHODH inhibitors.

## 1. Introduction

Pyrimidine, as a key precursor of RNA, DNA, glycoproteins, and phospholipids, plays a critical role in cellular metabolism and cell growth [1,2]. Human dihydroorotate dehydrogenase (*h*DHODH) is a mitochondrial enzyme that catalyzes the fourth step of *de novo* pyrimidine biosynthesis [3,4]. Unlike resting or fully differentiated cells which acquire pyrimidine mainly by the salvage pathways, rapidly proliferating cells, such as activated T cells, depend heavily on *de novo* nucleotide synthesis to meet their increased demand for nucleic acid precursors [5,6]. Thus, *h*DHODH would be an ideal drug target for the treatment of variety of diseases such as tumor, immunological disorders and acute myeloid leukemia [7,8,9,10,11,12,13,14].

**Figure 1:**
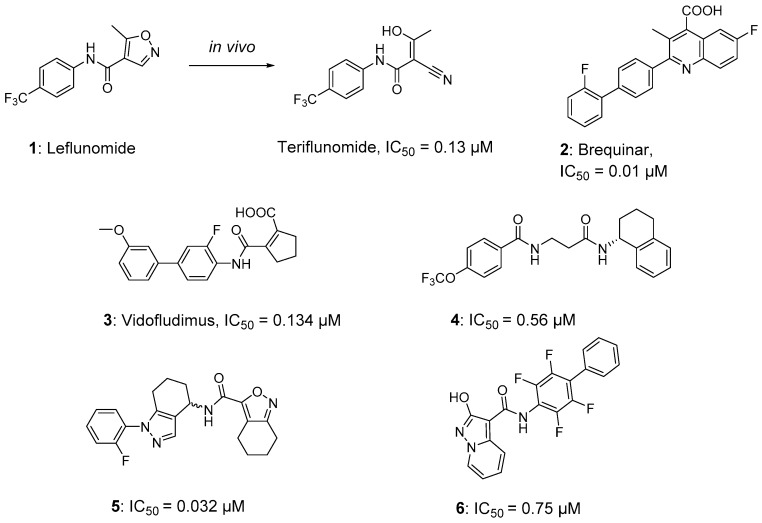
Structures of representative inhibitors of *h*DHODH.

Many *h*DHODH inhibitors have been developed and exhibited excellent therapeutic efficacy [15]. For example, leflunomide (1, Figure 1) was approved by FDA for the treatment of rheumatoid arthritis in 1998 [16,17]. Then this compound was found to be a prodrug, forming its active metabolite teriflunomide via a base caused ring-opening reaction in vivo [18,19]. Teriflunomide was developed to cure multiple sclerosis later [20]. Brequinar (2), one of the strongest inhibitors of *h*DHODH, only showed modest anticancer effects on a number of solid tumors in a phase II clinical trial [21,22]. Its effects on autoimmune diseases and viral diseases were also extensively studied [23,24,25]. Vidofludimus (3) was proven to be a promising pharmaceutical agent in systemic lupus erythematosus, inflammatory bowel disease, rheumatoid arthritis, and transplantation [7,26,27,28]. Vidofludimus also restored myeloid differentiation in leukemia cell lines at concentrations that are one log digit lower than those achieved in experiments with brequinar [14]. The development of ML390 (4) that induced differentiation in acute myeloid leukemia (AML) demonstrated that the inhibition of *h*DHODH could overcome differentiation blockade in AML [29]. Recently compound 5 was reported to reduce tumor growth by increasing p53 synthesis [30]. Compound 6 showed brequinar-like *h*DHODH potency in vitro and was superior in terms of cytotoxicity and immunosuppression. In general, it is significant to develop promising *h*DHODH inhibitors for the severe side effects of leflunomide/teriflunomide and the lack of newly marketed *h*DHODH inhibitors.

**Figure 2:**
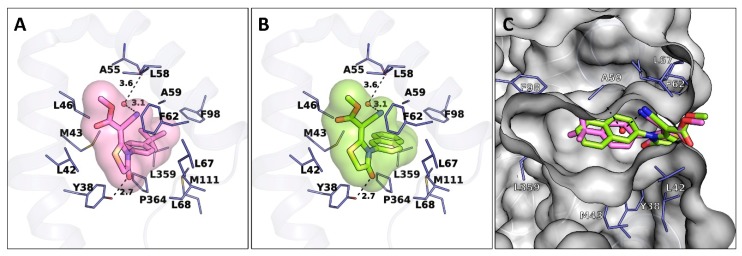
Proposed binding modes of 4-thiazolidinone derivatives **7** (**A**) and **8** (**B**). The X-ray crystal structure of *h*DHODH with PDB ID of 4LS1 was used in molecular docking. Figure 2C is an overlay of compounds **7** and **8**.

Recently we had described 4-thiazolidinone derivatives **7** and **8** as novel *h*DHODH inhibitors [31]. It was found that the binding mode of 4-thiazolidinone with *h*DHODH was different from the classic inhibitors, that is, the thiazolidinone fragment locates at the outside of the bind pocket, cyano group contacts ALA-59 by a water-mediated hydrogen bond, carbonyl and TYR-38 forms hydrogen bond interaction, while the aromatic fragment contacts MET-43 by hydrophobic interaction (Figure 2A). But, it was found that compound **8** only occupies the hydrophobic subsite of the ubiquinone-binding site with its naphthalenyl group (Figure 2B,C) [31]. The binding mode of compound **8** suggested us that the replacement of its naphthalenyl group with a suitable longer aryl group could orient the new ligand towards the inner side of the binding site. An additional polar group, such as carboxyl and acetyl, would generate hydrogen bond or salt bridge interactions with residue Arg-136 in the hydrophilic subsite of the ubiquinone-binding site and may improve compounds’ binding affinity against *h*DHODH. Therefore, further structural optimization of 4-thiazolidinone derivatives that focused on the decoration of the aryl group to identify more potent *h*DHODH inhibitors was presented in this study.

## 2. Results and Discussion

### 2.1. Molecular Design Strategies

**Figure 3:**
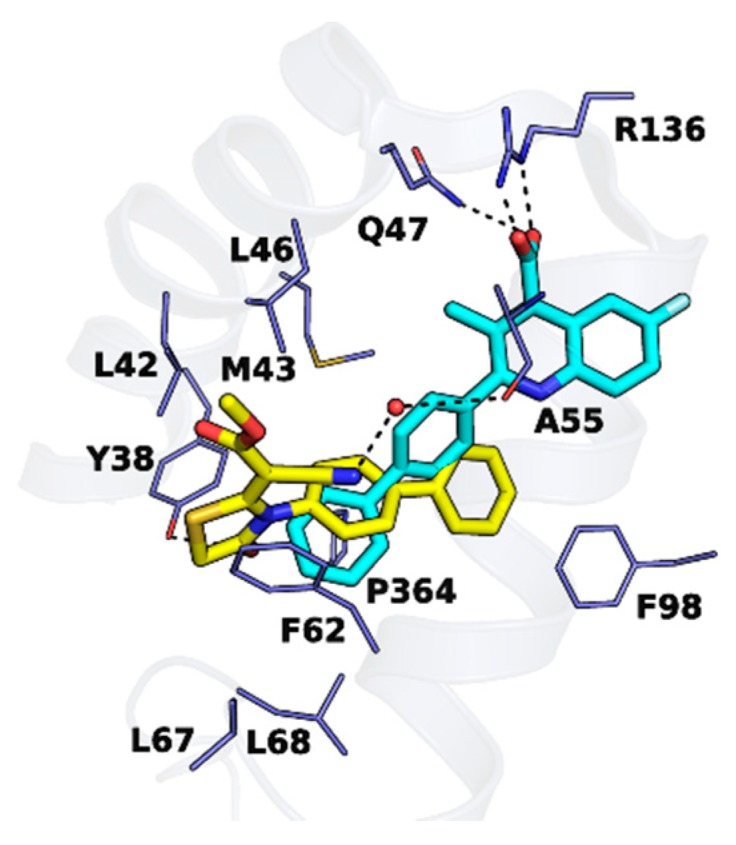
Binding mode of 4-thiazolidinone derivative **9**. Compound **9** is displayed as yellow sticks and brequinar is shown as cyan sticks.

**Figure 4:**
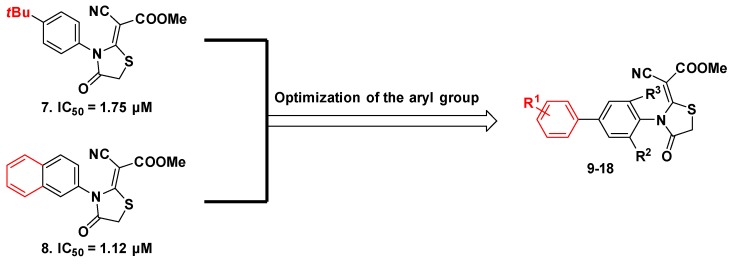
Molecular design strategies of 4-thiazolidinones with biphenyl as *h*DHODH inhibitors.

**Figure 5:**
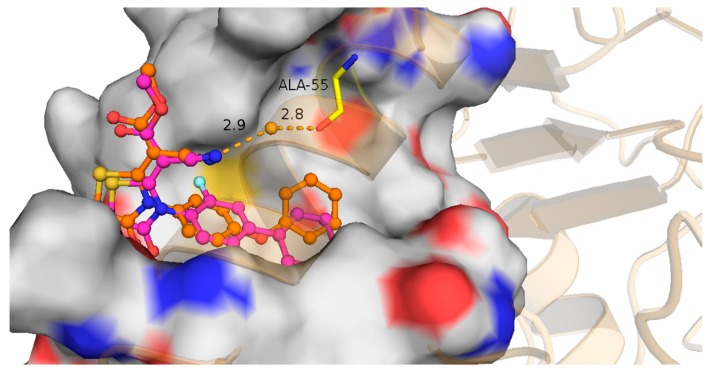
Binding modes of 4-thiazolidinones with biphenyl ether structure (magenta sticks: compound **10**, orange sticks: compound **19**).

**Figure 6:**
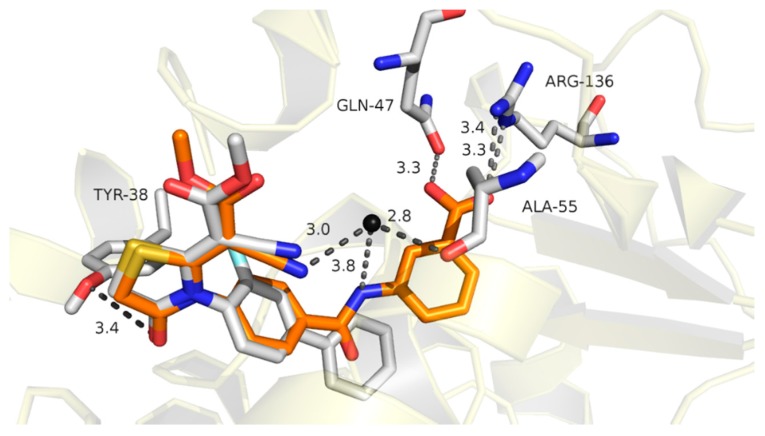
Proposed binding mode of benzamide-substituted 4-thiazolidinones as *h*DHODH inhibitors (orange sticks: compound **41**, gray sticks: compound **10**).

**Figure 7:**
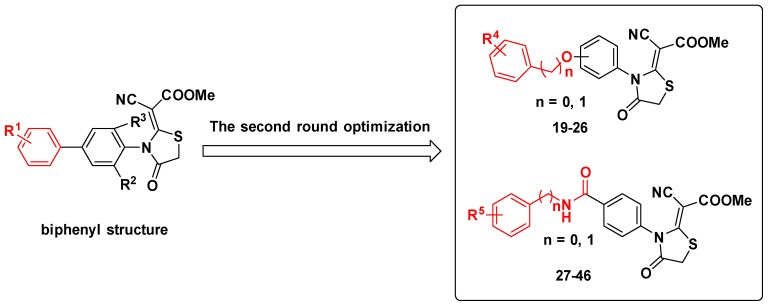
Design and modification strategies of 4-thiazolidinones with biphenyl ether and amide as *h*DHODH inhibitors.

In order to probe the inner subsite of the ubiquinone-binding site of *h*DHODH, biphenyl derivatives were firstly designed and synthesized. The binding mode of **9** was simulated using the same molecular docking method reported previously [31,32]. It was shown that the introduction of 4-phenyl moiety could enhance the hydrophobic interaction between the biphenyl compounds and the binding pocket (Figure 3 and Figure 4). In addition, by observing the binding modes of **9**, we found that there is some space for substitution at the *ortho*-position of the phenyl group to enhance the hydrophobic interactions with residues Met43, Leu46, Met111 and Pro364. Therefore, we tried to introduce a small fluorine atom into the structure and synthesized compounds **10-18**. However, compared with brequinar, a *para*-phenyl might collide with the binding pocket, which would influence the binding affinity against the target *h*DHODH (Figure 3). Therefore, phenoxy (phenoxymethyl) and phenyl amide groups were introduced to improve the binding strength by adjusting the molecular configuration (Figure 5). After molecular docking simulations, it was found that the introduction of an ether bond could cause an angle (about 109°) to adjust the orientation of the phenyl group, which may be helpful to avoid the steric hindrance between the ligand and the receptor (Figure 5). If the linker was replaced by a longer amide bond, the whole molecule would also become longer, thus enabling the phenyl group to contact residues in the inner binding site. When substituting a carboxyl group to the meta-position of the phenyl group of the benzamide derivatives next, the carboxyl group would form hydrogen bond interactions with residues Gln47 and Arg136, which are beneficial for strengthening the binding affinity of the compounds (Figure 6). In addition, the amide bond could participate in a water-mediated hydrogen bond network, which may be favorable for molecular binding. The results of docking simulations suggested us that the design strategies may be feasible to improve the potency of the compounds. The detailed modification strategies are described in Figure 4 and Figure 7.

### 2.2. Chemistry

**Scheme 1:**
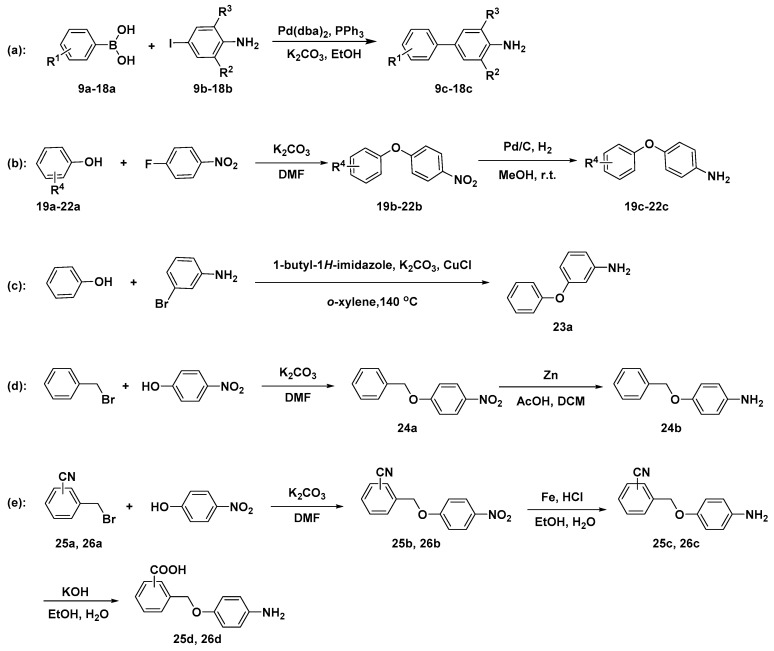
Synthesis of key intermediates of compounds **9**–**26**.

Key intermediates of compounds **9**–**26** were synthesized according to Scheme 1. Compounds **9c**–**18c** were obtained by Suzuki cross-coupling reactions of compounds **9a**–**18a** with **9b**–**18b**. Etherification of compounds **19a**–**22a** with 1-fluoro-4-nitrobenzene yielded compounds **19b**–**22b**, which were reduced to afford compounds **19c**–**22c**. Phenol was coupled with 1-fluoro-4-nitrobenzene to give compound **23a**. Etherification of (bromomethyl) benzene with 4-nitrophenol afforded compound **24a**, which was reduced to give compound **24b**. Compounds **25b** and **26b** were obtained by etherification of compounds **25a, 26a** with 4-nitrophenol, which were further reduced and hydrolyzed to give compounds **25d** and **26d**.

**Scheme 2:**
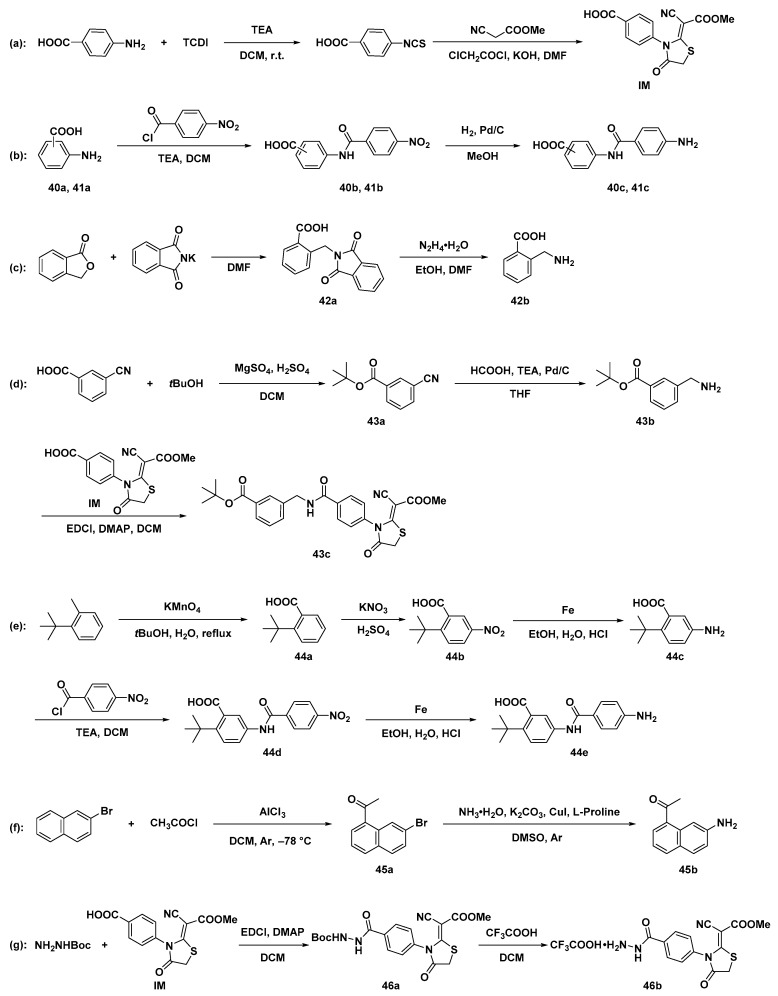
Synthesis of **IM** and key intermediates of compounds **40**–**46**.

Compound **IM** and key intermediates of compounds **40**–**46** were obtained according to Scheme 2. 4-isothiocyanatobenzoic acid was prepared by reaction of 4-aminobenzoic acid with 1,1’-thiocarbonyldiimidazole (TCDI) in the presence of TEA. 4-Isothiocyanatobenzoic acid was treated with methyl 2-cyanoacetate and potassium hydroxide in DMF to provide ketene-N, S-acetal salt, which then reacted with 2-chloroacetyl chloride to give key intermediate **IM**.

Compounds **40c**, **41c** were obtained by reduction of compounds **40b**, **41b**, which were prepared by amidation reactions of compounds **40a**, **41a** with 4-nitrobenzoyl chloride. Compound **42b** was synthesized by hydrazinolysis of compound **42a**, which was prepared by reaction of phthalide with potassium phthalimide. 

The important intermediate **43c** was generated from starting material 3-cyanobenzoic acid via protection, reduction, and amidation. Compound **44e** was prepared from 1-(*tert*-butyl)-2-methylbenzene via oxidation, nitration, reduction, amidation, and reduction. 

Compound **45b** was synthesized by amination of compound **45a**, which was obtained by Friedel -Crafts acylation of 2-bromonaphthalene. Intermediate **46b** was obtained by removing Boc group of compound **46a**, which was obtained by amination of **IM**.

**Scheme 3:**
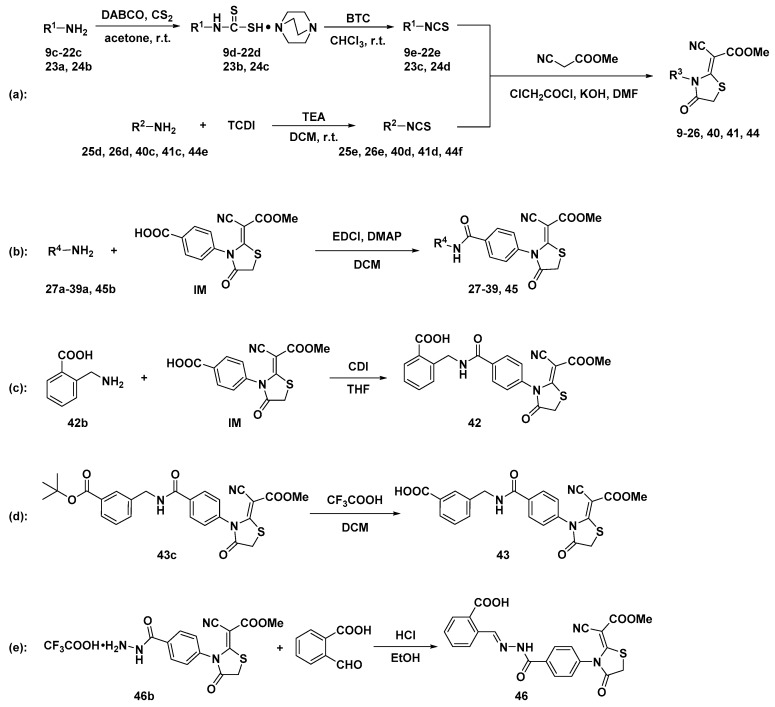
Synthesis of compounds **9**–**46**.

The synthetic route to obtain target compounds **9**–**46** was shown in Scheme 3. Isothiocyanates **9e**–**22e**, **23c**, and **24d** were obtained from corresponding aryl amines by two-step reactions. Key intermediates **25e**, **26e**, **40d**, **41d**, **44f** and compounds **9**–**26**, **40**, **41**, **44** were prepared by the same route of 4-isothiocyanatobenzoic acid and **IM**, respectively. Compounds **27**–**39**, **42**, and **45** were generated by amination of intermediates **27a**–**39a**, **42b**, **45b** with **IM**. Compound **43** was afforded by deprotection of compound **43**. The imidization of **46b** with 2-formylbenzoic acid was introduced to give compound **46**.

### 2.3. Inhibitory Activities against hDHODH and SAR Study

In order to identify more potent compounds, biphenyl group was introduced for the size and hydrophobicity of the aryl group which is essential for the activities. As shown in Table 1, the activity of unsubstituted biphenyl derivative **9** was equal to that of compounds **7** and **8**. Introducing a fluorine atom into the biphenyl group (compounds **10** and **11**) decreased the inhibitory activities. 2′-Methyl analog **12** had slightly less activity than **10**. Introduction of a methoxy group into the 2′-, 3′-, or 4′-positions of the biphenyl group (compounds **13**–**15**) resulted in roughly 2-fold reduction in inhibitory activity compared to compound **10**, amongst the three compounds, the 3′-methoxy derivative **14** was slightly more potent than compounds **13**, **15**. Introduction of electron-withdrawing groups (compounds **16**–**18**) also failed to enhance the potency. Moreover, the results of the bioassay also showed that the *ortho*-substituted fluorine atom (compounds **10** and **11**) did not make a positive contribution to the binding affinity, indicating that there may be steric clashes.

Since the biphenyl derivatives only showed moderate potency, biphenyl groups were replaced further by flexible aryl ethers further (Table 2). To our disappointment, diphenyl ether derivatives **19**–**23** displayed markedly diminished activity. The 3-phenoxyphenyl analog **23** was also inactive. Benzyl ether derivative **24** exhibited moderate activity with an IC_50_ value of 4.32 μM. The activity decreased when a carboxyl group (compounds **25** and **26**) was introduced.

Then the moderate rigid and longer amide structure was introduced as a linker (compounds **27**–**46**, Table 2). Phenyl derivative **28** showed a moderate activity, while the cyclohexyl analog **27** gave a dramatically decreased activity. It indicated that rigid aromatic ring is better than flexible cyclohexyl ring. Next we tried to introduce substituent groups into the phenyl group. A 4-*tert*-butyl substituent (compound **37**) was tolerated, while the introduction of smaller substituent groups such as CH_3_, F, Cl, I, CF_3_, OCH_3_, CN etc. into the 2, 3 or 4-positions (compounds **29**–**36**) was detrimental to the activity. The naphthalen-2-yl derivative **38** and benzyl derivative **39** were inactive. Subsequently, COOH and carbonyl groups were introduced to generate hydrogen bonds or salt bridge interactions with residue Arg136 in the hydrophilic subsite of the ubiquinone-binding site. However, the inhibition rates of the corresponding analogs **40**–**46** at 10 μM were still less than 50%. The bioassay results did not match our modeling results. It indicated that neither ether nor amide is an appropriate linker to extend the molecules to fit well in the pocket and eventually contact the inner residues Arg136 and Gln47.

Although the introduction of a biphenyl, diphenyl ether and amide structures into 4- thiazolidinone did not obviously increase the rate of inhibition of *h*DHODH, the attempts can provide some ideas for *h*DHODH inhibitor lead identification. We speculate that there may exist steric clashes caused by inappropriate biphenyl, ether and amide group linkers. Thus, we will try some other linkers like -C=N-N-, -C-C(=O)-, and -C-C(=O)-C- to replace the O of biphenyl ether or the CONH of the amide series in our future work to further investigate the SAR and to obtain more potent DHODH inhibitors. In addition, it was found that any changes to the structure connecting the N-terminus of thiazolidone have a weak influence on the bioactivities, which means that thiazolidone is important to increase the activity. In our future work, we will try to select other heterocycles and investigate the stability of thiazolidones under the determination conditions to further establish the factors influencing the bioactivity.

## 3. Materials and Methods

### 3.1. General Information

Unless otherwise indicated, all commercially available solvents and reagents were purchased directly from commercial suppliers and used as received without further purification. Melting points (m.p.) were recorded on Büchi B540 apparatus (Büchi Labortechnik AG, Flawil, Switzerland) and are uncorrected. ^1^H-NMR, ^19^F-NMR and ^13^C-NMR spectra were recorded on an AM-400 (^1^H at 400 MHz, ^13^C at 100 MHz, ^19^F at 376 MHz) spectrometer (Bruker BioSpin AG, Fällanden, Switzerland) with CDCl_3_ or DMSO-*d*_6_ as the solvent and TMS as the internal standard. Chemical shifts are reported in δ (parts per million) values. The following abbreviations were used to explain the multiplicities: s = singlet, d = doublet, t = triplet, q = quartet, m = multiplet, coupling constant (Hz) and integration. High-resolution electron mass spectra (ESI-TOF) were performed on a Micromass LC-TOF spectrometer (Waters Co.,Ltd., Milford, MA, USA). High resolution mass spectra (HRMS) were recorded under electron impact (70 eV) conditions using a MicroMass GCT CA 055 instrument (Waters Co.,Ltd., Milford, MA, USA). Analytical thin-layer chromatography (TLC) was carried out on precoated plates (silica gel 60 F254) and spots were visualized with ultraviolet (UV) light.

### 3.2. Chemistry

#### 3.2.1. Synthesis of Key Intermediates of Compounds **9**–**26**.

##### General Procedure for the Synthesis of Intermediates **9c**–**18c**

Substituted 4-iodoanilines **9b**–**18b** (5 mmol), substituted phenylboronic acids **9a**–**18a** (6 mmol), an orange solution of Pd(dba)_2_ (2 mol%) and triphenylphosphine (6 mol%) were added in a tube. The tube was evacuated and back-filled with argon. Potassium carbonate (20 mmol) solution (10 mL, 2 mol/L) and ethanol (10 mL) were added using syringe, and the mixture was stirred and refluxed for 3–5 h. After completion of the reaction, the reaction solution was extracted three times with ethyl acetate. The combined organic phases were dried over Na_2_SO_4_, and the solvent was removed under reduced pressure. The residue was purified by column chromatography (Petroleum ether (PE)/Ethyl acetate (EA)=10:1, V/V), to give **9c**–**18c** in 65%−95% yield. (Spectrums for target compounds **7**–**46** could be accessed in Supplementary Materials).

##### General Procedure for the Synthesis of Intermediates **19b**–**22b**

K_2_CO_3_ (50 mmol) was carefully added to a solution of **19a**–**22a** (12 mmol) and 1-fluoro-4-nitrobenzene (10 mmol) in DMF (8 mL). The mixture was stirred at heating condition (120 °C for **19b**, **20b**, and **22b**, 60 °C for **21b**) until the reaction was complete. The reaction mixture was diluted with water (20 mL), extracted with EA after cooling to room temperature. The combined organic layer was washed with 1 M NaOH, 1 M HCl and saturated salt water in order. Then the mixture was dried (Na_2_SO_4_), concentrated under reduced pressure to give **19b**–**22b** in yield of 70–90%, which was used in the next step without further purification.

##### General Procedure for the Synthesis of Intermediates **19c**–**22c**

Compounds **19b**–**22b** (8 mmol) and Pd/C (10% w/w) were added to MeOH (40 mL). The resulted mixture was reacted under H_2_ atmosphere at room temperature for 2 h. The mixture was filtered and the filtrate was concentrated under reduced pressure to give **19c**–**22c** in yield of 95–98%, which was used in the next step without further purification.

##### Synthesis of Intermediate **23a**

Phenol (12 mmol), 3-bromoaniline (10 mmol), K_2_CO_3_ (20 mmol), 1-butyl-1*H*-imidazole (5 mmol), and CuCl (4.5 mmol) were added to o-xylene (10 mL) under Ar atmosphere, and the mixture was heated to 140 °C for 20h. After cooling to room temperature, the mixture was filtered. The filtrate was concentrated under reduced pressure and purified by column chromatography (PE/EA=4:1, V/V) to give **23a** in yield of 90%.

##### Synthesis of Intermediate **24a**

K_2_CO_3_ (20 mmol) was added to a solution of (bromomethyl)benzene (12 mmol) and 4-nitrophenol (10 mmol) in DMF (10 mL). The resulted mixture was allowed to react at 90 °C for 2 h. The mixture was diluted with water (50 mL) after cooling to room temperature. The resulted mixture was filtered, washed with water. The filter cake was further crystallized from EtOH to give target compound in yield of 80%.

##### Synthesis of Intermediate **24b**

Zinc powder (120 mmol) and AcOH (400 mmol) were added to a solution of **24a** (8 mmol) in DCM (45 mL) in ice bath. The mixture was stirred at room temperature until the reaction was completed. The reaction mixture was filtered. The filtrate was washed with water, dried (Na_2_SO_4_) and concentrated under reduced pressure. The residue was further purified by column chromatography (PE/EA=4:1, V/V) to give **24b** in yield of 80%. ^1^H-NMR (CDCl_3_): δ 7.39 – 7.31 (m, 4H) 7.27 (d, *J* = 6.4 Hz, 1H), 6.69 (d, *J* = 8.4 Hz, 2H), 6.55 (d, *J* = 8.4 Hz, 2H), 4.27 (s, 2H), 4.16 (s, 2H) ppm; ^13^C-NMR (CDCl_3_): δ 147.79, 142.42, 139.61, 128.62, 127.60, 127.22, 116.21, 114.37, 49.36 ppm.

##### General Procedure for the Synthesis of Intermediates **25b** and **26b**

K_2_CO_3_ (15 mmol) was added to a solution of compounds **25a**, **26b** (10 mmol), and 4-nitrophenol (11 mmol) in DMF (30 mL) under Ar atmosphere, and the mixture was heated to 120 °C for 4 h. The mixture was diluted with water (50 mL) after cooling to room temperature. The resulted mixture was filtered, washed with water. The filter cake was dried to give target compounds **25b** and **26b**.

*2-((4-Nitrophenoxy)methyl)benzonitrile* (**25b**): ^1^H-NMR (CDCl_3_): δ 8.26–8.21 (m, 2H), 7.75 (d, *J* = 7.6 Hz, 1H), 7.70–7.64 (m, 2H), 7.52–7.47 (m, 1H), 7.11–7.06 (m, 2H). 5.35 (s, 2H) ppm; ^13^C-NMR (CDCl_3_): δ 162.97, 142.20, 138.97, 133.27, 133.15, 129.05, 128.63, 126.04, 116.85, 114.91, 111.50, 68.16 ppm.

##### General Procedure for Synthesis of Intermediates **25c** and **26c**

Reduced iron powder (100 mmol) and concentrated hydrochloric acid (0.5 mL) were carefully added to a mixture of compounds **25b**, **26b**, EtOH (60 mL) and water (6 mL). The reaction was reacted under reflux condition until the reaction was completed. The reaction mixture was filtered and the filter cake was washed with some EA. The filtrate was concentrated and used in the next step without further purification.

*3-((4-Aminophenoxy)methyl)benzonitrile* (**26c**): ^1^H-NMR (CDCl_3_): δ 7.68 (d, *J* = 7.6 Hz, 2H), 7.61 (t, *J* = 7.6 Hz, 1H), 7.40 (t, *J* = 7.6 Hz, 1H), 6.84 (d, *J* = 8.8 Hz, 2H), 6.65 (d, *J* = 8.8 Hz, 2H), 5.18 (s, 2H) ppm; ^13^C- NMR (CDCl_3_): δ 151.30, 141.22, 140.89, 133.01, 132.80, 128.45, 128.21, 117.15, 116.37, 116.35, 111.04, 68.52 ppm.

##### General Procedure for Synthesis of Intermediates **25d** and **26d**

15% KOH (100 mL) was added to a mixture of compounds **25c**, **26c** and EtOH (25 mL) under Ar atmosphere, and the mixture was reacted under reflux condition for 36 h. The reaction mixture was washed with EA (30 mL), acidified with 1 N HCl, and extracted with EA. The combined organic layer was washed with saturated NaCl, dried (Na_2_SO_4_), concentrated and purified by column chromatography (DCM/MeOH=10:1, V/V) to give **25d**, **26d** in yield of about 40%.

*2-((4-Aminophenoxy)methyl)benzoic acid* (**25d**): ^1^H-NMR (DMSO-*d*_6_): δ 10.62 (s, 3H), 7.94 (d, *J* = 7.6 Hz, 1H), 7.66–7.56 (m, 2H), 7.48–7.43 (m, 1H), 7.33 (d, *J* = 8.8 Hz, 2H), 7.08 (d, *J* = 8.8 Hz, 2H), 5.47 (s, 2H) ppm; ^13^C-NMR (DMSO-*d*_6_): δ 168.05, 157.67, 137.86, 132.09, 130.47, 129.53, 127.96, 127.77, 124.64, 124.38, 115.60, 67.91 ppm.

##### Synthesis of Intermediate **IM**

4-Aminobenzoic acid (5 mmol) was slowly added to a solution of TCDI (6 mmol) and TEA (5.5 mmol) in DCM (7.5 mL) at 0 °C. The mixture was stirred for 2h at 0 °C and then added dropwise to 4M aqueous HCl (9 mL). The precipitation was filtered and washed with 1M aqueous HCl (1 mL×2). The resulting sold was dried to afford 4-isothiocyanatobenzoic acid in yield of 90%. Methyl 2-cyanoacetate (2 mmol) followed by a solution of 4-isothiocyanatobenzoic acid (2 mmol) in anhydrous DMF (2 mL) were added to a cold suspension of powdered KOH (4 mmol) in dry DMF (2 mL). The mixture was stirred at room temperature for 0.5 h, then cooled again to 0 °C, treated with a solution of 2-chloroacetyl chloride (3 mmol) in anhydrous DMF (2 mL) and stirred at room temperature overnight. The mixture was poured into ice-cold water, and the resulting precipitate was filtered off, dried, and crystallized from DCM-EtOH to give intermediate **IM** in yield of 68%. Mp 290.1-290.7 °C. ^1^H-NMR (DMSO*-d*_6_): δ 13.28 (s, 1H), 8.06 (d, *J* = 8.4 Hz, 2H), 7.56 (d, *J* = 8.4 Hz, 2H), 4.08 (s, 2H), 3.71 (s, 3H) ppm. ^13^C-NMR (DMSO-*d***_6_**): δ 173.30, 172.30, 166.57, 165.31, 138.53, 132.45, 130.22, 129.71, 112.32, 75.76, 52.38, 32.27 ppm. HRMS (EI) calc. for C_12_H_8_N_2_O_3_S^+^ 318.0310; found 318.0312.

##### General Procedure for the Synthesis of Intermediates **40b** and **41b**

TEA (15 mmol) followed by 4-nitrobenzoyl chloride (10 mmol) was dropwise added to a solution of compound **40a**, **41a** in THF (40 mL) in ice bath. The mixture was allowed to stir at room temperature overnight. Then the mixture was diluted with water (50 mL), acidified with 1 M HCl, filtered. The filter cake was washed with water, crystallized from DCM-MeOH to give intermediate **40b**, **41b**.

*3-(4-Nitrobenzamido)benzoic acid* (**41b**): ^1^H-NMR (DMSO-*d*_6_): δ 10.80 (s, 1H), 8.43 (s, 1H), 8.23 (d, *J* = 8.8 Hz, 2H), 8.23 (d, *J* = 8.8 Hz, 2H), 8.06 (d, *J* = 8.0 Hz, 1H), 7.73 (d, *J* = 8.0 Hz, 1H), 7.52 (t, *J* = 8.0 Hz, 1H) ppm; ^13^C-NMR (DMSO-*d*_6_): δ 167.08, 164.04, 149.19, 140.22, 138.91, 131.25, 129.25, 128.98, 124.92, 124.52, 123.53, 121.21 ppm.

##### General Procedure for the Synthesis of Intermediates **40c** and **41c**

Compounds **40c** and **41c** were prepared from **40b** and **41b** according the same procedure described for **19c**–**22c**.

##### Synthesis of Intermediate **42a**

Phthalide (10 mmol) and potassium phthalimide (11 mmol) was added to DMF (7 mL). The mixture was stirred under reflux condition until the reaction was completed. 35% AcOH (11 mL) was added after cooling to room temperature. The resulted mixture was filtered after stirring for 0.5 h. The filter cake was washed with water and EtOH in order, crystallized from DCM-MeOH to give intermediate **42a** in yield of 56%.

##### Synthesis of Intermediate **42b**

Compound **42a** (5 mmol) was dissolved in DMF (10 mL) and EtOH (20 mL). The mixture was heated to 75 °C, added 80% hydrazine hydrate (0.6 mL) and stirred at this temperature overnight. The mixture was filtered after cooling to room temperature. The filtrate was concentrated to give **42b** in yield of 80%.

##### Synthesis of Intermediate **43a**

Concentrated H_2_SO_4_ (30 mmol) was added to a mixture of magnesium sulfate (120 mmol) and DCM (120 mL). Then 3-cyanobenzoic acid (30 mmol) and *tert*-butanol were added after stirring for 15 min. The mixture was stirred at room temperature for 24 h and filtered. The filtrate was neutralized with saturated NaHCO_3_, diluted with water, and extracted with DCM. The combined organic layer was washed with saturated NaCl, concentrated and purified by column chromatography (PE/EA=3:1, V/V) to give **43a** in yield of 58%. ^1^H-NMR (DMSO-*d*_6_): δ 8.25 (s, 1H), 8.19 (d, *J* = 8.0 Hz, 1H), 8.10 (d, *J* = 8.0 Hz, 1H), 7.72 (t, *J* = 8.0 Hz, 1H), 1.56 (s, 9H) ppm. ^13^C-NMR (DMSO-*d*_6_): δ 163.21, 136.23, 133.41, 132.47, 132.40, 130.04, 117.97, 111.91, 81.87, 27.62 ppm.

##### Synthesis of Intermediate **43b**

A mixture of HCOOH and TEA (25 mL, V:V=5:1) followed by Pd/C (1.1 g) was slowly added to a solution of compound **43a** in THF (25 mL) under Ar atmosphere. The mixture was stirred at 40 °C for 4 h and filtered. The filtrate was neutralized with saturated NaHCO_3_ and concentrated to remove THF. The residue was extracted with EA. The combined organic layer was washed with saturated NaCl, dried (Na_2_SO_4_), and concentrated to give compound **43b**. GC-MS *m/z* 207 [M]^+^.

##### Synthesis of Intermediate **43c**

Compound **43b** (2 mmol), **IM** (2 mmol), and 4-dimethylaminopyridine (DMAP, 20 mg) was added to DCM (20 mL). Then 1-ethyl-(3-dimethylaminopropyl)carbonyldiimide hydrochloride (EDCI, 4 mmol) was added. The mixture was stirred at room temperature until the reaction was completed, and concentrated. The residue was purified by column chromatography (PE/EA=4:3, V/V) to give **43c** in yield of 68%. ^1^H-NMR (DMSO-*d*_6_): δ 9.28 (t, *J* = 6.0 Hz, 1H), 8.02 (d, *J* = 8.8 Hz, 2H), 7.89 (s, 1H), 7.79 (d, *J* = 7.6 Hz, 1H), 7.60 (d, *J* = 8.0 Hz, 1H), 7.54 (d, *J* = 8.8 Hz, 2H), 7.48 (d, *J* = 8.0 Hz, 1H), 4.55 (d, *J* = 6.0 Hz, 2H), 4.09 (s, 2H), 3.71 (s, 3H), 1.54 (s, 9H) ppm.

##### Synthesis of Intermediate **44a**

KMnO_4_ (60 mmol) was added to a solution of 1-(tert-butyl)-2-methylbenzene in *tert*-butanol (60 mL) and water (60 mL). The mixture was stirred under reflux condition until the reaction was completed and filtered. The filtrate was concentrated to remove *tert*-butanol, acidified with concentrated HCl, and filtered. The filter cake was dried to give **44a**, which was directly used in the next step.

##### Synthesis of Intermediate **44b**

Compound **44b** was dissolved in concentrated H_2_SO_4_ (25 mL) and cooled to 0 °C. Then a solution of KNO_3_ (20 mmol) in concentrated H_2_SO_4_ (25 mL) was dropwise added to the mixture. The mixture was reacted at 0 °C until the reaction was completed, and dropwise added to ice water. The resulted mixture was filtered and washed with water. The filter cake was dried and crystallized from EtOH to give intermediate **44b** in yield of 40% over two steps. ^1^H-NMR (DMSO-*d*_6_): δ 13.75 (s, 1H), 8.21 (dd, *J* = 8.8, 2.8 Hz, 1H), 8.10 (d, *J* = 2.8 Hz, 1H), 7.80 (d, *J* = 8.8 Hz, 1H), 1.43 (s, 9H) ppm; ^13^C-NMR (DMSO-*d*_6_): δ 170.88, 153.99, 144.99, 135.54, 128.88, 123.82, 122.76, 36.35, 30.62 ppm.

##### Synthesis of Intermediate **44c**

Compound **44b** (10 mmol) was added to a mixture of EtOH (60 mL) and water (15 mL). Then reduced iron powder (100 mmol) and concentrated HCl (10 drops) were added. The mixture was stirred under reflux condition until the reaction was completed, filtered. The filter cake was washed with EA. The filtrate was concentrated and directly used in the next step.

##### Synthesis of Intermediate **44d**

4-Nitrobenzoyl chloride (6 mmol) was slowly added to a mixture of compound **44c**, DCM (30 mL), and TEA (10 mmol). The obtained mixture was stirred at room temperature until the reaction was completed. The mixture was acidified with 1 M HCl and filtered. The filter cake was washed with water, dried and crystallized from DCM/MeOH to give intermediate **44d** in yield of 40% over two steps. ^1^H-NMR (DMSO-*d*_6_): δ 13.15 (s, 1H), 10.61 (s, 1H), 8.42–8.35 (m, 2H), 8.23–8.17 (m, 2H), 7.81–7.76 (m, 2H), 7.49 (d, *J* = 9.2 Hz, 1H), 1.39 (s, 9H) ppm; ^13^C-NMR (DMSO-*d*_6_): δ 172.65, 163.77, 149.18, 141.93, 140.26, 136.09, 134.46, 129.16, 127.25, 123.54, 120.89, 119.73, 35.23, 31.10 ppm.

##### Synthesis of Intermediate **44e**

Compound **44e** was prepared from **44d** in the same manner as described for **44c**. ^1^H-NMR (DMSO-*d*_6_): δ 13.00 (s, 1H), 9.79 (s, 1H), 7.78–7.66 (m, 4H), 7.45–7.34 (m, 1H), 6.60 (d, *J* = 8.8 Hz, 2H), 5.76 (s, 2H), 1.37 (s, 9H) ppm; ^13^C-NMR (DMSO-*d*_6_): δ 165.20, 152.17, 140.36, 137.12, 129.31, 126.83, 120.79, 120.29, 119.28, 112.50, 79.15, 35.09, 31.18 ppm.

##### Synthesis of Intermediate **45a**

2-Bromonaphthalene (50 mmol) was dissolved in dry DCM under Ar atmosphere and cooled to -10 °C. Then the mixture was added AlCl_3_ (150 mmol) and stirred until it turns green. Then acetyl chloride was added after cooling to -78 °C and stirred at this temperature for 3 h. The mixture was quenched with 1 M HCl, diluted with water, and extracted with DCM. The combined organic phases were washed with 1 M HCl and saturated NaCl in order, dried over Na_2_SO_4_, and the solvent was removed under reduced pressure. The residue was purified by column chromatography (PE/EA=4:1, V/V) to give **45d** in yield of 89%. ^1^H-NMR (CDCl_3_): δ 9.03 (d, *J* = 2.0 Hz, 1H), 7.99 (dd, *J* = 7.2, 1.0 Hz, 1H), 7.96 (d, *J* = 8.4 Hz, 1H), 7.73 (d, *J* = 8.8 Hz, 1H), 7.62 (dd, *J* = 8.8, 2.0 Hz, 1H), 7.52 (dd, *J* = 8.4, 7.2 Hz, 1H), 2.74 (s, 3H) ppm; ^13^C-NMR (CDCl_3_): δ 201.06, 134.13, 133.08, 132.42, 131.13, 130.04, 129.84, 128.57, 124.76, 123.01, 29.76 ppm.

##### Synthesis of Intermediates **45b**

Compound **45a** (20 mmol), ammonia (100 mmol), CuI (4 mmol), L-proline (8 mmol), and K_2_CO_3_ (60 mmol) was suspended in DMSO (40 mL) under Ar atmosphere. The mixture was stirred at 85 °C for 24 h, and filtered after cooling to room temperature. The filter cake was washed with EA. The filtrate was diluted with water and extracted with EA. The combined organic phases were washed with saturated NaCl, dried over Na_2_SO_4_, and the solvent was removed under reduced pressure. The residue was purified by column chromatography (PE/EA=3:1, V/V) to give **45b** in yield of 52%.

##### Synthesis of Intermediates **46a**

DMAP (0.5 mmol) and *tert*-butyl carbazate (6 mmol) were added to a mixture of compound **IM** and DCM (25 mL). Then the mixture was added EDCI (10 mmol) and stirred at room temperature until the reaction was completed. The solvent was removed under reduced pressure and the residue was purified by column chromatography (DCM/acetone=10:1, V/V) to give **46a** in yield of 72%. ^1^H- NMR (CDCl_3_): δ 8.79 (s, 1H), 7.94 (d, *J* = 8.4 Hz, 2H), 7.31 (d, *J* = 8.4 Hz, 2H), 6.86 (s, 1H), 3.91 (s, 2H), 3.81 (s, 3H), 1.50 (s, 9H) ppm; ^13^C-NMR (CDCl_3_): δ 172.20, 170.07, 165.67, 165.62, 155.69, 137.12, 129.25, 129.15, 111.85, 81.99, 78.88, 52.90, 31.79, 28.34, 28.18 ppm.

##### Synthesis of Intermediate **46b**

CF_3_COOH was added to a solution of compound **46a** in DCM (45 mL). The mixture was stirred at room temperature overnight and concentrated to give **46b**, which was directly used in the next step.

##### Synthesis of Aryl Isothiocyanates **9e**–**22e**, **23c**, **24d**

A mixture of 1,4-diazabicyclo(2.2.2) octane (DABCO, 15 mmol), aromatic amines **9c**–**22c**, **23a**, **24b** (5 mmol), and carbon disulfide (25 mL) in acetone (5 mL) was stirred overnight at room temperature. The precipitated solid was filtered. To a mixture of the solid and chloroform (20 mL) at 0 °C, was added dropwise a solution of triphosgene (2 mmol) in chloroform (10 mL) over 30 min. The reaction mixture was allowed to warm to room temperature and stirred overnight. After the resulting mixture was filtered, the filtrate was concentrated under reduced pressure and purified by column chromatography (100% PE) to give **9c**–**22c**, **23a**, **24b** in 70%–95% yield as white solids or colorless oils.

##### Synthesis of Aryl Isothiocyanates **25e, 26e, 40d, 41d, 44f**

Isothiocyanates **25e**, **26e**, **40d**, **41d** and **44f** were prepared from **25d**, **26d**, **40c**, **41c**, **44e** in the same manner as described for 4-isothiocyanatobenzoic acid.

#### 3.2.2. Synthesis of Compounds **9**–**26**, **40**, **41**, **44**

Compounds **9**–**26**, **40**, **41** and **44** were prepared from **9e**–**22e**, **23c**, **24d**, **25e**, **26e**, **40d**, **41d**, **44f** in the same manner as described for compound **IM**.

*Methyl (Z)-2-(3-([1,1’-biphenyl]-4-yl)-4-oxothiazolidin-2-ylidene)-2-cyanoacetate* (**9**). Mp 235.1–235.5 °C. ^1^H-NMR (DMSO-*d*_6_): *δ* 7.82 (d, *J* = 8.4 Hz, 2H), 7.75 (d, *J* = 7.2 Hz, 2H), 7.51 (t, *J* = 8.0 Hz, 4H), 7.43 (t, *J* = 7.2 Hz, 1H), 4.10 (s, 2H), 3.72 (s, 3H) ppm. HRMS (ES+) calcd for C_19_H_14_N_2_O_3_S (M + H)^+^,351.0803; found, 351.0802.

*Methyl (Z)-2-cyano-2-(3-(3-fluoro-[1,1’-biphenyl]-4-yl)-4-oxothiazolidin-2-ylidene)acetate* (**10**). Mp 180.0–180.1 °C. ^1^H-NMR (DMSO-*d*_6_): δ 7.83–7.79 (m, 3H), 7.72 (dd, *J*_1_ = 8.4 Hz, *J_2_* = 2.0 Hz, 1H), 7.66 (t, *J* = 7.6 Hz, 1H), 7.56–7.43 (m, 3H), 4.24 (ABq, *J_gem_* = 18.8 Hz, 2H), 3.74 (s, 3H) ppm. ^19^F-NMR (DMSO-*d*_6_): δ −121.94 (t, *J* = 9.4 Hz) ppm. HRMS (ES+) calcd for C_19_H_13_FN_2_O_3_S (M + H)^+^,369.0703; found, 369.0709.

*Methyl (Z)-2-cyano-2-(3-(3,5-difluoro-[1,1’-biphenyl]-4-yl)-4-oxothiazolidin-2-ylidene)acetate* (**11**). Mp 185.6–186.0 °C. ^1^H-NMR (DMSO-*d*_6_): δ 7.82 (dd, *J_1_* = 16.8 Hz, *J_2_* = 6.8 Hz, 4H), 7.55–7.47 (m, 3H), 4.40 (s, 2H), 3.76 (s, 3H) ppm. ^19^F-NMR (DMSO-*d*_6_): δ −118.44 (d, *J* = 11.1 Hz) ppm. HRMS (ES+) calcd for C_19_H_12_F_2_N_2_O_3_S (M + H)^+^,387.0615; found, 387.0621.

*Methyl (Z)-2-cyano-2-(3-(3-fluoro-2’-methyl-[1,1’-biphenyl]-4-yl)-4-oxothiazolidin-2-ylidene)acetate* (**12**). Mp 198.6–198.9 °C. ^1^H-NMR (400 MHz, DMSO-*d*_6_): δ 7.63 (t, *J* = 8.0 Hz, 1H), 7.44 (d, *J* = 10.8 Hz, 1H), 7.33–7.26 (m, 5H), 4.25 (ABq, *J_gem_* = 18.8 Hz, 2H), 3.75 (s, 3H), 2.26 (s, 3H) ppm. ^19^F NMR (376 MHz, DMSO-*d*_6_): δ −122.72 (t, *J* = 9.4 Hz) ppm. HRMS (ES+) calcd for C_20_H_15_FN_2_O_3_S (M + H)^+^, 383.0866; found, 383.0868.

*Methyl (Z)-2-cyano-2-(3-(3-fluoro-2’-methoxy-[1,1’-biphenyl]-4-yl)-4-oxothiazolidin-2-ylidene)acetate* (**13**). Mp 220.1–220.5 °C. ^1^H-NMR (DMSO-*d*_6_): δ 7.59 (t, *J* = 8.0 Hz, 2H), 7.50 (dd, *J*_1_ = 8.4 Hz, *J*_2_ = 1.6Hz, 1H), 7.43 (td, *J*_1_ = 8.0Hz, *J*_2_ = 1.6 Hz, 1H), 7.37 (dd, *J*_1_ = 7.2 Hz, *J*_2_ = 1.6Hz, 1H), 7.18 (d, *J* = 8.0 Hz,1H), 7.08 (t, *J* = 7.4 Hz, 1H), 4.24 (ABq, *J_gem_* = 18.8 Hz, 2H), 3.82 (s, 3H), 3.75 (s, 3H) ppm. ^19^F-NMR (DMSO-*d*_6_): δ −123.08 (dd, *J*_1_ = 12.8 Hz, *J*_2_ = 8.5 Hz) ppm. HRMS (ES+) calcd for C_20_H_15_FN_2_O_4_S (M + H)^+^, 399.0815; found, 399.0814.

*Methyl (Z)-2-cyano-2-(3-(3-fluoro-3’-methoxy-[1,1’-biphenyl]-4-yl)-4-oxothiazolidin-2-ylidene)acetate* (**14**). Mp 193.1–193.6 °C. ^1^H-NMR (DMSO-*d*_6_): δ 7.84 (dd, *J*_1_ = 11.6 Hz, *J*_2_ = 1.6Hz, 1H), 7.73 (dd, *J*_1_ = 8.4 Hz, *J*_2_ = 1.6Hz, 1H), 7.65 (t, *J* = 8.0 Hz, 1H), 7.43 (t, *J* = 8.0 Hz, 1H), 7.35 (t, *J* = 8.0 Hz,2H), 7.03 (dd, *J_1_* = 8.0 Hz, *J_2_* = 1.6 Hz, 1H), 4.24 (ABq, *J_gem_* = 18.8 Hz, 2H), 3.86 (s, 3H), 3.75 (s, 3H) ppm. ^19^F-NMR (DMSO-*d*_6_): δ −121.97 (dd, *J_1_* = 12.8 Hz, *J_2_* = 9.4 Hz) ppm. HRMS (ES+) calcd for C_20_H_15_FN_2_O_4_S (M + H)^+^, 399.0815; found, 399.0821.

*Methyl (Z)-2-cyano-2-(3-(3-fluoro-4’-methoxy-[1,1’-biphenyl]-4-yl)-4-oxothiazolidin-2-ylidene)acetate* (**15**). Mp 177.1–177.3 °C. ^1^H-NMR (DMSO-*d*_6_): δ 7.77–7.72 (m, 3H), 7.65 (d, *J* = 8.8 Hz, 1H), 7.59 (t, *J* = 8.0 Hz, 1H), 7.07 (d, *J* = 8.8 Hz, 2H), 4.22 (ABq, *J_gem_* = 18.6 Hz, 2H), 3.83 (s, 3H), 3.74 (s, 3H) ppm. ^19^F-NMR (DMSO-*d*_6_): δ −122.12 (dd, *J_1_* = 13.2 Hz, *J_2_* = 8.9 Hz) ppm. HRMS (ES+) calcd for C_20_H_15_FN_2_O_4_S (M + H)^+^, 399.0815; found, 399.0816.

*Methyl (Z)-2-cyano-2-(3-(3’-cyano-3-fluoro-[1,1’-biphenyl]-4-yl)-4-oxothiazolidin-2-ylidene)acetate* (**16**). Mp 191.2–191.3 °C. ^1^H-NMR (DMSO-*d*_6_): δ 8.33 (s, 1H), 8.16 (d, *J* = 8.0 Hz, 1H), 7.95 (dd, *J_1_* = 11.2 Hz, *J_2_* = 7.6 Hz, 2H), 7.83 (d, *J* = 8.4 Hz, 1H), 7.72 (q, *J* = 7.6 Hz, 2H), 4.24 (ABq, *J_gem_* = 18.8 Hz, 2H), 3.75 (s, 3H) ppm. ^19^F-NMR (DMSO-*d*_6_): δ −121.44 (td, *J_1_* = 8.9 Hz, *J_2_* = 3.4 Hz) ppm. HRMS (ES+) calcd for C_20_H_12_FN_3_O_3_S (M + H)^+^, 394.0662; found, 394.0668.

*Methyl (Z)-2-cyano-2-(3-(3-fluoro-3’-nitro-[1,1’-biphenyl]-4-yl)-4-oxothiazolidin-2-ylidene) acetate* (**17**). Mp 179.2–179.9 °C. ^1^H-NMR (DMSO-*d*_6_): δ 8.57–8.56 (m, 1H), 8.32–8.26 (m, 2H), 8.02 (dd, *J_1_* = 11.2 Hz, *J_2_* = 2.0 Hz, 1H), 7.88–7.80 (m, 2H), 7.74 (t, *J* = 8.0 Hz, 1H), 4.24 (ABq, *J_gem_* = 18.4 Hz, 2H), 3.75 (s, 3H). ^19^F-NMR (DMSO-*d*_6_): δ −121.31 (t, *J* = 10.6 Hz). HRMS (ES+) calcd for C_19_H_12_FN_3_O_5_S (M + H)^+^, 413.3851; found, 413.3853.

*Methyl (Z)-2-cyano-2-(3-(3-fluoro-3’-(trifluoromethyl)-[1,1’-biphenyl]-4-yl)-4-oxothiazolidin-2-ylidene) -acetate* (**18**). Mp 182.4–183.1 °C. ^1^H-NMR (DMSO-d_6_): δ 8.12 (s, 1H), 7.98 (d, *J* = 11.2 Hz, 1H), 7.83 (dd, *J_1_* = 8.4 Hz, *J_2_* = 3.2Hz, 2H), 7.78–7.70 (m, 2H), 4.18 (ABq, *J_gem_* = 18.8 Hz, 2H), 3.75 (s, 3H) ppm. ^19^F-NMR (DMSO-*d*_6_): δ −61.03 (s), -121.55(dd, *J_1_* = 12.3 Hz, *J_2_* = 8.9 Hz) ppm. HRMS (ES+) calcd for C_20_H_12_F_4_N_2_O_3_S (M + H)^+^, 437.0583; found, 437.0591.

*Methyl (Z)-2-cyano-2-(4-oxo-3-(4-phenoxyphenyl)thiazolidin-2-ylidene)acetate* (**19**). White solid, Yield: 72%. Mp 167.6–168.3 °C; ^1^H-NMR (DMSO-*d*_6_): δ 7.46–7.42 (m, 2H), 7.39 (t, *J* = 8.0 Hz, 2H), 7.18–7.12 (m, 3H), 7.07 (d, *J* = 8.0 Hz, 2H), 4.07 (s, 2H), 3.71 (s, 3H) ppm. ^13^C-NMR (DMSO-*d*_6_): δ 173.40, 173.13, 165.44, 157.97, 156.72, 131.34, 130.08, 130.01, 123.56, 119.73, 118.35, 112.40, 75.73, 52.35, 32.17 ppm. HRMS (EI) calc. for C_19_H_14_N_2_O_4_S^+^ 366.0674; found 366.0675.

*Methyl (Z)-2-cyano-2-(3-(4-(2-methoxyphenoxy)phenyl)-4-oxothiazolidin-2-ylidene)acetate* (**20**). White solid, Yield: 74%. Mp 181.2–182.1 °C; ^1^H-NMR (DMSO-*d*_6_): δ 7.34 (d, *J* = 8.8 Hz, 2H), 7.25–7.16 (m, 2H), 7.05 (dd, *J* = 8.0, 1.2 Hz, 1H), 6.99–6.93 (m, 3H), 4.05 (s, 2H), 3.75 (s, 3H), 3.71 (s, 3H) ppm. ^13^C-NMR (DMSO-*d*_6_): δ 173.48, 173.02, 165.48, 159.03, 151.09, 143.57, 130.83, 128.87, 125.66, 121.13, 121.04, 117.08, 113.45, 112.28, 75.80, 55.61, 52.32, 32.08 ppm. HRMS (EI) calc. for C_20_H_16_N_2_O_5_S^+^ 396.0780; found 396.0779.

*Methyl (Z)-2-cyano-2-(4-oxo-3-(4-(2-(trifluoromethyl)phenoxy)phenyl)thiazolidin-2-ylidene)acetate* (**21**). White solid, Yield: 72%.Mp 159.7–161.3 °C; ^1^H-NMR (DMSO-*d*_6_): δ 7.61 (t, *J* = 8.2 Hz, 1H), 7.52 (d, *J* = 8.8 Hz, 2H), 7.48 (d, *J* = 8.0 Hz, 1H), 7.37 (d, *J* = 6.4 Hz, 2H), 7.27 (d, *J* = 8.8 Hz, 2H), 4.08 (s, 2H), 3.72 (s, 3H) ppm. ^13^C-NMR (DMSO-*d*_6_): δ 173.35, 173.07, 165.42, 157.74, 156.76, 131.78, 131.23, 131.16, 130.77 (q, ^2^*J*_CF_ = 31.9 Hz), 123.65 (q, ^1^*J*_CF_ = 270.9 Hz), 121.75, 120.84, 119.73 (q, ^3^*J*_CF_ = 3.8 Hz), 114.15 (q, ^3^*J*_CF_ = 3.8 Hz), 112.42, 75.79, 52.35, 32.20 ppm. ^19^F-NMR (DMSO-*d*_6_): δ −61.13 (s, 3F) ppm. HRMS (EI) calc. for C_20_H_13_F_3_N_2_O_4_S^+^ 434.0548; found 434.0550.

*Methyl (Z)-2-(3-(4-(4-(tert-butyl)phenoxy)phenyl)-4-oxothiazolidin-2-ylidene)-2-cyanoacetate* (**22**). White solid, Yield: 74%. Mp 212.9–213.9 °C; ^1^H-NMR (DMSO-*d*_6_): δ 7.41 (d, *J* = 8.4 Hz, 2H), 7.40 (d, *J* = 8.4 Hz, 2H), 7.11 (d, *J* = 8.8 Hz, 2H), 7.00 (d, *J* = 8.8 Hz, 2H), 4.07 (s, 2H), 3.71 (s, 3H), 1.28 (s, 9H) ppm. ^13^C-NMR (DMSO-*d*_6_): δ 173.40, 173.11, 165.45, 158.35, 154.28, 145.96, 131.24, 129.81, 126.67, 119.40, 118.05, 112.35, 75.76, 52.34, 34.03, 32.15, 31.21 ppm. HRMS (EI) calc. for C_23_H_22_N_2_O_4_S^+^ 422.1300; found 422.1299.

*Methyl (Z)-2-cyano-2-(4-oxo-3-(3-phenoxyphenyl)thiazolidin-2-ylidene)acetate* (**23**). White solid, Yield: 76%. Mp 183.4–184.3 °C; ^1^H-NMR (DMSO-*d*_6_): δ 7.52 (t, *J* = 8.0 Hz, 1H), 7.39 (t, *J* = 8.0 Hz, 2H), 7.25–7.20 (m, 2H), 7.17–7.12 (m, 2H), 7.10 (d, *J* = 8.0 Hz, 2H), 4.01 (ABq, *J_gem_* = 18.4 Hz, 2H), 3.71 (s, 3H) ppm. ^13^C-NMR (DMSO-*d*_6_): δ 173.25, 172.68, 165.40, 157.06, 156.64, 136.07, 130.69, 130.04, 124.60, 123.63, 121.35, 119.87, 118.48, 112.51, 75.57, 52.36, 32.19 ppm. HRMS (EI) calc. for C_19_H_14_N_2_O_4_S^+^ 366.0674; found 366.0675.

*Methyl (Z)-2-(3-(4-(benzyloxy)phenyl)-4-oxothiazolidin-2-ylidene)-2-cyanoacetate* (**24**). White solid, Yield: 75%. Mp 202.0–202.8 °C; ^1^H-NMR (DMSO-*d*_6_): δ 7.47 (d, *J* = 7.2 Hz, 2H), 7.40 (t, *J* = 7.2 Hz, 2H), 7.37–7.34 (m, 1H), 7.31 (dt, *J* = 8.8, 2.6 Hz, 2H), 7.11 (dt, *J* = 8.8, 2.6 Hz, 2H), 5.15 (s, 2H), 4.05 (s, 2H), 3.70 (s, 3H) ppm. ^13^C-NMR (DMSO-*d*_6_): δ 173.51, 173.12, 165.51, 159.73, 136.62, 130.54, 128.44, 127.91, 127.74, 127.48, 115.18, 112.24, 75.74, 69.49, 52.31, 32.00 ppm. HRMS (EI) calc. for C_20_H_16_N_2_O_4_S^+^ 380.0831; found 380.0834.

*(Z)-2-((4-(2-(1-Cyano-2-methoxy-2-oxoethylidene)-4-oxothiazolidin-3-yl)phenoxy)methyl)benzoic acid* (**25**). White solid, Yield: 67%. Mp 210.6–211.5 °C; ^1^H-NMR (DMSO-*d*_6_): δ 13.13 (s, 1H), 7.95 (d, *J* = 7.6 Hz, 1H), 7.65 (d, *J* = 7.6 Hz, 1H), 7.59 (t, *J* = 7.6 Hz, 1H), 7.46 (t, *J* = 7.6 Hz, 1H), 7.31 (d, *J* = 8.8 Hz, 2H), 7.07 (d, *J* = 8.8 Hz, 2H), 5.52 (s, 2H), 4.05 (s, 2H), 3.71 (s, 3H) ppm. ^13^C-NMR (DMSO-*d*_6_): δ 173.50, 173.12, 168.05, 165.50, 159.68, 137.94, 132.17, 130.62, 130.50, 129.27, 127.80, 127.69, 127.57, 115.19, 112.22, 75.78, 67.88, 52.30, 32.02 ppm. HRMS (EI) calc. for C_21_H_16_N_2_O_6_S^+^ 424.0729; found 424.0728.

*(Z)-3-((4-(2-(1-Cyano-2-methoxy-2-oxoethylidene)-4-oxothiazolidin-3-yl)phenoxy)methyl)benzoic acid* (**26**). White solid, Yield: 68%. Mp 233.2–234.1 °C; ^1^H-NMR (DMSO-*d*_6_): δ 13.02 (s, 1H), 8.06 (s, 1H), 7.92 (d, *J* = 7.6 Hz, 1H), 7.72 (d, *J* = 7.6 Hz, 1H), 7.54 (t, *J* = 7.6 Hz, 1H), 7.32 (d, *J* = 8.8 Hz, 2H), 7.13 (d, *J* = 8.8 Hz, 2H), 5.24 (s, 2H), 4.05 (s, 2H), 3.71 (s, 3H) ppm. ^13^C-NMR (DMSO-*d*_6_): δ 173.48, 173.07, 167.08, 165.50, 159.58, 137.24, 131.97, 130.99, 130.58, 128.78, 128.38, 127.63, 115.23, 112.23, 75.78, 68.94, 52.30, 32.00 ppm. HRMS (EI) calc. for C_21_H_16_N_2_O_6_S^+^ 424.0729; found 424.0730.

*(Z)-2-(4-(2-(1-Cyano-2-methoxy-2-oxoethylidene)-4-oxothiazolidin-3-yl)benzamido)benzoic acid* (**40**). White solid, Yield: 70%. Mp 261.8–262.5 °C; ^1^H-NMR (DMSO-*d*_6_): δ 13.81 (s, 1H), 12.25 (s, 1H), 8.71 (d, *J* = 8.0 Hz, 1H), 8.14–8.06 (m, 3H), 7.74–7.63 (m, 3H), 7.24 (t, *J* = 7.4 Hz, 1H), 4.11 (s, 2H), 3.73 (s, 3H) ppm. ^13^C-NMR (DMSO-*d*_6_): δ 173.33, 172.24, 169.94, 165.31, 162.25, 140.80, 137.96, 136.10, 134.28, 131.26, 130.10, 128.01, 123.20, 120.06, 116.84, 112.36, 75.90, 52.37, 32.25, 30.73 ppm. HRMS (ESI) calc. for C_21_H_15_N_3_O_6_SNa^+^ (M + Na)^+^, 460.0579; found 460.0578. HRMS (EI) calc. for C_21_H_16_N_2_O_6_S^+^ 424.0729; found 424.0728.

*(Z)-3-(4-(2-(1-Cyano-2-methoxy-2-oxoethylidene)-4-oxothiazolidin-3-yl)benzamido)benzoic acid* (**41**). White solid, Yield: 68%. Mp 205.7–206.6 °C; ^1^H-NMR (DMSO-*d*_6_): δ 12.98 (s, 1H), 10.57 (s, 1H), 8.45 (s, 1H), 8.13 (d, *J* = 7.6 Hz, 2H), 8.07 (d, *J* = 8.0 Hz, 1H), 7.71 (d, *J* = 7.6 Hz, 1H), 7.61 (d, *J* = 7.6 Hz, 2H), 7.50 (t, *J* = 7.8 Hz, 1H), 4.10 (s, 2H), 3.72 (s, 3H) ppm. ^13^C-NMR (DMSO-*d*_6_): δ 173.33, 172.24, 169.94, 165.31, 162.25, 140.80, 137.96, 136.10, 134.28, 131.26, 130.10, 128.01, 123.20, 120.06, 116.84, 112.36, 75.90, 52.37, 32.25, 30.73 ppm. HRMS (ESI) calc. for C_21_H_15_N_3_O_6_SNa^+^ (M + Na)+, 460.0579; found 460.0580.

*(Z)-2-(tert-butyl)-5-(4-(2-(1-cyano-2-methoxy-2-oxoethylidene)-4-oxothiazolidin-3-yl)benzamido)benzoic acid* (**44**). White solid, Yield: 69%. Mp 328.1–328.9 °C; ^1^H-NMR (DMSO-*d*_6_): δ 13.13 (s, 1H), 10.44 (s, 1H), 8.10 (d, *J* = 8.8 Hz, 2H), 7.80 (dd, *J* = 8.4, 2.4 Hz, 1H), 7.78 (d, *J* = 2.4 Hz, 1H), 7.60 (d, *J* = 8.8 Hz, 2H), 7.48 (d, *J* = 8.4 Hz, 1H), 4.10 (s, 2H), 3.72 (s, 3H), 1.39 (s, 9H) ppm. ^13^C-NMR (DMSO-*d*_6_): δ 173.39, 172.71, 172.32, 165.35, 164.39, 141.59, 137.55, 136.35, 136.04, 134.42, 129.47, 128.66, 127.19, 120.88, 119.69, 112.38, 75.79, 52.40, 35.21, 32.23, 31.12 ppm. HRMS (EI) calc. for C_25_H_23_N_3_O_6_S^+^ 493.1308; found 493.1300.

#### 3.2.3. Synthesis of Compounds **27**–**39**, **45**

General procedure for the synthesis of compounds **27–39, 45**

Corresponding aromatic amines (1.2 mmol) and DMAP (0.1 mmol) were added to a mixture of compound **IM** (1 mmol) and DCM (5 mL). Then the mixture was added EDCI (2 mmol) and stirred at room temperature until the reaction was completed. The solvent was removed under reduced pressure and the residue was purified by column chromatography (DCM/acetone=10:1, V/V) to give compounds **27**–**39**, **45**.

*Methyl (Z)-2-cyano-2-(3-(4-(cyclohexylcarbamoyl)phenyl)-4-oxothiazolidin-2-ylidene)acetate* (**27**). White solid, Yield: 78%. Mp 224.3–225.1 °C; ^1^H-NMR (DMSO-*d*_6_): δ 7.33 (d, *J* = 8.4 Hz, 2H), 7.26 (d, *J* = 8.4 Hz, 2H), 4.06 (s, 2H), 3.69 (s, 3H), 2.57 (t, *J* = 9.6 Hz, 1H), 1.80 (d, *J* = 9.6 Hz, 4H), 1.71 (d, *J* = 12.4 Hz, 1H), 1.48–1.31 (m, 4H), 1.30–1.18 (m, 1H) ppm. ^13^C-NMR (DMSO-*d*_6_): δ 173.46, 172.71, 165.50, 149.98, 132.44, 128.97, 127.46, 111.95, 75.90, 54.87, 52.30, 43.51, 33.80, 32.06, 26.22, 25.52 ppm. HRMS (EI) calc. for C_20_H_21_N_3_O_4_S^+^ 399.1253; found 399.1251.

*Methyl (Z)-2-cyano-2-(4-oxo-3-(4-(phenylcarbamoyl)phenyl)thiazolidin-2-ylidene)acetate (***28***)*. White solid, Yield: 48%. Mp 194.9–195.6 °C; ^1^H-NMR (DMSO-*d*_6_): δ 10.40 (s, 1H), 8.11 (d, *J* = 8.4 Hz, 2H), 7.80 (d, *J* = 8.0 Hz, 2H), 7.60 (d, *J* = 8.4 Hz, 2H), 7.37 (t, *J* = 7.8 Hz, 2H), 7.13 (t, *J* = 7.2 Hz, 1H), 4.10 (s, 2H), 3.72 (s, 3H) ppm. ^13^C-NMR (DMSO-*d*_6_): δ 173.41, 172.35, 165.37, 164.44, 138.93, 137.47, 136.33, 129.45, 128.67, 128.62, 123.87, 120.49, 112.39, 75.79, 52.41, 32.24 ppm. HRMS (EI) calc. for C_20_H_15_N_3_O_4_S^+^ 393.0783; found 393.0782.

*Methyl (Z)-2-cyano-2-(4-oxo-3-(4-(o-tolylcarbamoyl)phenyl)thiazolidin-2-ylidene)acetate* (**29**). White solid, Yield: 50%. Mp 274.1–275.0 °C; ^1^H-NMR (DMSO-*d*_6_): δ 10.05 (s, 1H), 8.12 (d, *J* = 8.4 Hz, 2H), 7.59 (d, *J* = 8.4 Hz, 2H), 7.36 (d, *J* = 7.6 Hz, 1H), 7.29 (d, *J* = 7.2 Hz, 1H), 7.24 (t, *J* = 6.8 Hz, 1H), 7.19 (t, *J* = 7.4 Hz, 1H), 4.10 (s, 2H), 3.72 (s, 3H), 2.25 (s, 3H) ppm. ^13^C-NMR (DMSO-*d*_6_): δ 173.44, 172.35, 165.38, 164.28, 137.45, 136.17, 135.95, 133.82, 130.33, 129.47, 128.62, 126.70, 126.16, 126.03, 112.43, 75.78, 52.41, 30.67, 17.89 ppm. HRMS (EI) calc. for C_21_H_17_N_3_O_4_S^+^ 407.0940; found 407.0941.

*Methyl (Z)-2-cyano-2-(3-(4-((4-fluorophenyl)carbamoyl)phenyl)-4-oxothiazolidin-2-ylidene)acetate* (**30**). White solid, Yield: 43%. Mp 231.7–232.5 °C; ^1^H-NMR (DMSO-*d*_6_): δ 10.46 (s, 1H), 8.10 (d, *J* = 8.4 Hz, 2H), 7.82 (dd, *J* = 8.8, 5.0 Hz, 2H), 7.60 (d, *J* = 8.4 Hz, 2H), 7.22 (t, *J* = 8.8 Hz, 2H), 4.10 (s, 2H), 3.72 (s, 3H) ppm. ^13^C-NMR (DMSO-*d*_6_): δ 173.40, 172.34, 165.36, 164.38, 158.39 (d, ^1^*J*_CF_ = 239.1 Hz), 137.51, 136.17, 135.28 (d, ^4^*J*_CF_ = 2.5 Hz), 129.47, 128.65, 122.33 (d, ^3^*J*_CF_ = 7.8 Hz), 115.21 (d, ^2^*J*_CF_ = 22.1 Hz), 112.40, 75.79, 52.40, 32.24 ppm. ^19^F-NMR (DMSO-*d*_6_): δ −118.51 – −118.61 (m, 1F) ppm. HRMS (EI) calc. for C_20_H_14_FN_3_O_4_S^+^ 411.0689; found 411.0690.

*Methyl (Z)-2-cyano-2-(3-(4-((3-cyano-4-fluorophenyl)carbamoyl)phenyl)-4-oxothiazolidin-2-ylidene)acetate* (**31**). White solid, Yield: 40%. Mp 264.2–265.1 °C; ^1^H-NMR (DMSO-*d*_6_): δ 10.75 (s, 1H), 8.31 (dd, *J* = 5.8, 2.6 Hz, 1H), 8.11 (d, *J* = 8.4 Hz, 3H), 7.63 (d, *J* = 8.4 Hz, 2H), 7.57 (t, *J* = 9.2 Hz, 1H), 4.10 (s, 2H), 3.73 (s, 3H) ppm. ^13^C-NMR (DMSO-*d*_6_): δ 173.39, 172.30, 165.34, 164.80, 158.49 (d, ^1^*J*_CF_ = 251.2 Hz), 137.85, 136.05 (d, ^3^*J*_CF_ = 6.5 Hz), 135.57, 129.61, 128.76, 127.79 (d, ^3^*J*_CF_ = 8.1 Hz), 124.38, 116.99 (d, ^2^*J*_CF_ = 20.4 Hz), 113.95, 112.42, 99.85 (d, ^2^*J*_CF_ = 16.1 Hz), 75.79, 52.41, 32.25 ppm. ^19^F-NMR (DMSO-*d*_6_): δ −114.31–−114.38 (m, 1F) ppm. HRMS (EI) calc. for C_21_H_13_FN_4_O_4_S^+^ 436.0642; found 436.0640.

*Methyl (Z)-2-(3-(4-((4-chlorophenyl)carbamoyl)phenyl)-4-oxothiazolidin-2-ylidene)-2-cyanoacetate* (**32**). White solid, Yield: 44%. Mp 280.1–280.9 °C; ^1^H-NMR (DMSO-*d*_6_): δ 10.53 (s, 1H), 8.10 (d, *J* = 8.4 Hz, 2H), 7.85 (d, *J* = 8.8 Hz, 2H), 7.61 (d, *J* = 8.4 Hz, 2H), 7.43 (d, *J* = 8.8 Hz, 2H), 4.10 (s, 2H), 3.72 (s, 3H) ppm. ^13^C-NMR (DMSO-*d*_6_): δ 173.40, 172.33, 165.36, 164.57, 137.92, 137.60, 136.07, 129.50, 128.72, 128.55, 127.49, 121.96, 112.40, 75.79, 52.41, 32.24 ppm. HRMS (EI) calc. for C_20_H_14_^35^ClN_3_O_4_S^+^ 427.0394; found 427.0393; calc. for C_20_H_14_^37^ClN_3_O_4_S^+^ 429.0364; found 429.0369.

*Methyl (Z)-2-(3-(4-((4-bromophenyl)carbamoyl)phenyl)-4-oxothiazolidin-2-ylidene)-2-cyanoacetate* (**33**). White solid, Yield: 42%. Mp 306.2–307.1 °C; ^1^H-NMR (DMSO-*d*_6_): δ 10.53 (s, 1H), 8.10 (d, *J* = 8.4 Hz, 2H), 7.80 (d, *J* = 8.8 Hz, 2H), 7.61 (d, *J* = 8.4 Hz, 2H), 7.57 (d, *J* = 8.8 Hz, 2H), 4.10 (s, 2H), 3.73 (s, 3H) ppm. ^13^C-NMR (DMSO-*d*_6_): δ 173.39, 172.32, 165.36, 164.58, 138.35, 137.61, 136.07, 131.46, 129.50, 128.73, 122.32, 115.58, 112.40, 75.80, 52.41, 32.25 ppm. HRMS (EI) calc. for C_20_H_14_^79^BrN_3_O_4_S^+^ 470.9888; found 470.9887; calc. for C_20_H_14_^81^BrN_3_O_4_S ^+^ 472.9868; found 472.9863.

*Methyl (Z)-2-cyano-2-(3-(4-((4-iodophenyl)carbamoyl)phenyl)-4-oxothiazolidin-2-ylidene)acetate* (**34**). White solid, Yield: 45%. Mp 315.8–316.6 °C; ^1^H-NMR (DMSO-*d*_6_): δ 10.49 (s, 1H), 8.09 (d, *J* = 8.8 Hz, 2H), 7.72 (d, *J* = 8.8 Hz, 2H), 7.66 (d, *J* = 8.8 Hz, 2H), 7.60 (d, *J* = 8.8 Hz, 2H), 4.10 (s, 2H), 3.72 (s, 3H) ppm. ^13^C-NMR (DMSO-*d*_6_): δ 173.39, 172.32, 165.36, 164.56, 138.82, 137.59, 137.30, 136.09, 129.49, 128.72, 122.56, 112.40, 87.64, 75.79, 52.42, 32.25 ppm. HRMS (EI) calc. for C_20_H_14_IN_3_O_4_S^+^ 518.9750; found 518.9751.

*Methyl (Z)-2-cyano-2-(4-oxo-3-(4-((4-(trifluoromethyl)phenyl)carbamoyl)phenyl)thiazolidin-2-ylidene) acetate* (**35**). White solid, Yield: 40%. Mp 264.2–265.1 °C; ^1^H-NMR (DMSO-*d*_6_): δ 10.75 (s, 1H), 8.13 (d, *J* = 8.0 Hz, 2H), 8.05 (d, *J* = 8.4 Hz, 2H), 7.76 (d, *J* = 8.4 Hz, 2H), 7.63 (d, *J* = 8.0 Hz, 2H), 4.11 (s, 2H), 3.73 (s, 3H) ppm. ^13^C-NMR (DMSO-_d6_): δ 173.40, 172.33, 165.36, 165.00, 142.61, 137.78, 135.87, 129.55, 128.85, 125.93 (q, ^3^*J*_CF_ = 3.6 Hz), 124.35 (q, ^1^*J*_CF_ = 269.5 Hz), 123.80 (q, ^2^*J*_CF_ = 31.8 Hz), 120.23, 112.42, 75.78, 52.40, 32.25 ppm. ^19^F-NMR (DMSO-*d*_6_): δ −60.35 (s, 3F) ppm. HRMS (EI) calc. for C_21_H_14_F_3_N_3_O_4_S^+^ 461.0657; found 461.0658.

*Methyl (Z)-2-cyano-2-(3-(4-((4-methoxyphenyl)carbamoyl)phenyl)-4-oxothiazolidin-2-ylidene)acetate* (**36**). White solid, Yield: 55%. Mp 268.9–269.7 °C; ^1^H-NMR (DMSO-*d*_6_): δ 10.29 (s, 1H), 8.10 (d, *J* = 7.2 Hz, 2H), 7.71 (d, *J* = 8.0 Hz, 2H), 7.59 (d, *J* = 7.2 Hz, 2H), 6.95 (d, *J* = 8.0 Hz, 2H), 4.10 (s, 2H), 3.76 (s, 3H), 3.73 (s, 3H) ppm. ^13^C-NMR (DMSO-*d*_6_): δ 173.37, 172.31, 165.36, 163.96, 155.69, 137.32, 136.40, 131.97, 129.39, 128.53, 122.10, 113.76, 112.35, 75.83, 55.17, 52.38, 32.21 ppm. HRMS (EI) calc. for C_21_H_17_N_3_O_5_S^+^ 423.0889; found 423.0891.

*Methyl (Z)-2-(3-(4-((4-(tert-butyl)phenyl)carbamoyl)phenyl)-4-oxothiazolidin-2-ylidene)-2-cyanoacetate* (**37**). White solid, Yield: 51%. Mp 256.1–257.0 °C; ^1^H-NMR (DMSO-*d*_6_): δ 10.34 (s, 1H), 8.10 (d, *J* = 8.4 Hz, 2H), 7.71 (d, *J* = 8.8 Hz, 2H), 7.59 (d, *J* = 8.4 Hz, 2H), 7.39 (d, *J* = 8.8 Hz, 2H), 4.10 (s, 2H), 3.72 (s, 3H), 1.29 (s, 9H) ppm. ^13^C-NMR (DMSO-*d*_6_): δ 173.42, 172.38, 165.37, 164.21, 146.19, 137.40, 136.35, 129.42, 128.63, 125.24, 120.23, 112.39, 75.74, 52.41, 34.06, 32.25, 31.18 ppm. HRMS (EI) calc. for C_24_H_23_N_3_O_4_S^+^ 449.1409; found 449.1410.

*Methyl (Z)-2-cyano-2-(3-(4-(naphthalen-2-ylcarbamoyl)phenyl)-4-oxothiazolidin-2-ylidene)acetate* (**38**). White solid, Yield: 48%. Mp 285.0–286.0 °C; ^1^H-NMR (DMSO-*d*_6_): δ 10.61 (s, 1H), 8.49 (d, *J* = 1.2 Hz, 1H), 8.16 (d, *J* = 8.4 Hz, 2H), 7.95–7.84 (m, 4H), 7.63 (t, *J* = 8.4 Hz, 2H), 7.51 (t, *J* = 7.4 Hz, 1H), 7.45 (t, *J* = 7.4 Hz, 1H), 4.11 (s, 2H), 3.73 (s, 3H) ppm. ^13^C-NMR (DMSO-_d6_): δ 173.41, 172.35, 165.38, 164.66, 137.55, 136.57, 136.27, 133.27, 130.07, 129.50, 128.73, 128.18, 127.44, 126.40, 124.88, 120.96, 116.78, 112.41, 75.81, 52.41, 32.25 ppm. HRMS (EI) calc. for C_24_H_17_N_3_O_4_S^+^ 443.0940; found 443.0938.

*Methyl (Z)-2-(3-(4-(benzylcarbamoyl)phenyl)-4-oxothiazolidin-2-ylidene)-2-cyanoacetate* (**39**). White solid, Yield: 68%. Mp 117.3–118.1 °C; ^1^H-NMR (DMSO-*d*_6_): δ 9.22 (t, *J* = 5.6 Hz, 1H), 8.03 (d, *J* = 8.4 Hz, 2H), 7.54 (d, *J* = 8.4 Hz, 2H), 7.37–7.32 (m, 4H), 7.27–7.22 (m, 1H), 4.51 (d, *J* = 5.6 Hz, 2H), 4.08 (s, 2H), 3.71 (s, 3H) ppm. ^13^C-NMR (DMSO-*d*_6_): δ 173.39, 172.35, 165.37, 165.20, 139.46, 137.20, 135.82, 129.39, 128.28, 128.23, 127.27, 126.78, 112.33, 75.78, 52.39, 42.72, 32.22 ppm. HRMS (EI) calc. for C_21_H_17_N_3_O_4_S^+^ 407.0940; found 407.0939.

*Methyl (Z)-2-(3-(4-((5-acetylnaphthalen-2-yl)carbamoyl)phenyl)-4-oxothiazolidin-2-ylidene)-2-cyanoacetate* (**45**). White solid, Yield: 43%. Mp 275.0–275.8 °C; ^1^H-NMR (DMSO-*d*_6_): δ 10.73 (s, 1H), 9.10 (d, *J* = 1.6 Hz, 1H), 8.17 (d, *J* = 8.4 Hz, 2H), 8.12 (d, *J* = 7.2 Hz, 1H), 8.11–8.08 (m, 2H), 8.01 (d, *J* = 8.8 Hz, 1H), 7.62 (d, *J* = 8.4 Hz, 2H), 7.53 (t, *J* = 7.8 Hz, 1H), 4.11 (s, 2H), 3.73 (s, 3H), 2.74 (s, 3H) ppm. ^13^C-NMR (DMSO-*d*_6_): δ 201.53, 173.40, 172.34, 165.37, 164.72, 138.60, 137.59, 136.18, 134.45, 132.43, 130.57, 129.84, 129.60, 129.45, 128.85, 128.81, 123.62, 121.26, 115.06, 112.38, 75.82, 52.40, 32.25, 30.08 ppm. HRMS (EI) calc. for C_26_H_19_N_3_O_5_S^+^ 485.1045; found 485.1046.

#### 3.2.4. Synthesis of Compounds **42**, **43** and **46**

##### Synthesis of (Z)-2-((4-(2-(1-cyano-2-methoxy-2-oxoethylidene)-4-oxothiazolidin-3-yl)benzamido)methyl)-benzoic acid (**42**)

TCDI (2.4 mmol) was added to a solution of compound **IM** in THF (10 mL). Then the mixture was stirred at room temperature for 3 h. Then the mixture was added compound **42b** (2 mmol) and stirred at 50 °C for 2 h. The solvent was removed under reduced pressure after cooling to room temperature and the residue was purified by column chromatography (DCM/MeOH=20:3, V/V) to give **42** in a yield of 38%. White solid, Yield: 38%. Mp 217.3–218.1 °C; ^1^H-NMR (DMSO-*d*_6_): δ 13.08 (s, 1H), 9.14 (t, *J* = 5.6 Hz, 1H), 8.04 (d, *J* = 8.4 Hz, 2H), 7.90 (d, *J* = 7.6 Hz, 1H), 7.58–7.52 (m, 3H), 7.43 (d, *J* = 7.6 Hz, 1H), 7.37 (t, *J* = 7.6 Hz, 1H), 4.85 (d, *J* = 5.6 Hz, 2H), 4.09 (s, 2H), 3.71 (s, 3H) ppm. ^13^C-NMR (DMSO-*d*_6_): δ 173.93, 172.89, 168.93, 165.92, 165.87, 140.81, 137.76, 136.24, 132.51, 130.87, 129.94, 128.74, 127.76, 127.22, 112.86, 76.22, 52.90, 41.80, 32.73 ppm. HRMS (EI) calc. for C_22_H_17_N_3_O_6_S^+^ 451.0844; found 451.0838.

##### Synthesis of (Z)-3-((4-(2-(1-cyano-2-methoxy-2-oxoethylidene)-4-oxothiazolidin-3-yl)benzamido)methyl) benzoic acid (**43**)

CF_3_COOH (1.5 mL) was added to a solution of compound **43c** (1 mmol) in DCM (15 mL). Then the mixture was stirred at room temperature overnight. The solvent was removed under reduced pressure and the residue was crystallized from DCM/MeOH to give compound **43** in a yield of 85%.

White solid, Mp 205.7–206.6 °C; ^1^H-NMR (DMSO-*d*_6_): δ 12.92 (s, 1H), 9.26 (t, *J* = 5.6 Hz, 1H), 8.02 (d, *J* = 8.4 Hz, 2H), 7.94 (s, 1H), 7.84 (d, *J* = 8.0 Hz, 1H), 7.59 (d, *J* = 7.6 Hz, 1H), 7.54 (d, *J* = 8.4 Hz, 2H), 7.47 (t, *J* = 8.0 Hz, 1H), 4.56 (d, *J* = 5.6 Hz, 2H), 4.09 (s, 2H), 3.71 (s, 3H) ppm. ^13^C-NMR (DMSO-*d*_6_): δ 173.34, 172.29, 167.22, 165.34, 165.25, 139.96, 137.26, 135.68, 131.88, 130.84, 129.42, 128.57, 128.24, 128.21, 127.81, 112.30, 75.81, 52.37, 42.53, 32.19 ppm. HRMS (ESI) calc. for C_22_H_17_N_3_O_6_SNa^+^ (M + Na)^+^, 474.0736; found 474.0737.

##### Synthesis of 2-(((E)-(4-((Z)-2-(1-cyano-2-methoxy-2-oxoethylidene)-4-oxothiazolidin-3-yl)benzamido)- methylene)amino)benzoic acid (**46**)

A drop of concentrated HCl was added to a solution of compound **46b** (1 mmol) and 2-formylbenzoic acid in EtOH (30 mL). Then the mixture was stirred at room temperature until the reaction was completed. The solvent was removed under reduced pressure and the residue was crystallized from DCM/MeOH to give compound **46** in yield of 80%. White solid, Yield: 80%. Mp 222.8–223.5 °C; ^1^H-NMR (DMSO-*d*_6_): δ 13.26 (s, 1H), 12.25 (s, 1H), 9.24 (s, 1H), 8.12 – 8.05 (m, 3H), 7.92 (d, *J* = 7.6 Hz, 1H), 7.67 (t, *J* = 7.6 Hz, 1H), 7.60 (d, *J* = 8.4 Hz, 2H), 7.55 (t, *J* = 7.6 Hz, 1H), 4.10 (s, 2H), 3.72 (s, 3H) ppm. ^13^C-NMR (DMSO-*d*_6_): δ 173.39, 172.33, 168.04, 165.36, 162.28, 147.08, 137.63, 134.78, 134.53, 132.00, 130.67, 130.30, 129.69, 129.51, 128.72, 126.68, 112.35, 75.80, 52.40, 32.24 ppm. HRMS (ESI) calc. for C_22_H_16_N_4_O_6_SNa^+^ (M + Na)^+^, 487.0688; found 487.0689.

### 3.3. In Vitro Assays

The plasmid pET-19b–*h*DHODH (Met30-Arg396) was transformed into BL21 (DE3) *E. coli* cells for protein production. Cells were grown at 37 °C in a rich medium, and were induced with 1 mM isopropyl-β-D-thiogalactoside (IPTG) at an OD_600_ of 0.6–0.8. After incubation for an additional 18 h at 16 °C, the cells were harvested by centrifugation. The harvested cells were suspended in buffer A (50 mM HEPES pH 8.0, 400 mM NaCl, 10% glycerol) and disrupted by a high-pressure cracker at 4 °C. Triton X-100 was added to a final concentration of 1% into the lysate before centrifugation. The supernatant was loaded onto a HiTrap Chelating column (5 mL; GE Healthcare, Uppsala, Sweden). Pure *h*DHODH was eluted with buffer A, 0.1% Triton X-100 and 160 mM imidazole.

The purified *h*DHODH was diluted into a final concentration of 10 nM with the assay buffer contained 50 mM HEPES pH 8.0, 150 mM KCl, 0.1% Triton X-100. UQ_0_ and DCIP were added to the assay buffer to final concentrations of 100 and 120 μM, respectively. The dihydroorotate was added to a final concentration of 500 μM to initiate the reaction. Brequinar was measured as the positive control. Inhibition rate was calculated from (1-*V_i_*/*V_0_*) × 100. For the determination of the IC_50_ values, eight to nine different concentrations were applied. Each inhibitor concentration point was tested in triplicate. IC50 values were calculated using the sigmoidal fitting option of the program Origin 8.0.

## 4. Conclusions

In conclusion, based on our previous work, three series of 4-thiazolidinone derivatives including biphenyl, diphenyl ether and amide groups were designed and synthesized as *h*DHODH inhibitors. The preliminary structure–activity relationships were investigated. The *h*DHODH inhibitory activities of several newly synthesized compounds and compounds **7** and **8** are at the same level, especially compounds **9** and **37** with IC_50_ values of 1.32 and 1.45 μM, respectively. Further modifications will be investigated to improve the activity of 4-thiazolidinone derivatives.

## Figures and Tables

**Table 1:**
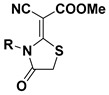
Structures and activities for 4-thiazolidinone analogs **9**–**18**.

Compd	R	% Inhibition at 10 µM	*h*DHODH IC_50_*^a^* (μM)
**7**	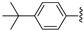	79.1	1.75
**8**	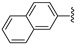	80.3	1.12
**9**	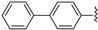	73.8	1.32
**10**	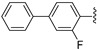	58.8	3.52
**11**	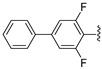	33.9	>10
**12**	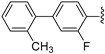	57.6	6.04
**13**	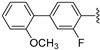	54.7	6.64
**14**	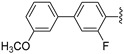	63.3	5.42
**15**	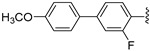	55.4	6.86
**16**	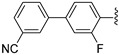	50.2	8.39
**17**	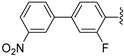	50.7	4.35
**18**	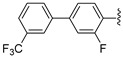	53.1	9.29
Brequinar			0.0084

*^a^* IC_50_ values were determined from three independent tests, and attempts to determine IC_50_ values were made if the inhibition rate at 10 μM was greater than 50%.

**Table 2:**
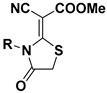
Structures and activities for 4-thiazolidinone analogs **19**–**46.**

Compd	R	% Inhibition at 10 µM	*h*DHODH IC_50_ (μM)
**19**	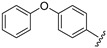	52.0	9.43
**20**	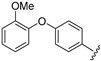	44.7	>10
**21**	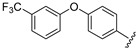	29.7	>10
**22**	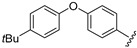	18.1	>10
**23**	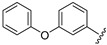	34.4	>10
**24**	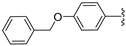	55.4	4.32
**25**	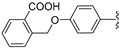	29.9	>10
**26**	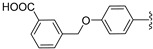	36.2	>10
**27**	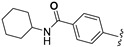	41.7	>10
**28**	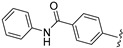	69.0	2.98
**29**	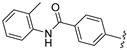	47.0	>10
**30**	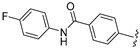	71.3	3.01
**31**	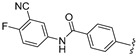	53.6	8.56
**32**	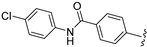	52.7	7.50
**33**	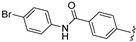	42.0	>10
**34**	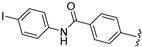	38.1	>10
**35**	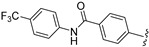	24.5	>10
**36**	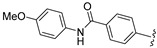	55.4	5.01
**37**	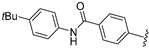	75.6	1.45
**38**	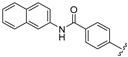	38.2	>10
**39**	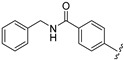	39.9	>10
**40**	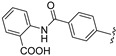	34.5	>10
**41**	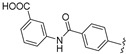	23.5	>10
**42**	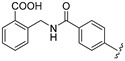	36.8	>10
**43**	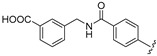	44.0	>10
**44**	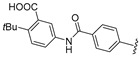	30.3	>10
**45**	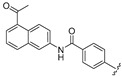	42.2	>10
**46**	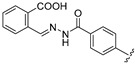	34.8	>10

## Supplementary Materials

Spectrums for target compounds **7**–**46** could be accessed in supplementary materials.
