# Supplementary material for: Structural Optimization and Structure–Activity Relationship of 4-Thiazolidinone Derivatives as Novel Inhibitors of Human Dihydroorotate Dehydrogenase
Source: Molecules. 2019 Jul 31;24(15):2780. doi: 10.3390/molecules24152780 (PMC6696179; doi:10.3390/molecules24152780)
Supplement: Supplementary file 1 [file molecules-24-02780-s001.pdf]

Supplementary materials for

Structural optimization and structure–activity relationship of 4-thiazolidinone derivatives as novel inhibitors of human dihydroorotate dehydrogenase

Fanxun Zeng [1,§], Lina Quan [2,§], Guantian Yang [1], Tiantian Qi [2], Letian Zhang [1], Shiliang Li [2], Honglin Li [2], Lili Zhu [2,*], Xiaoyong Xu [1,*]

[1] Shanghai Key Laboratory of Chemical Biology, School of Pharmacy, East China University of Science and Technology, 130 Meilong Road, Shanghai 200237, PR China; 429646704@qq.com (F.Z.); 853565296@qq.com (G.Y.); 125702429@qq.com (L.Z); xyxu@ecust.edu.cn (X.X.)

[2] Shanghai Key Laboratory of New Drug Design, State Key Laboratory of Bioreactor Engineering, School of Pharmacy, East China University of Science & Technology, Shanghai 200237, PR China; Inquan1991@163.com (L.Q.); 785823121@qq.com (T.Q.); Slli403@163.com (S.L.); hlli@ecust.edu.cn (H.L.); zhulfl@ecust.edu.cn (L.Z.)

* Correspondence:

1. Shanghai Key Laboratory of Chemical Biology, School of Pharmacy, East China University of Science and Technology, 130 Meilong Road, Shanghai 200237, PR China, xyxu@ecust.edu.cn (X.X.); Tel.: +86-21-64252945 (X.X);

2. Shanghai Key Laboratory of New Drug Design, State Key Laboratory of Bioreactor Engineering, School of Pharmacy, East China University of Science & Technology, Shanghai 200237, PR China zhulfl@ecust.edu.cn (L.Z.); Tel.: +86-21-64253379 (L.Z.)

§ These authors contributed equally to this work.

The copies of spectrum for target compounds 7-46.(^{1}H NMR, ^{19}F NMR and ^{13}C NMR spectra were recorded on Bruker AM-400 (^{1}H at 400 MHz, ^{13}C at 100 MHz, ^{19}F at 376 MHz) spectrometer with $CDCl_3$ or DMSO-d_6 as the solvent and TMS as the internal standard.**)**

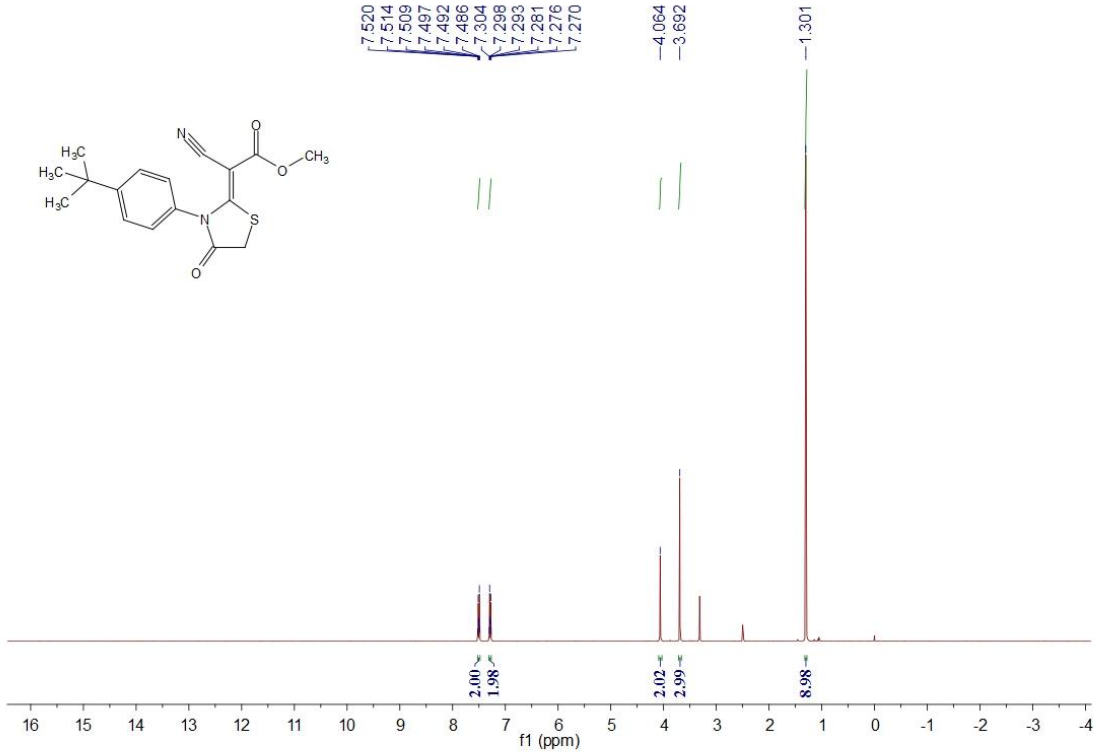

FigureS1.^{1}H-NMR of compound **7.**

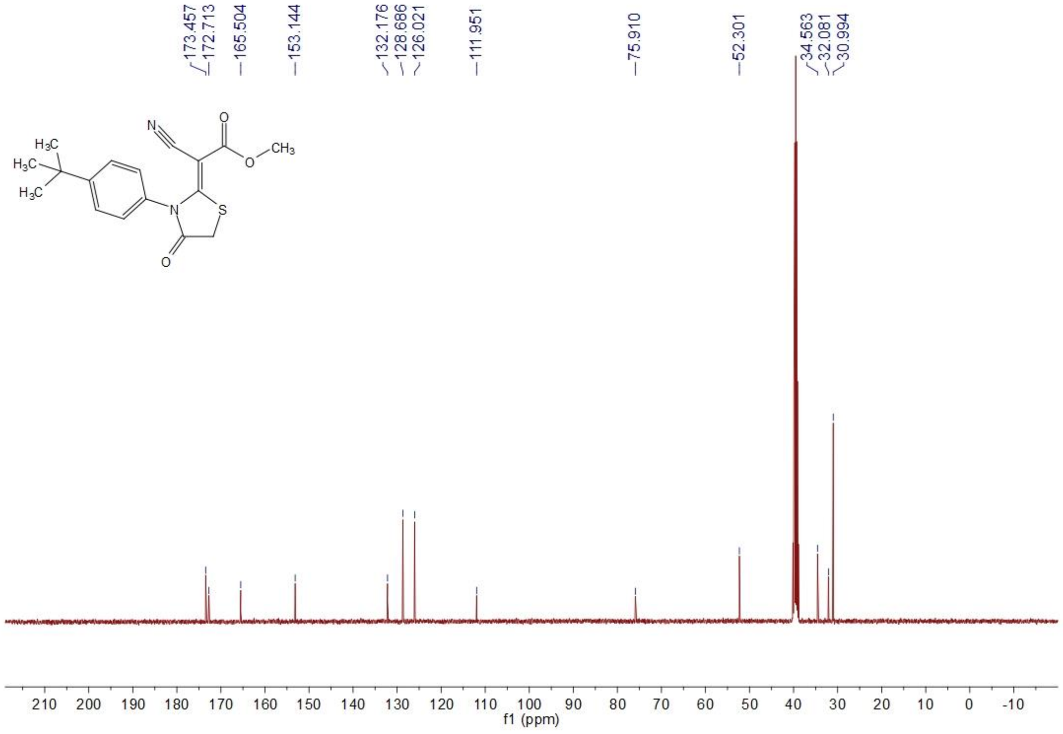

FigureS2.^{13}C-NMR of compound **7.**

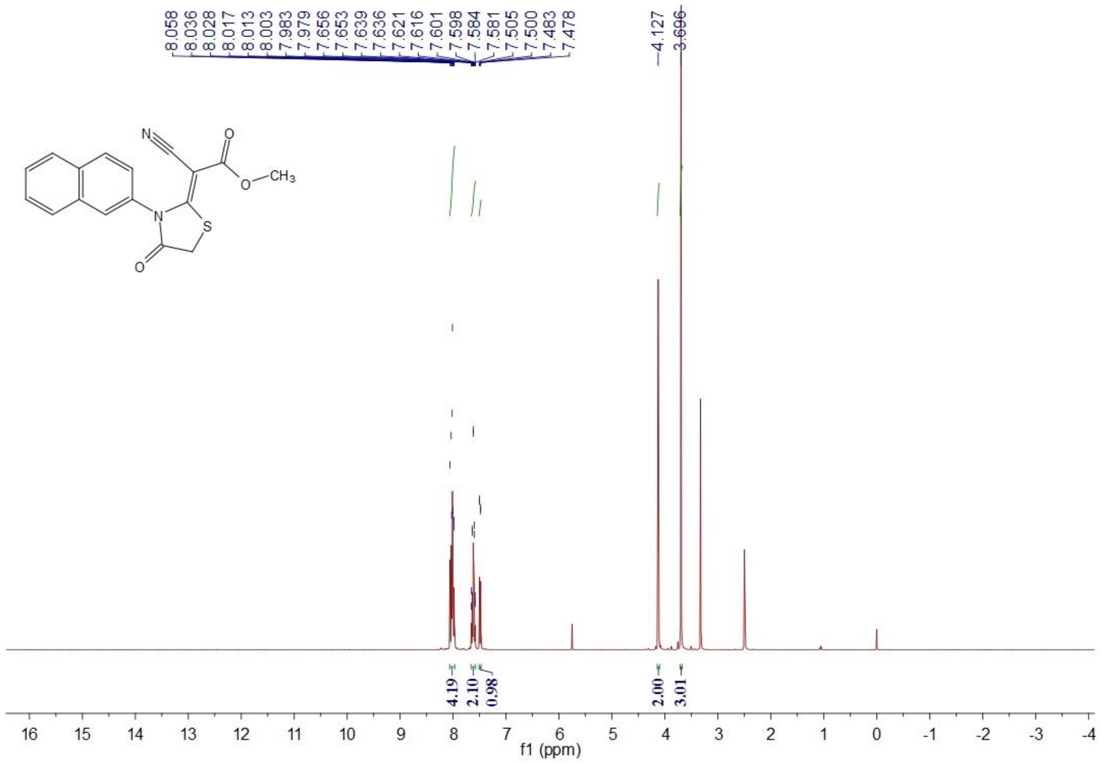

FigureS3.^{1}H-NMR of compound **8.**

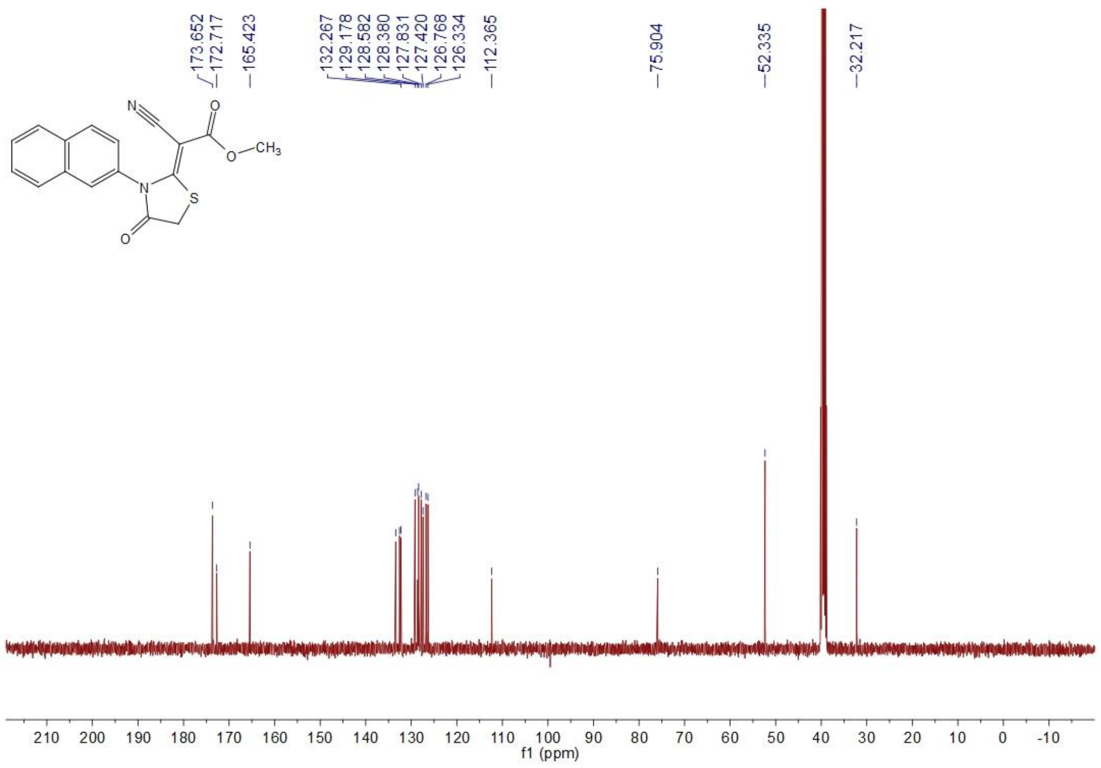

FigureS4.^{13}C-NMR of compound **8.**

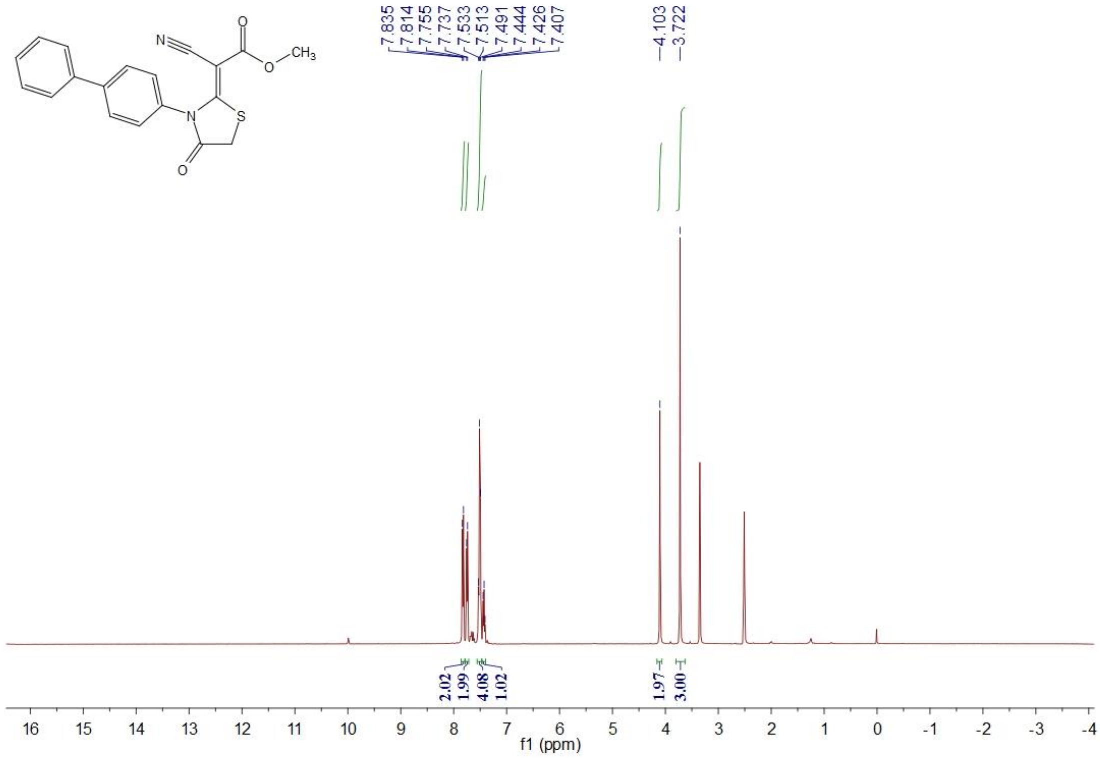

FigureS5.^{1}H-NMR of compound **9.**

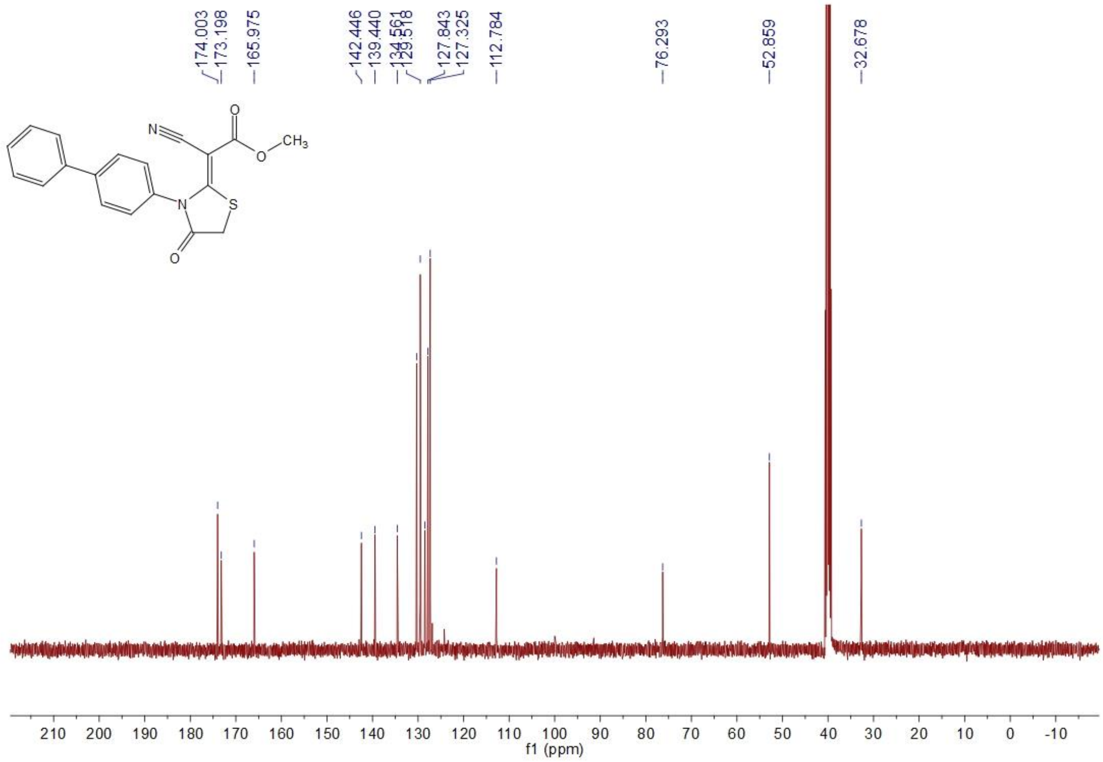

FigureS6.^{13}C-NMR of compound **9.**

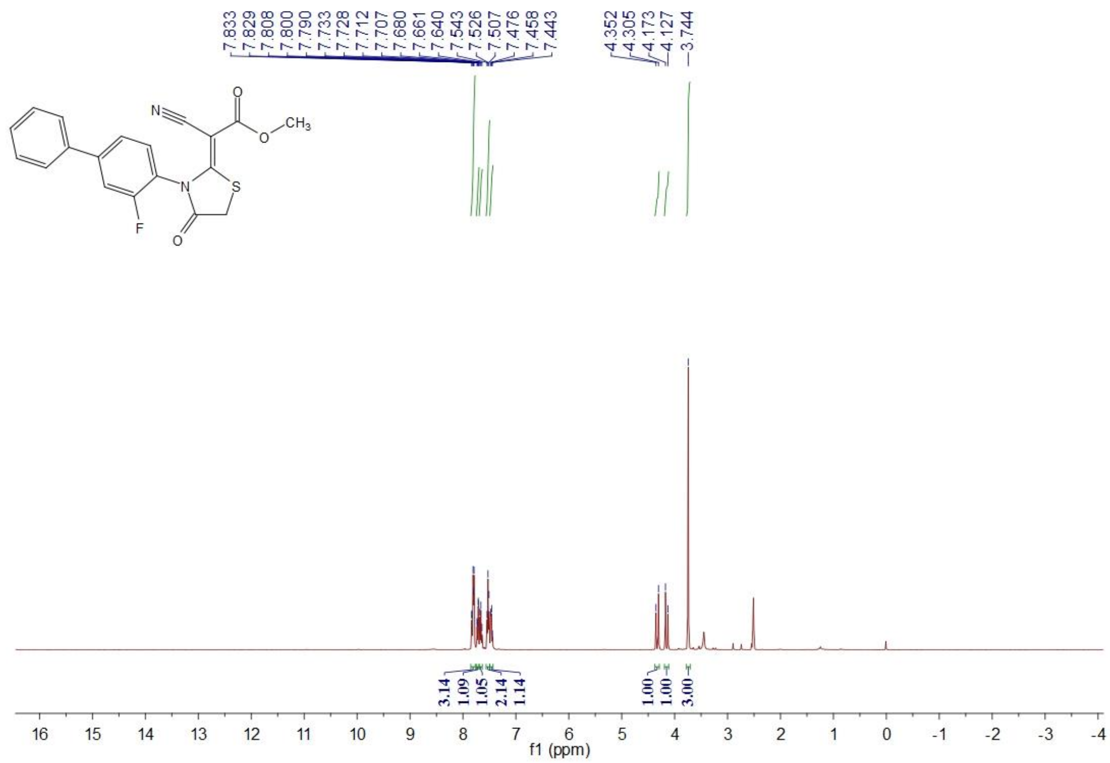

FigureS7.^{1}H-NMR of compound **10.**

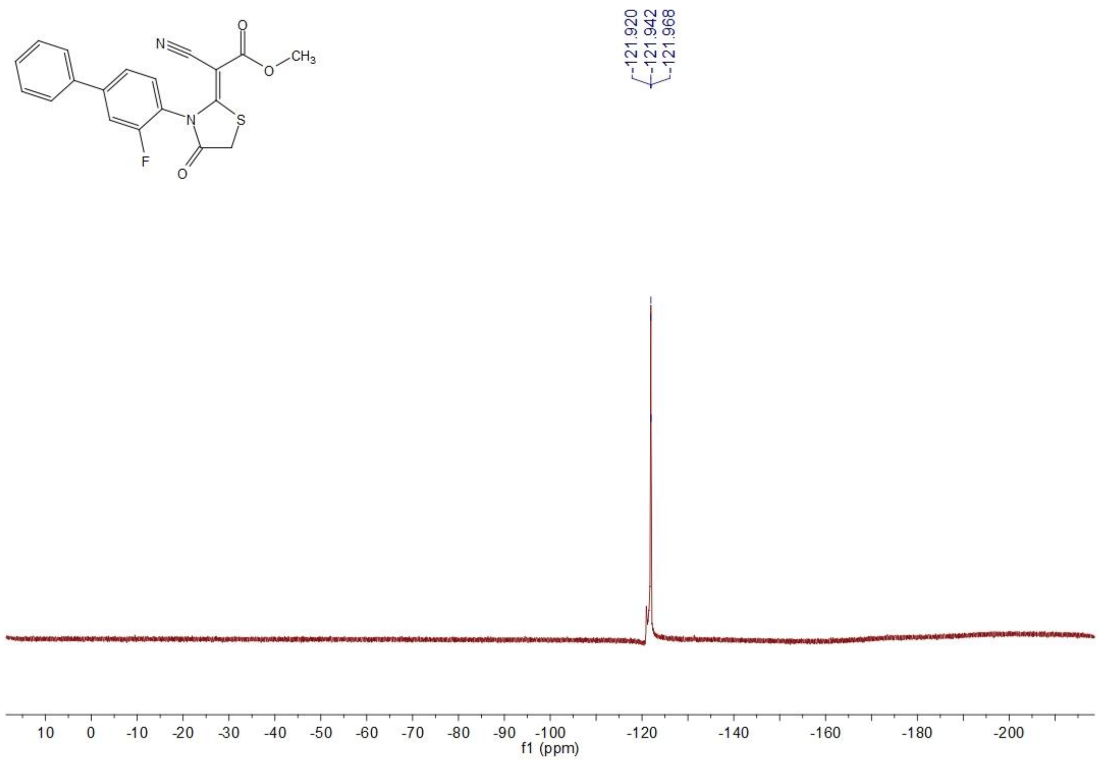

FigureS8.^{19}F-NMR of compound **10.**

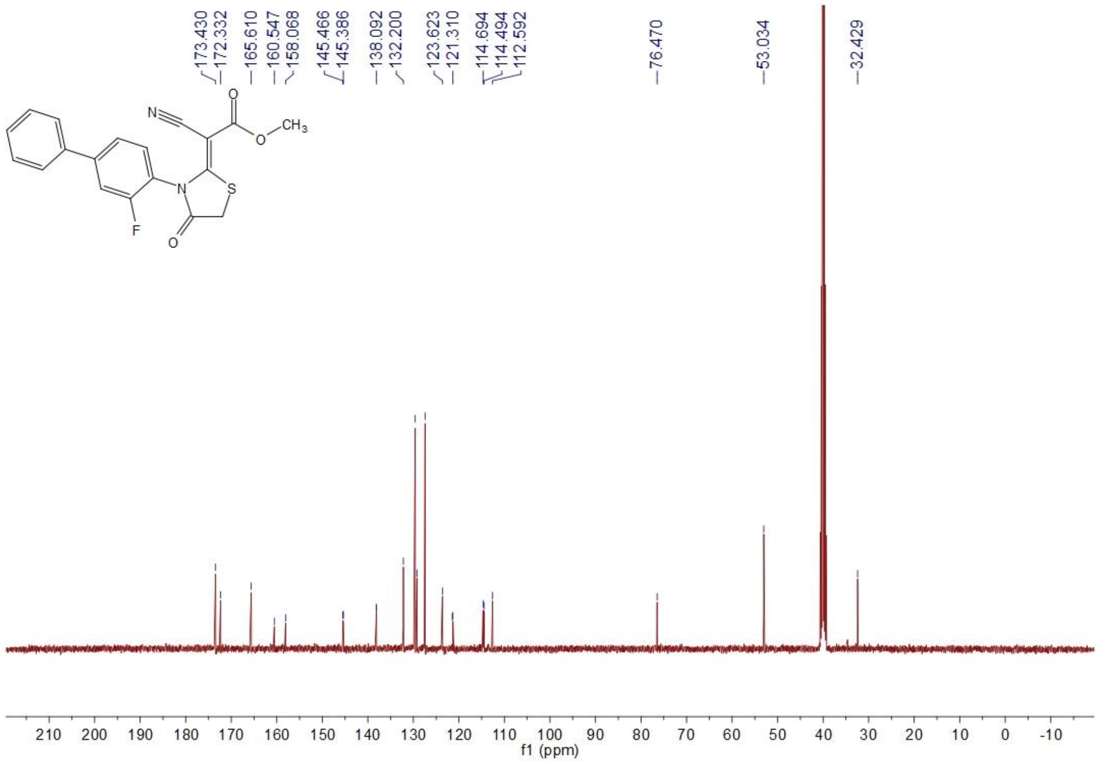

FigureS9.^{13}C-NMR of compound **10.**

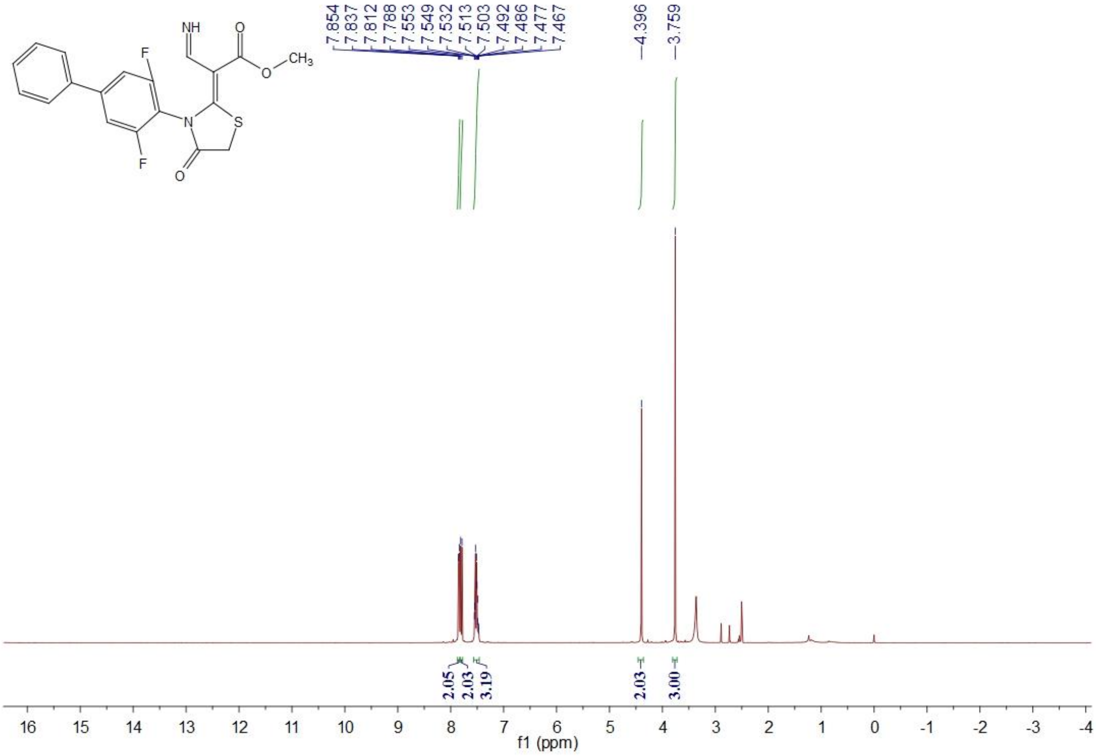

FigureS10.^{1}H-NMR of compound **11.**

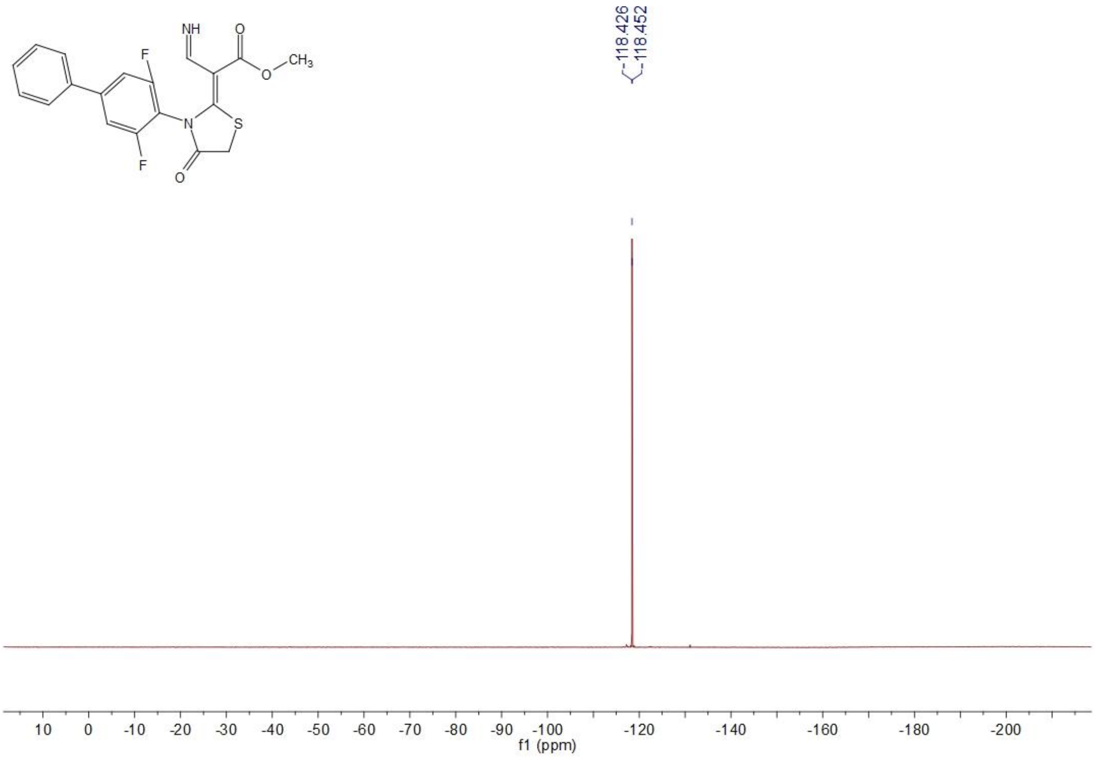

FigureS11.^{19}F-NMR of compound **11.**

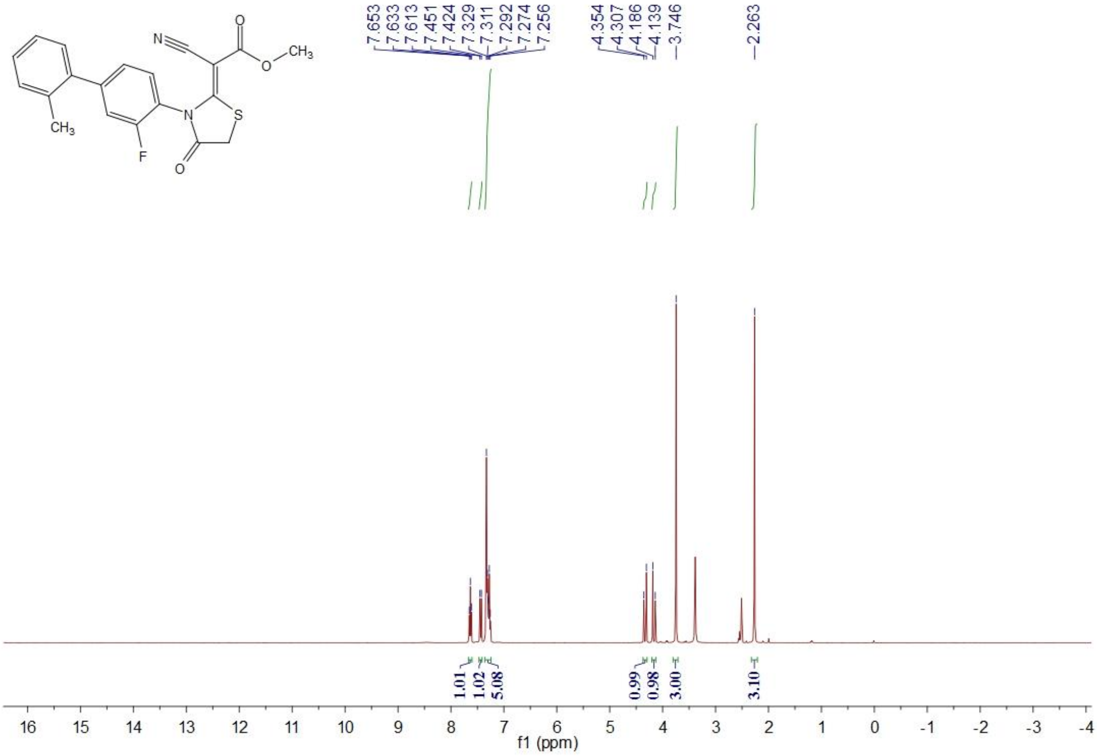

FigureS12.^{1}H-NMR of compound **12.**

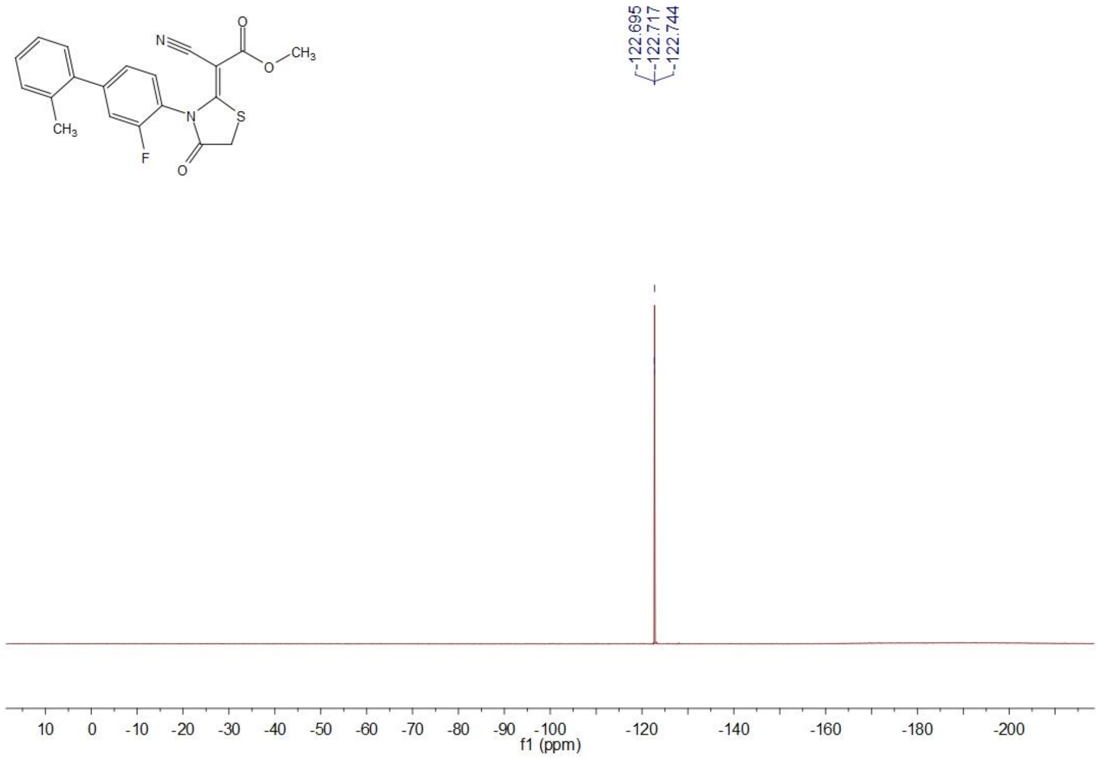

FigureS13.^{19}F-NMR of compound **12.**

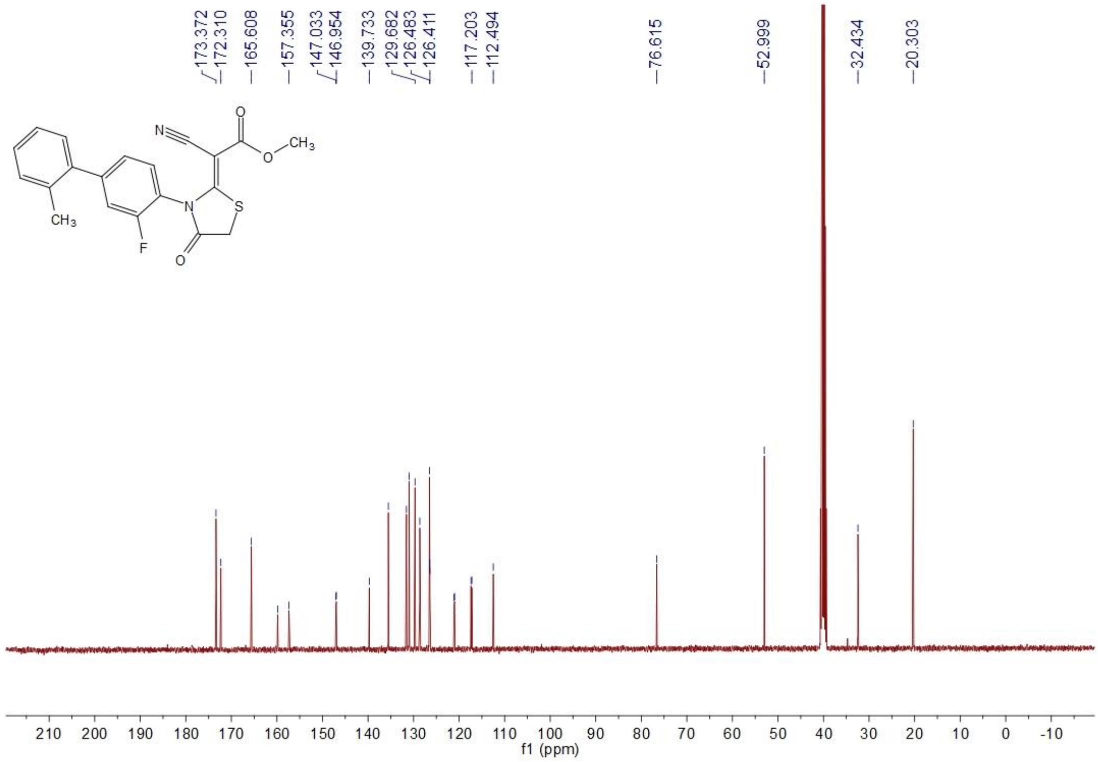

FigureS14.^{13}C-NMR of compound **12.**

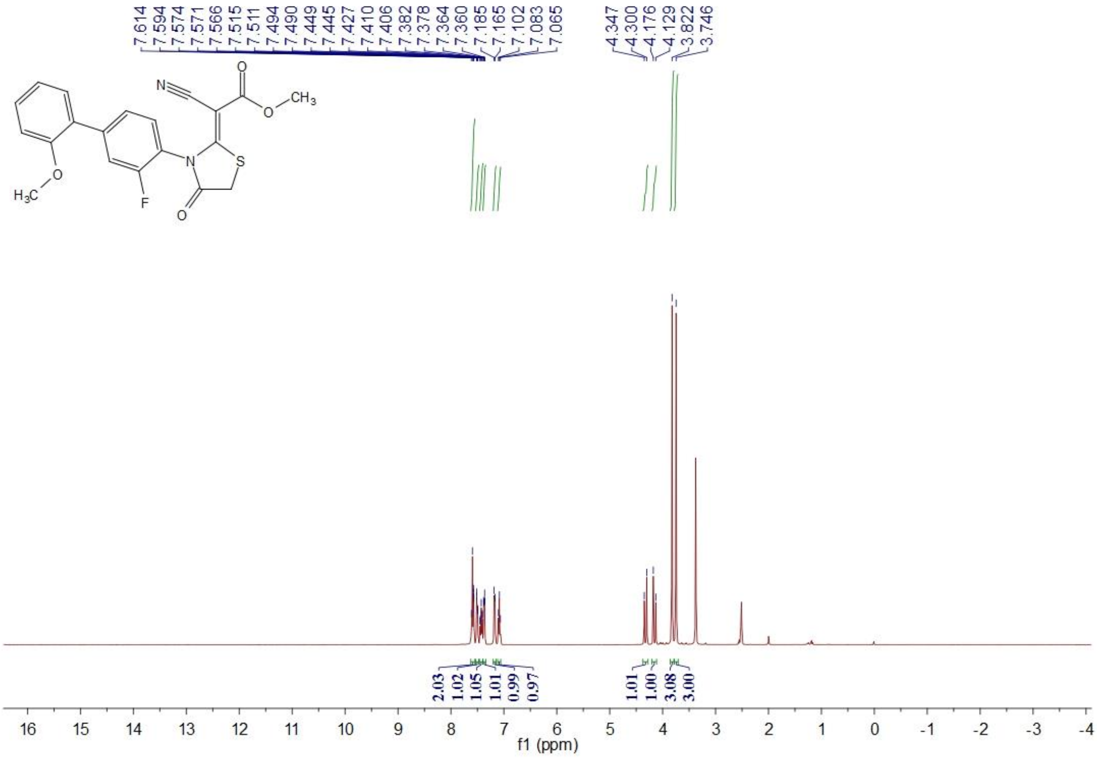

FigureS15.^{1}H-NMR of compound **13.**

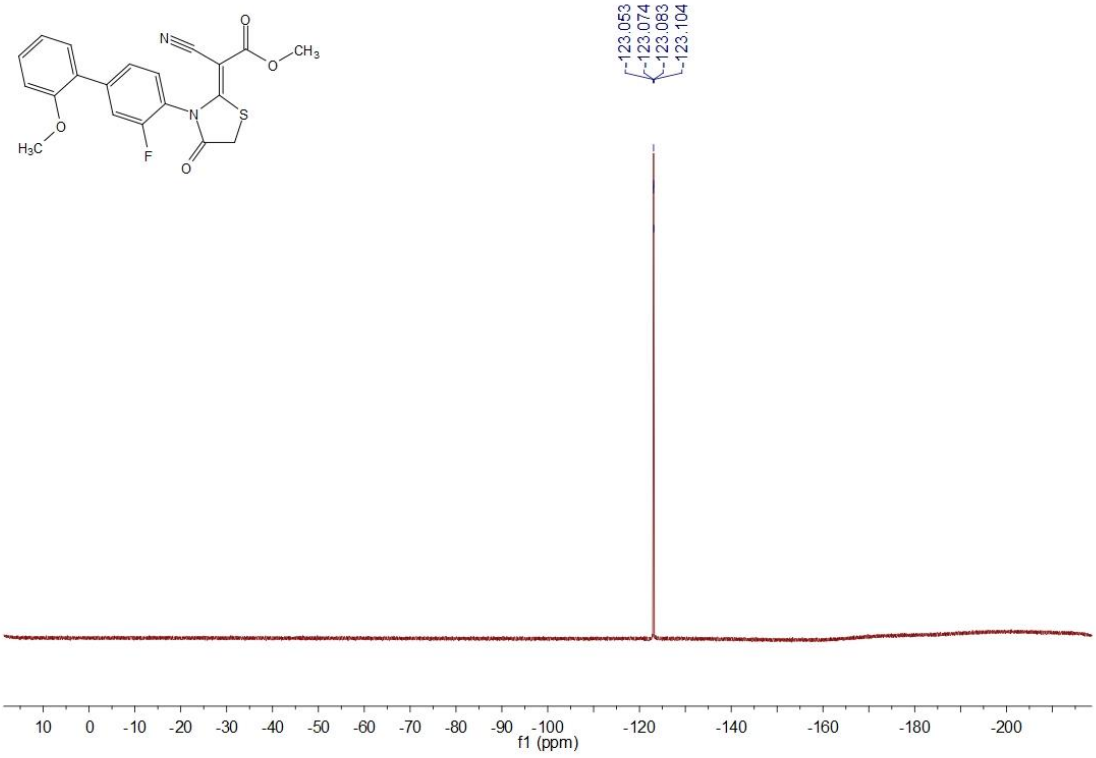

FigureS16.^{19}F-NMR of compound **13.**

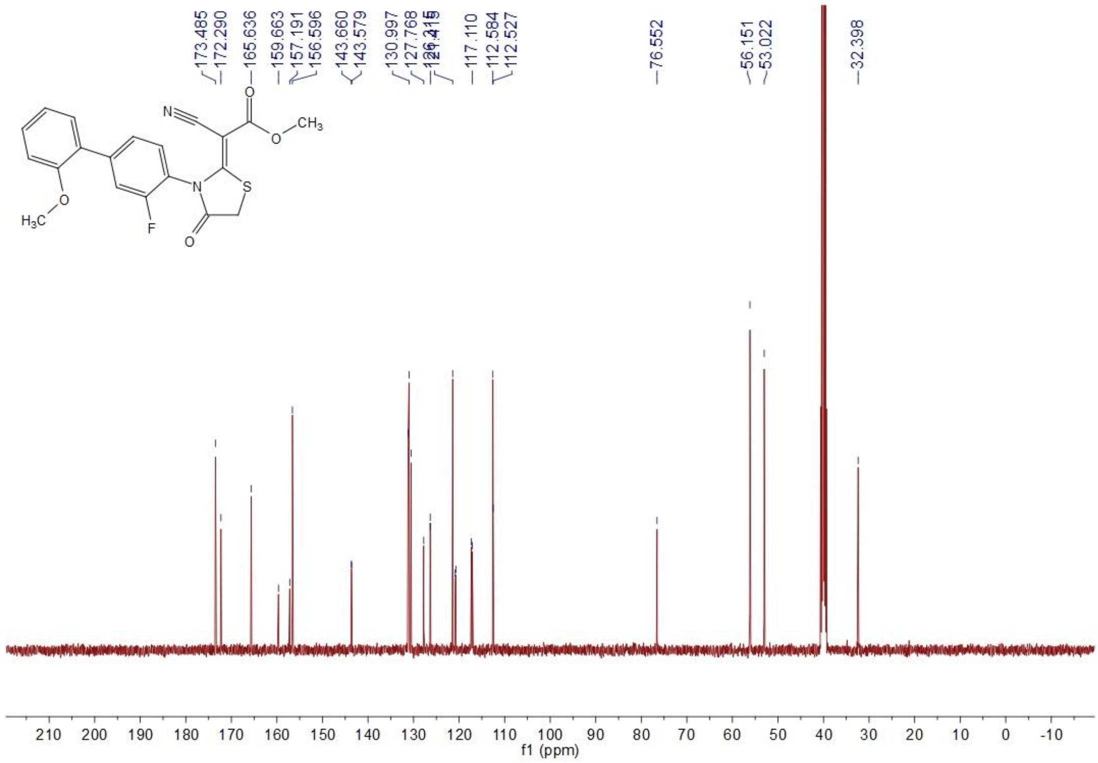

FigureS17.^{13}C-NMR of compound **13.**

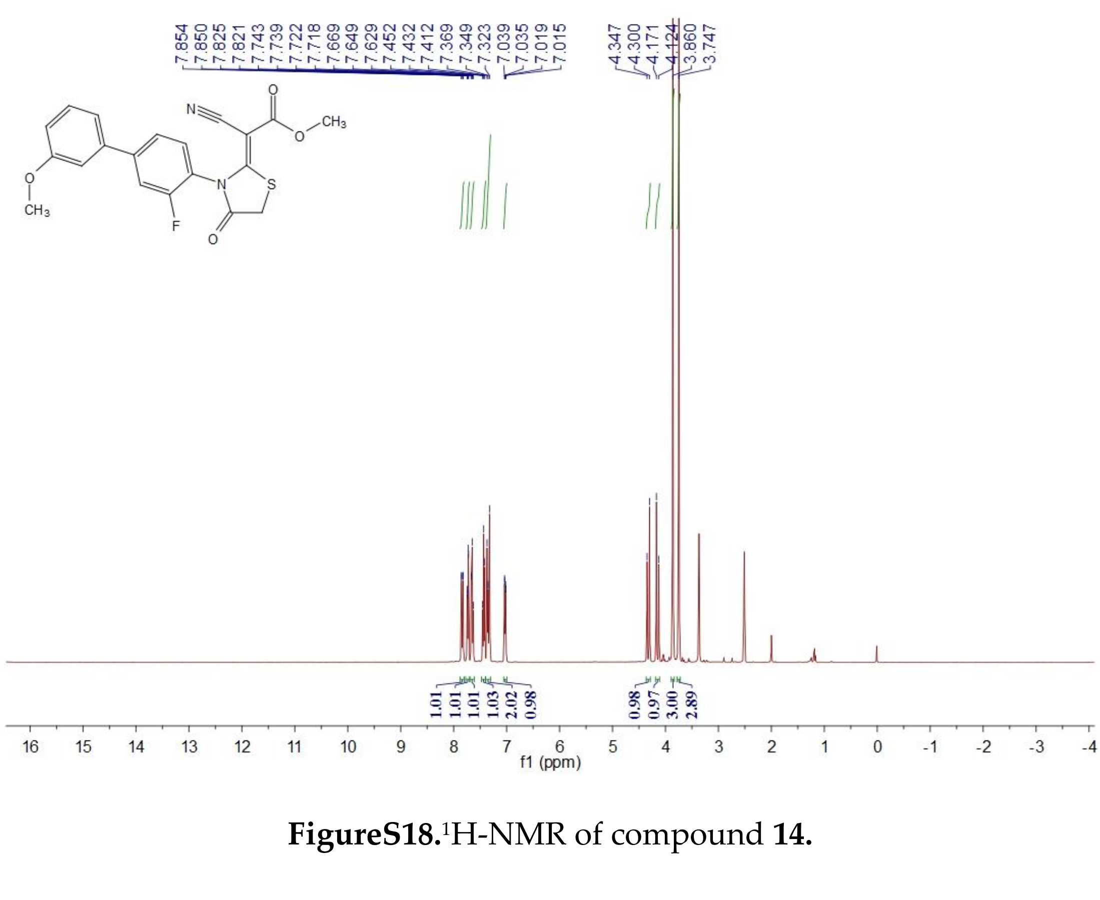

FigureS18.^{1}H-NMR of compound **14.**

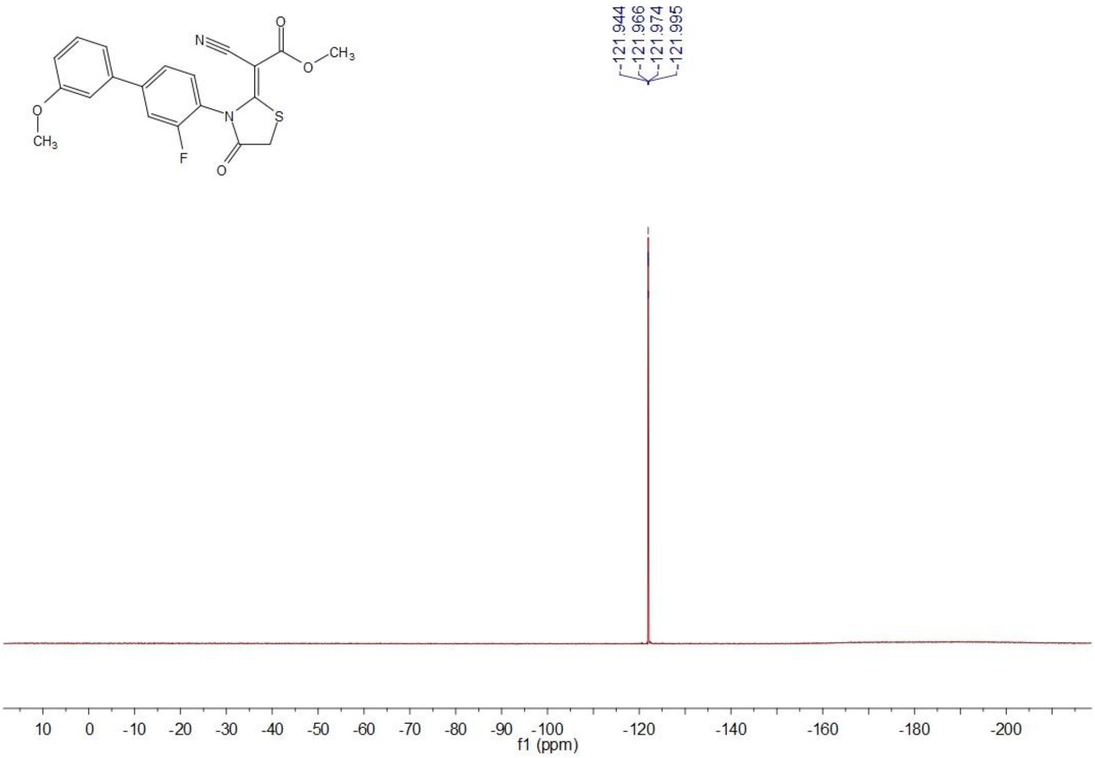

FigureS19.^{19}F-NMR of compound **14.**

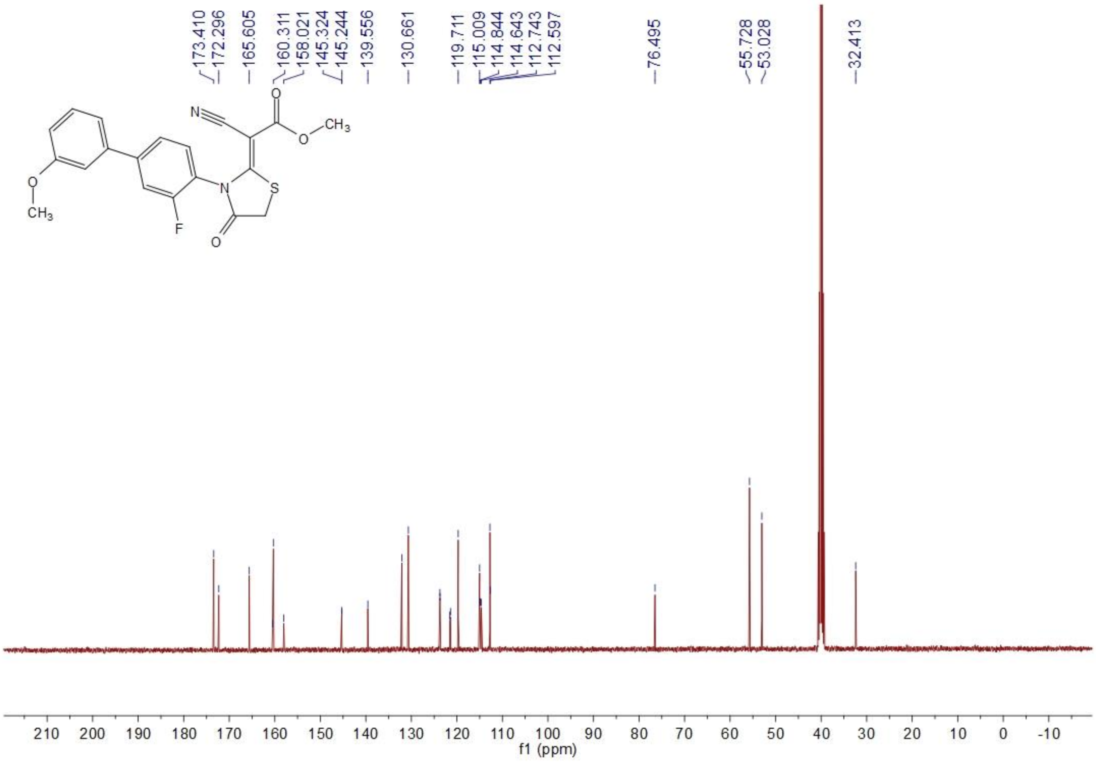

FigureS20.^{13}C-NMR of compound **14.**

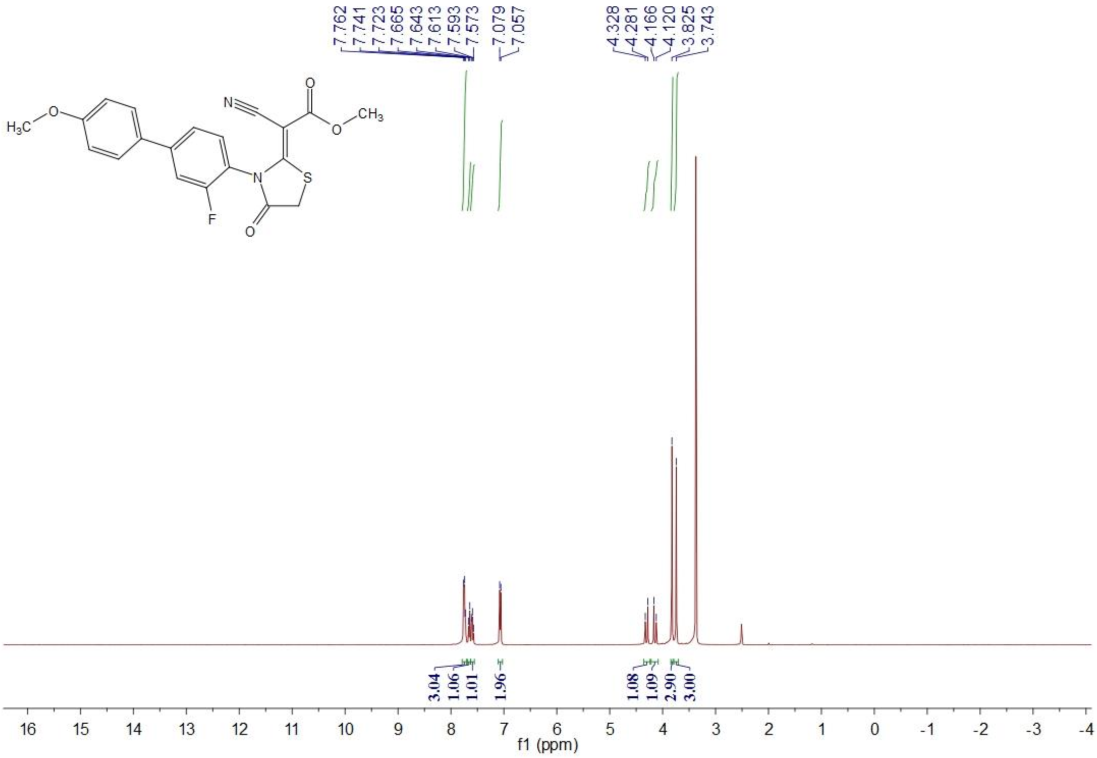

FigureS21.^{1}H-NMR of compound **15**

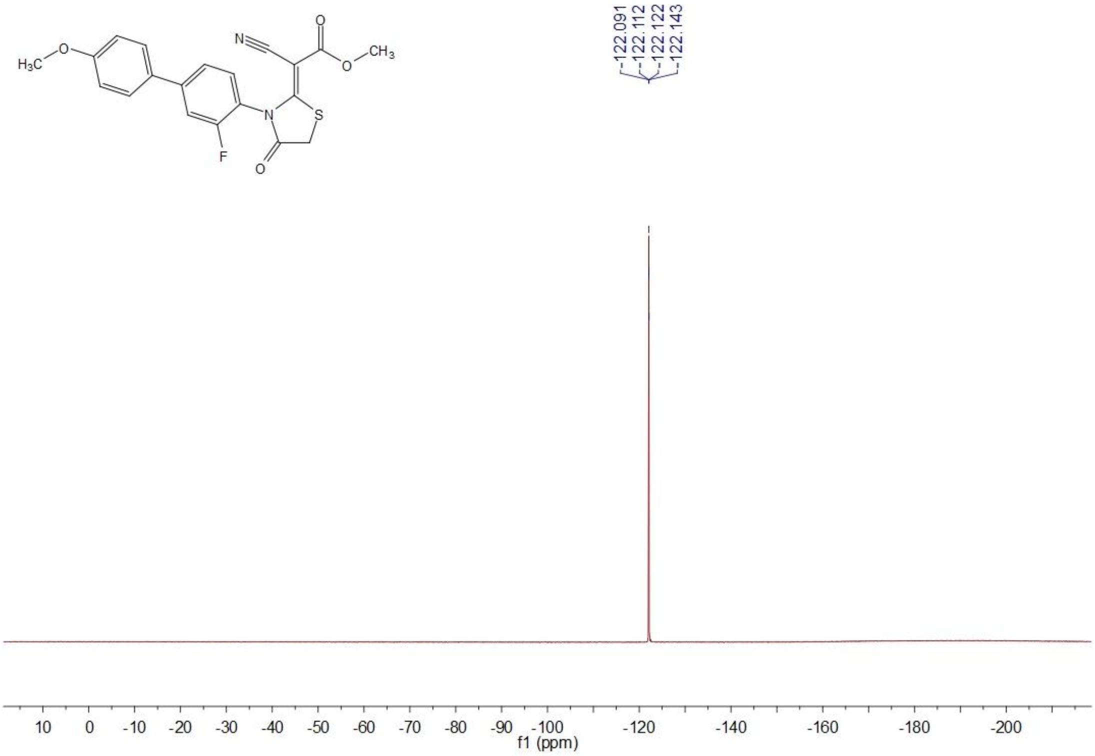

.

FigureS22.^{19}F-NMR of compound **15.**

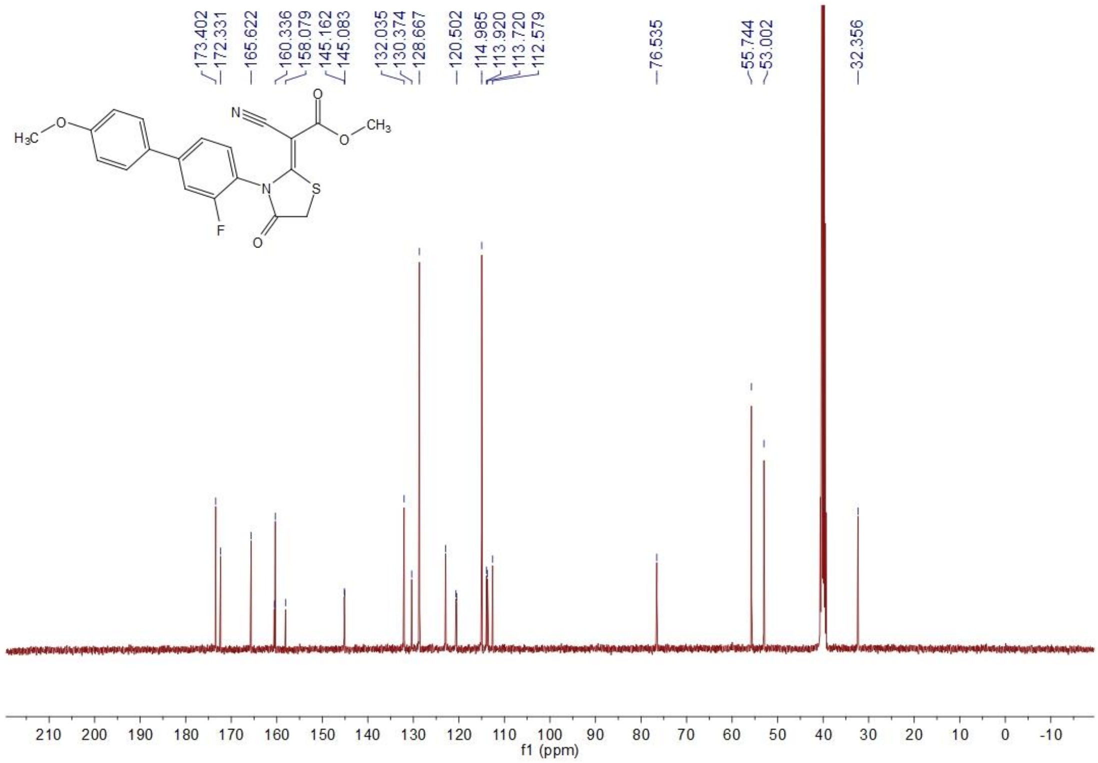

FigureS23.^{13}C-NMR of compound **15.**

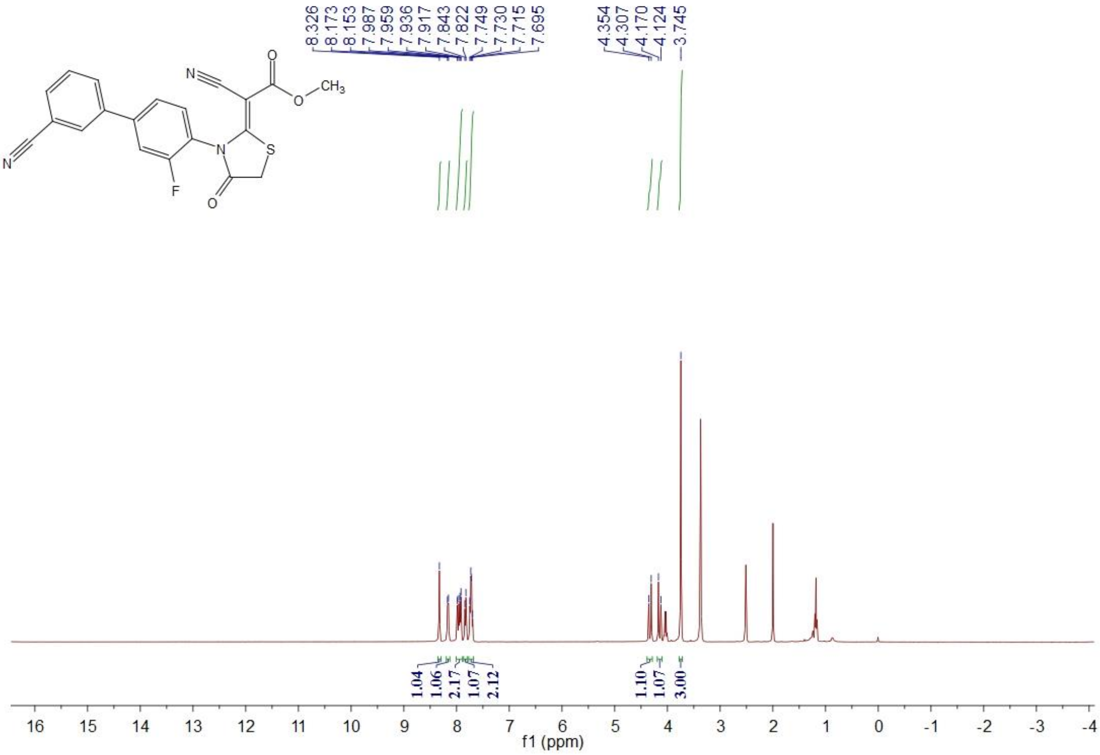

FigureS24.^{1}H-NMR of compound **16.**

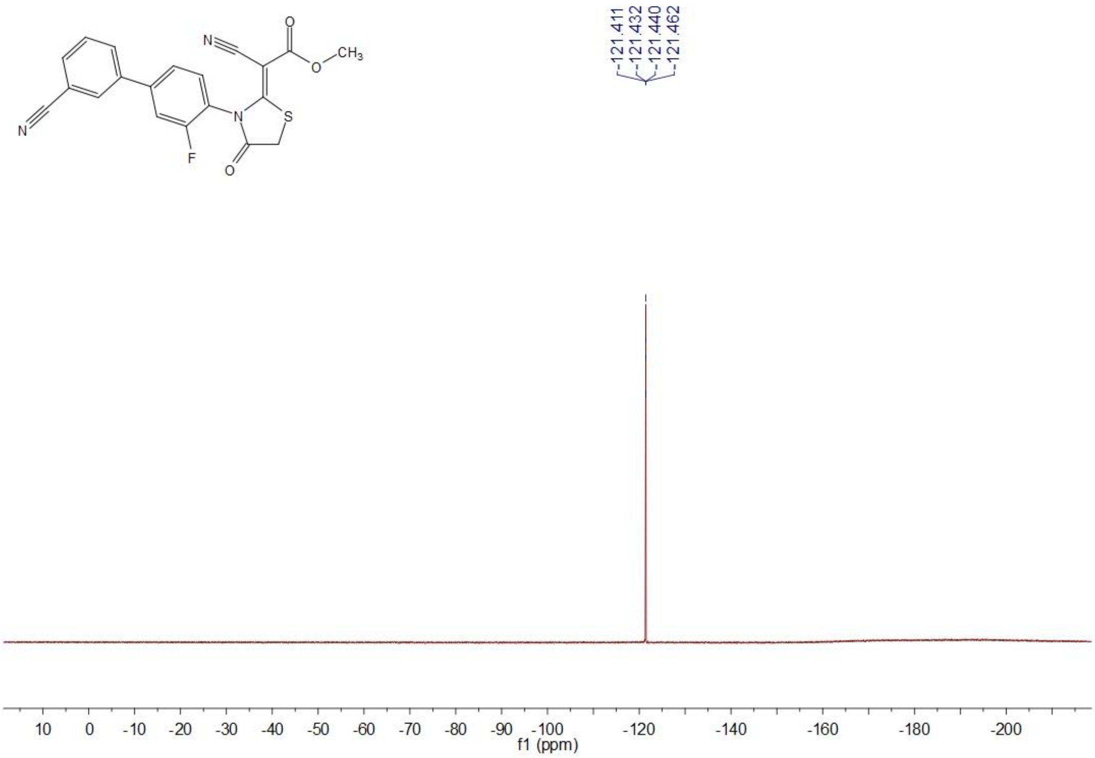

FigureS25.^{19}F-NMR of compound **16.**

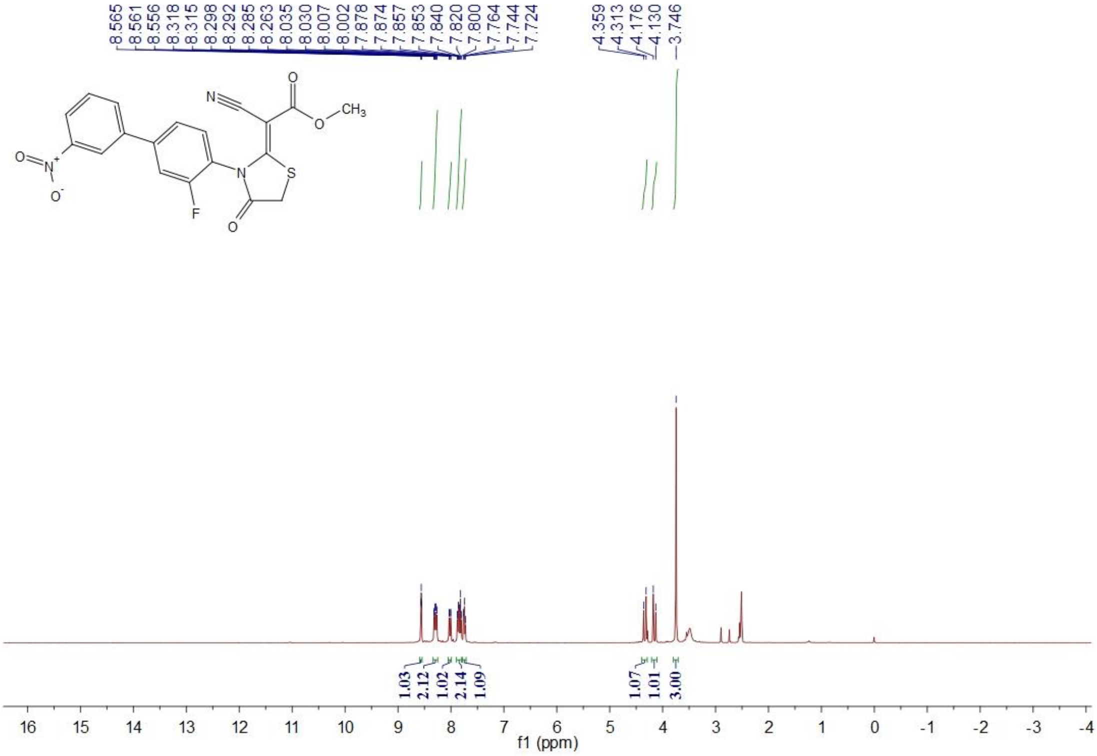

FigureS26.^{1}H-NMR of compound **17.**

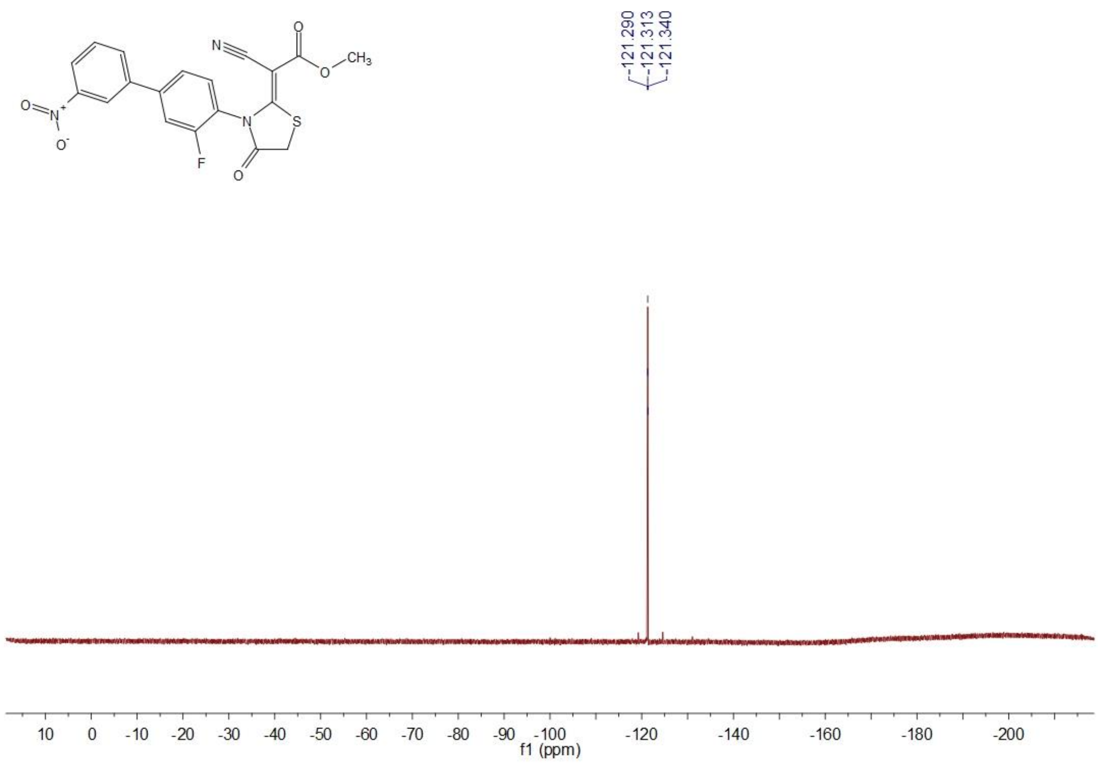

FigureS27.^{19}F-NMR of compound **17.**

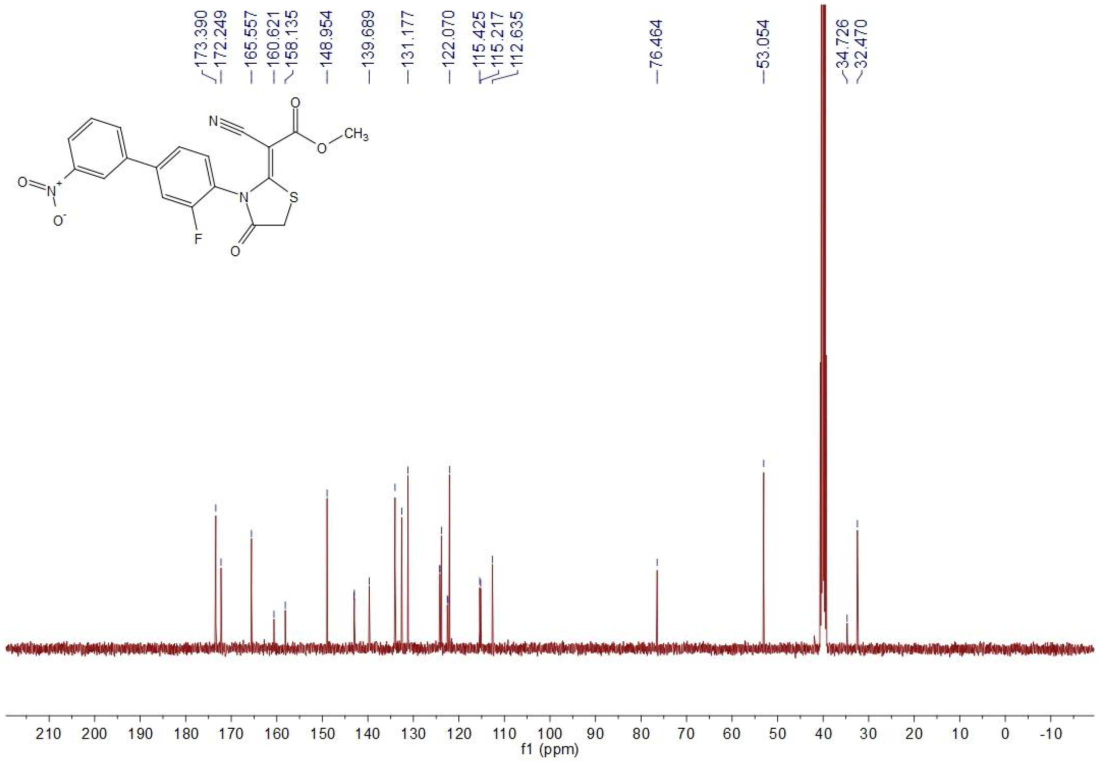

FigureS28. ^{13}C-NMR of compound **17.**

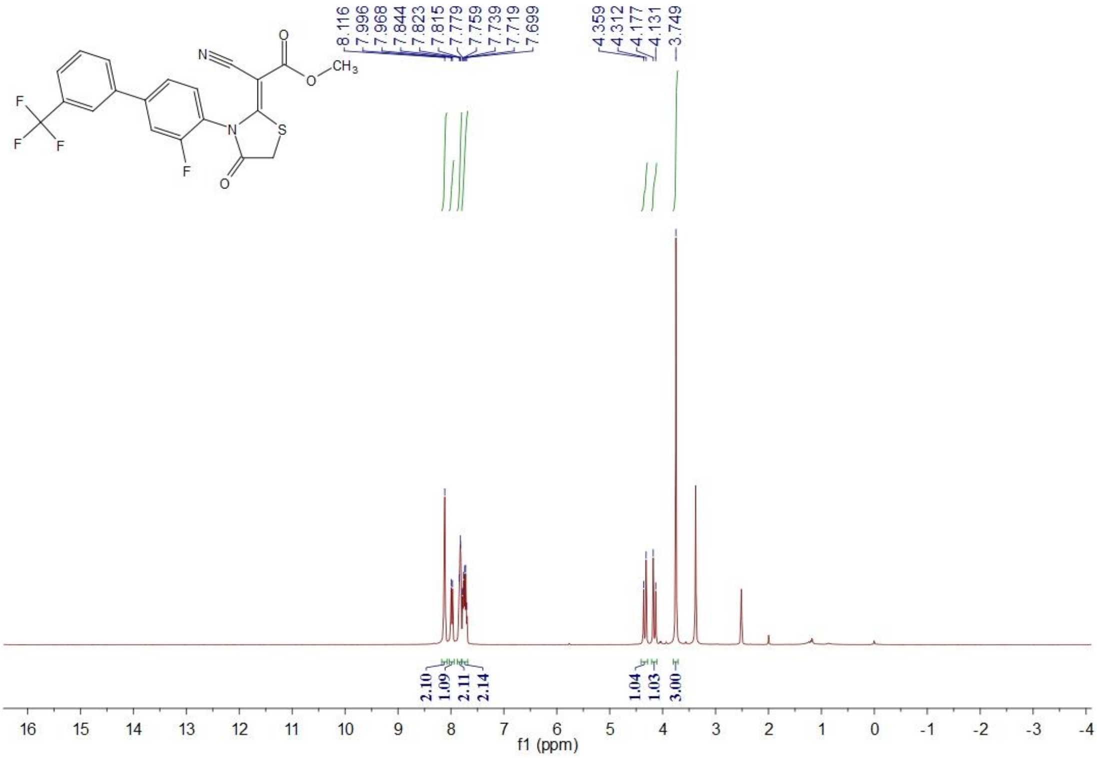

FigureS29.^{1}H-NMR of compound **18.**

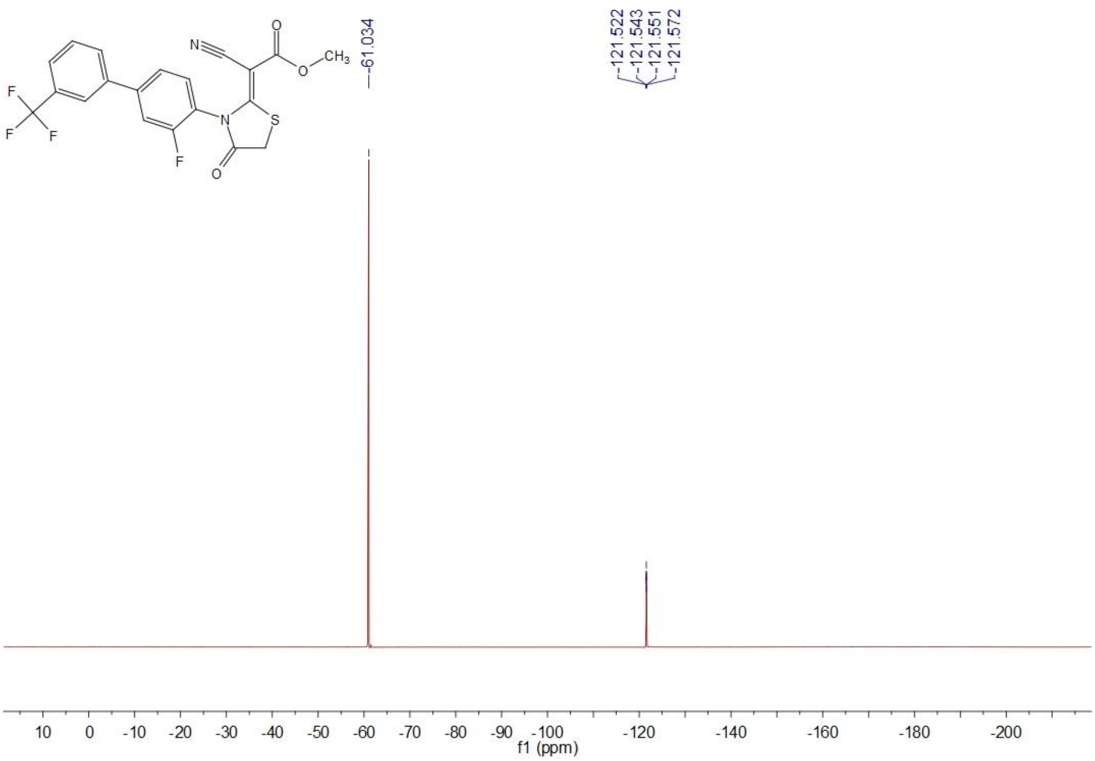

FigureS30.^{19}F-NMR of compound **18.**

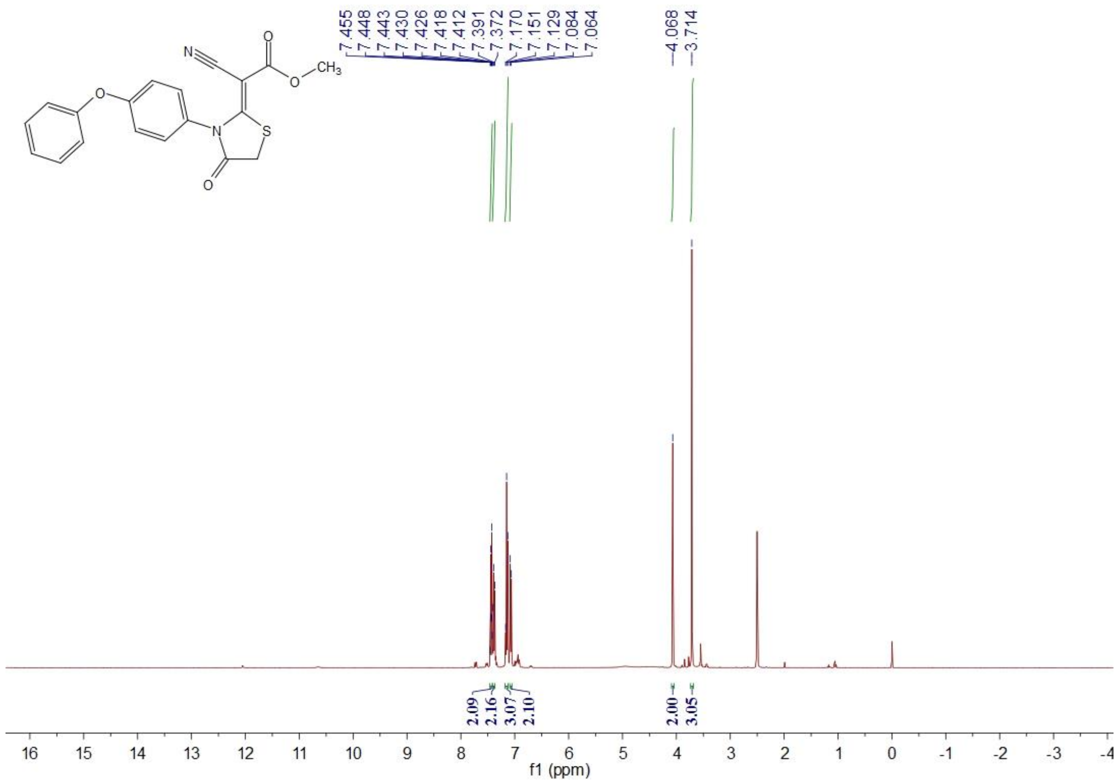

FigureS31.^{1}H-NMR of compound **19.**

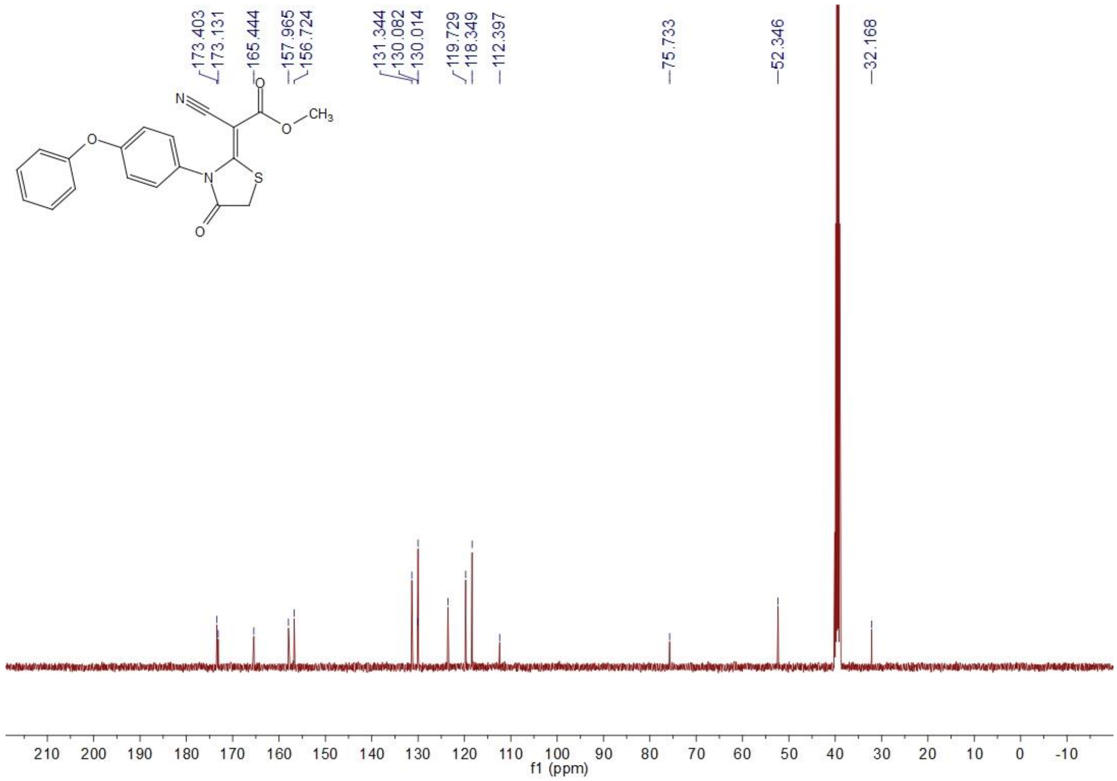

FigureS32.^{13}C-NMR of compound **19.**

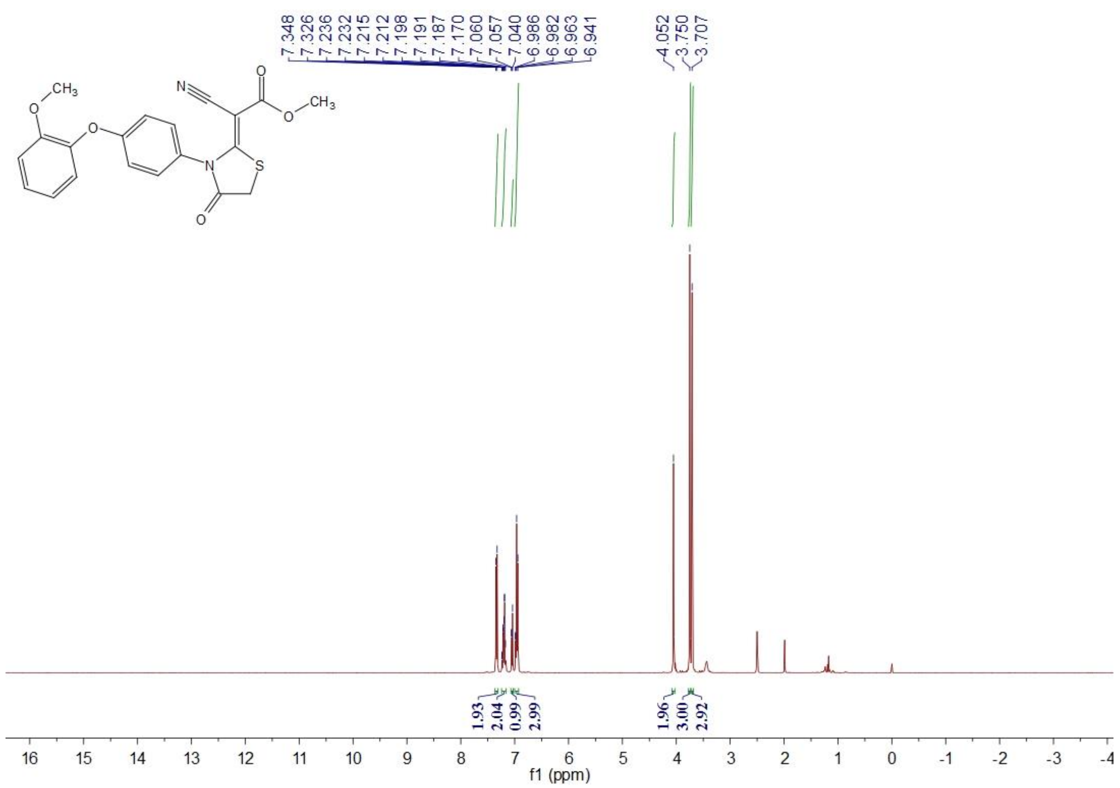

FigureS33.^{1}H-NMR of compound **20.**

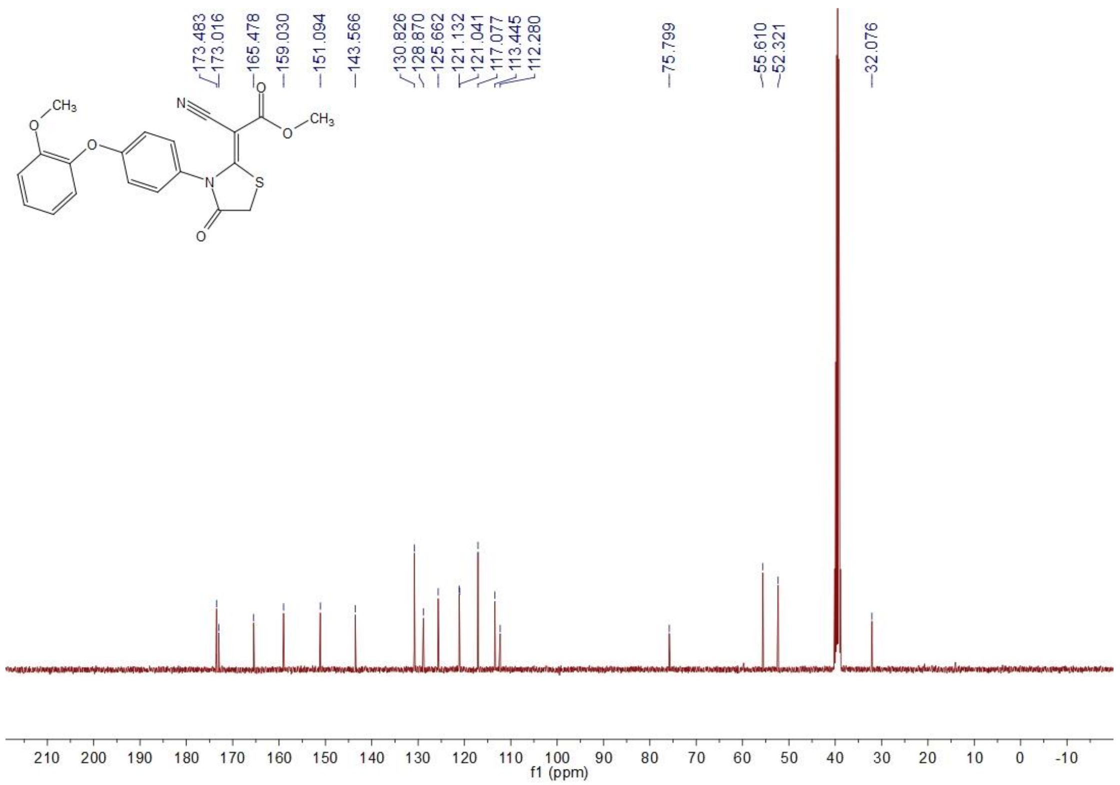

FigureS34.^{13}C-NMR of compound **20.**

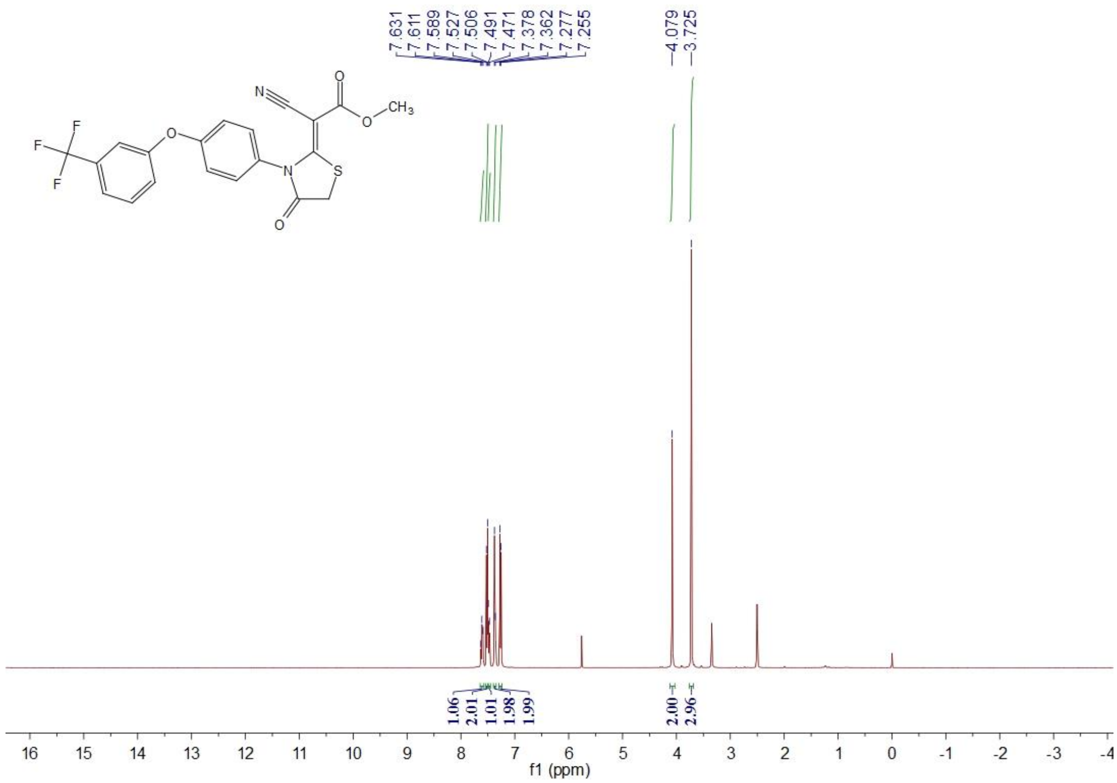

FigureS35.^{1}H-NMR of compound **21.**

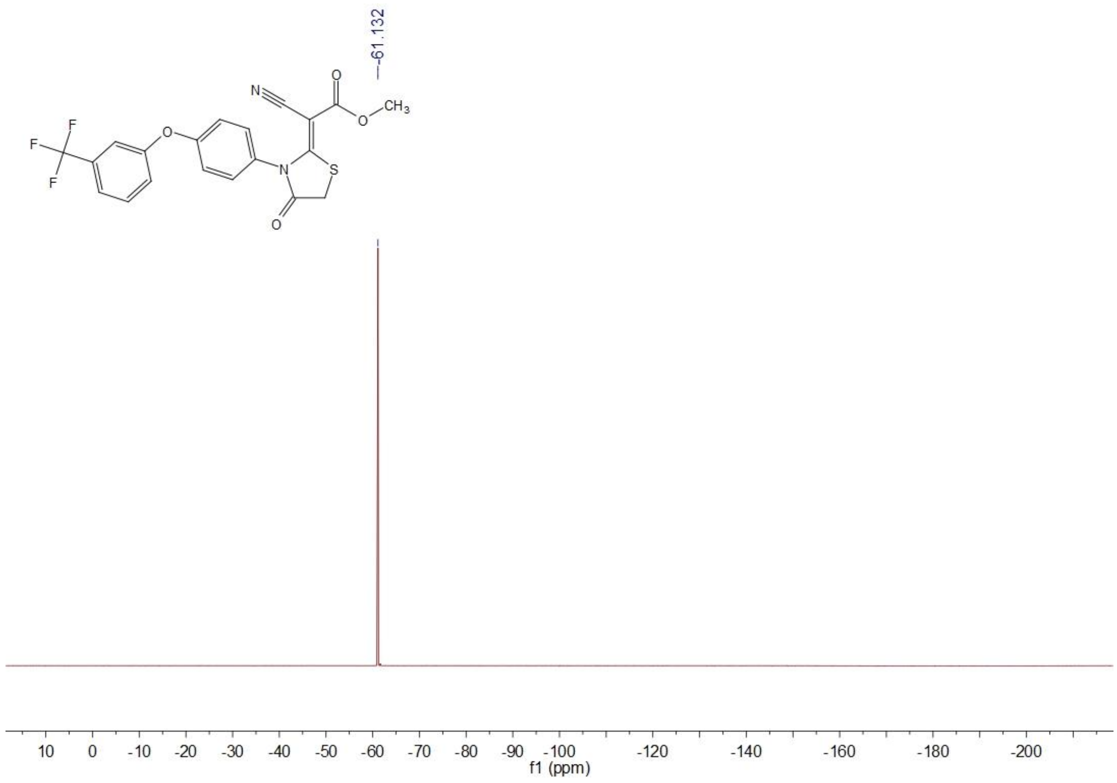

FigureS36.^{19}F-NMR of compound **21.**

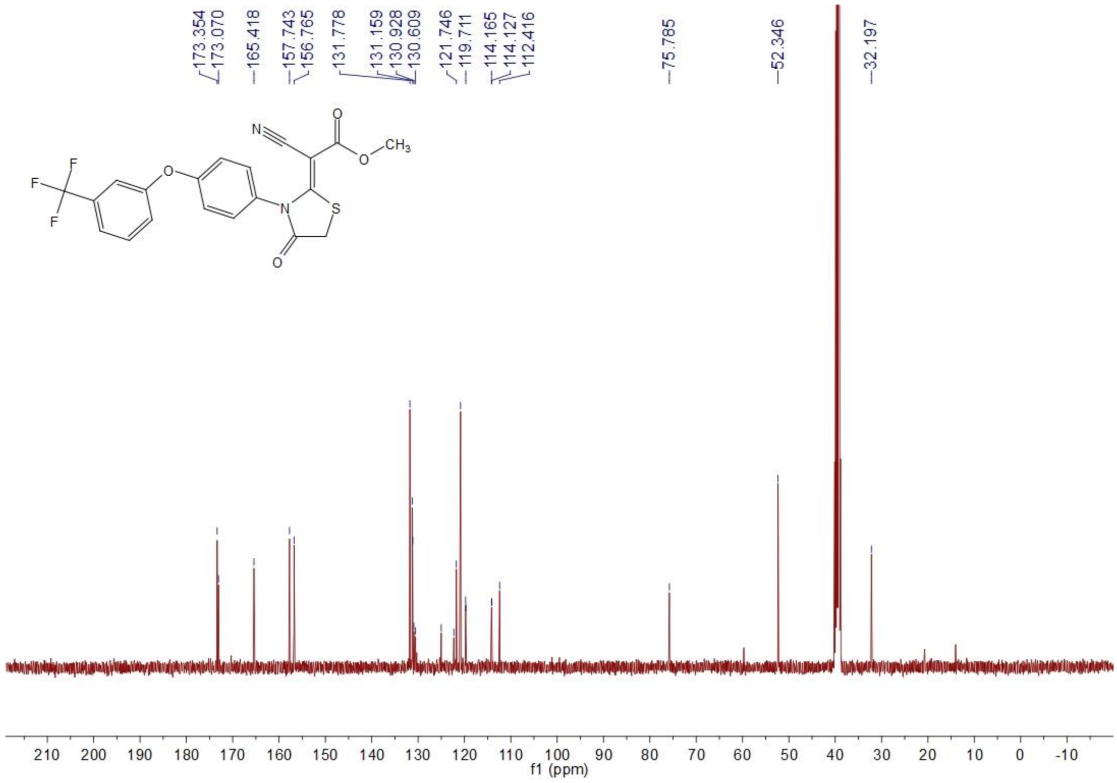

FigureS37.^{13}C-NMR of compound **21.**

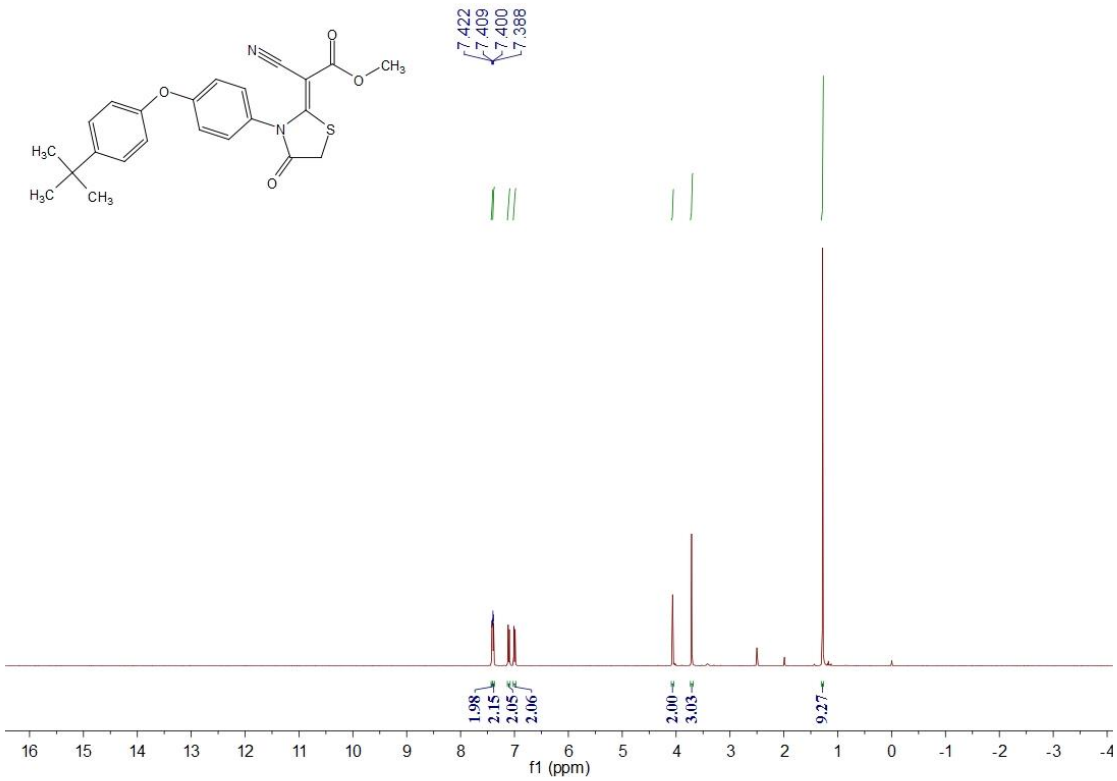

FigureS38.^{1}H-NMR of compound **22.**

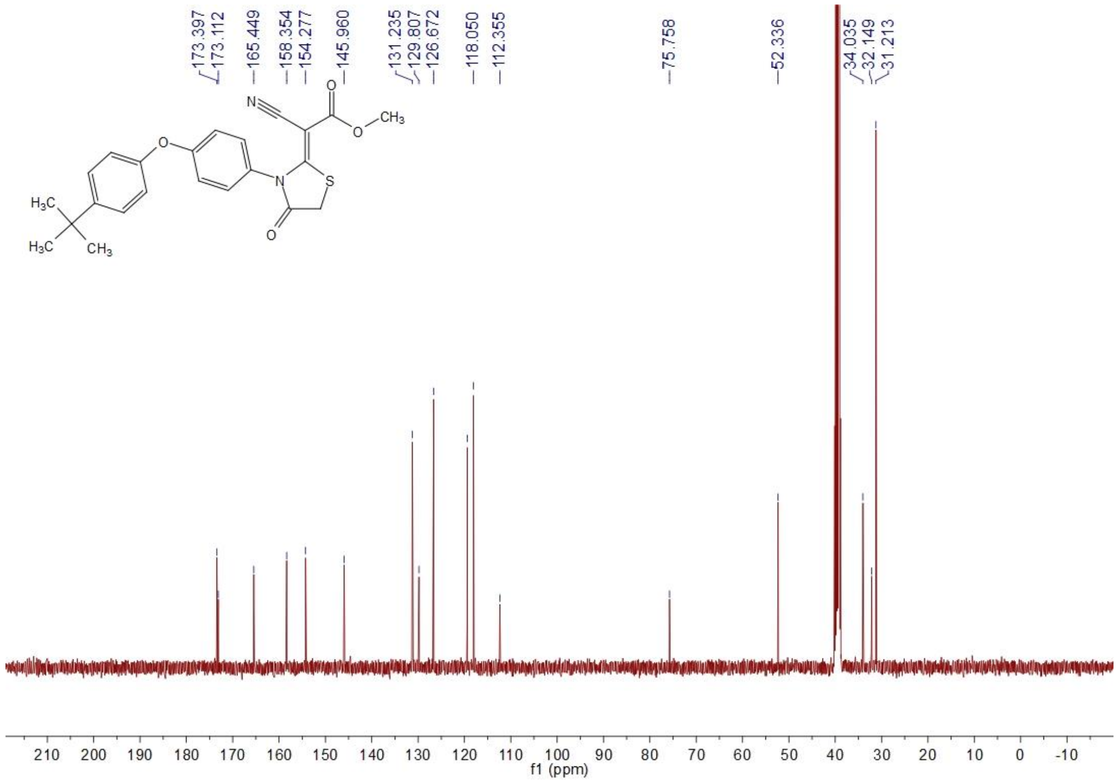

FigureS39.^{13}C-NMR of compound **22.**

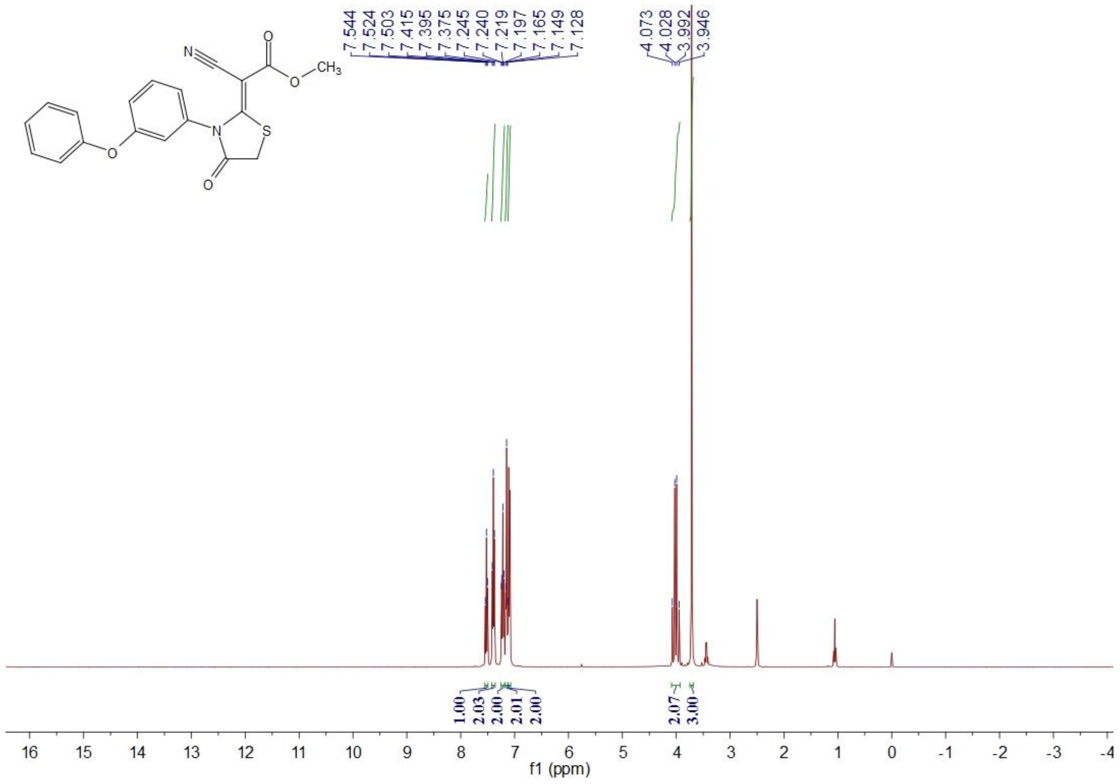

FigureS40.^{1}H-NMR of compound **23.**

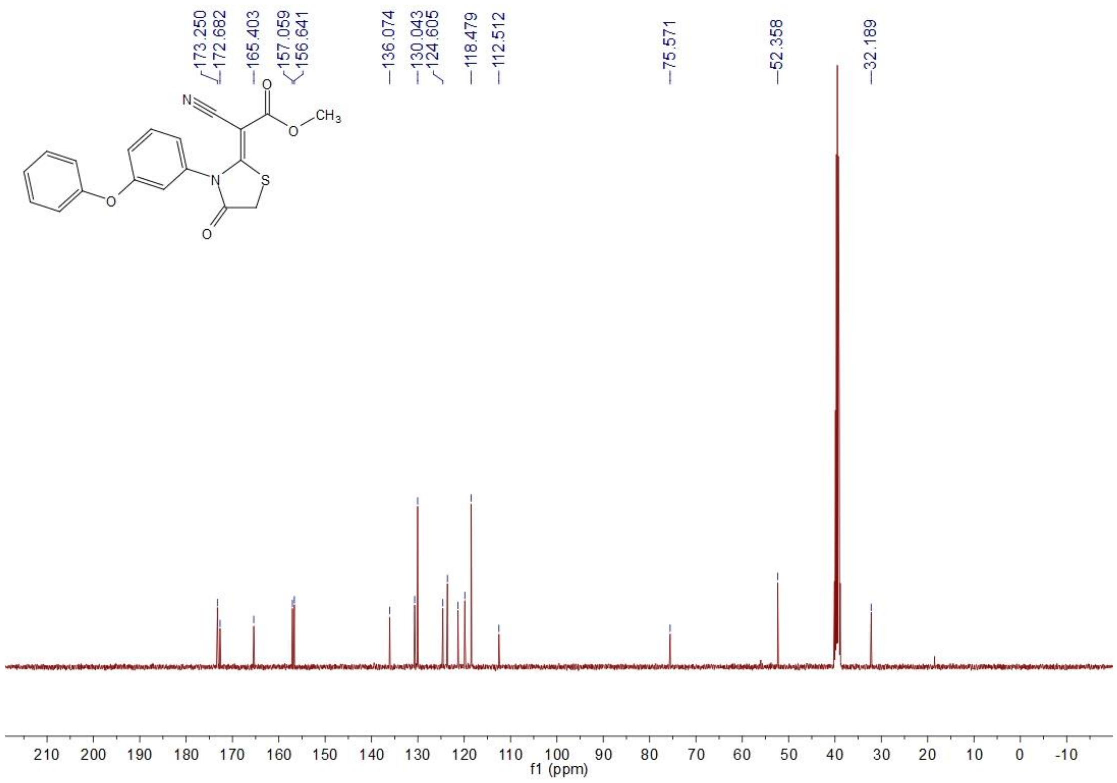

FigureS41.^{13}C-NMR of compound **23.**

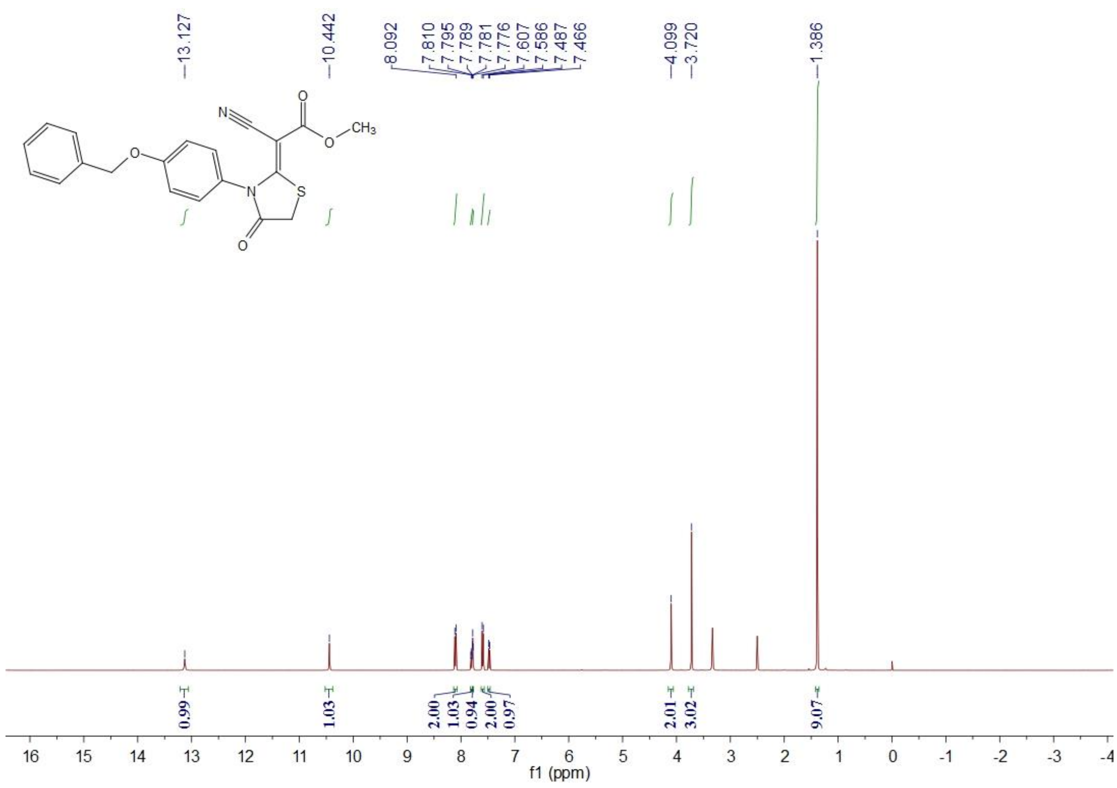

FigureS42.^{1}H-NMR of compound **24.**

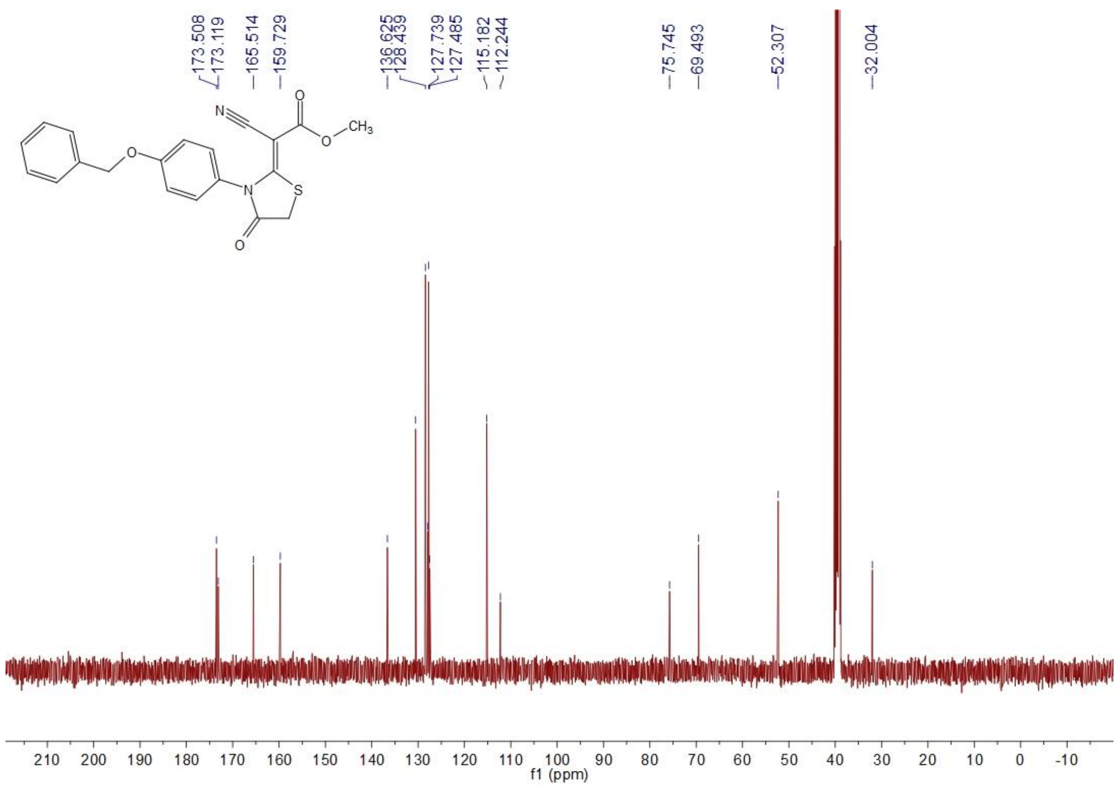

FigureS43.^{13}C-NMR of compound **24.**

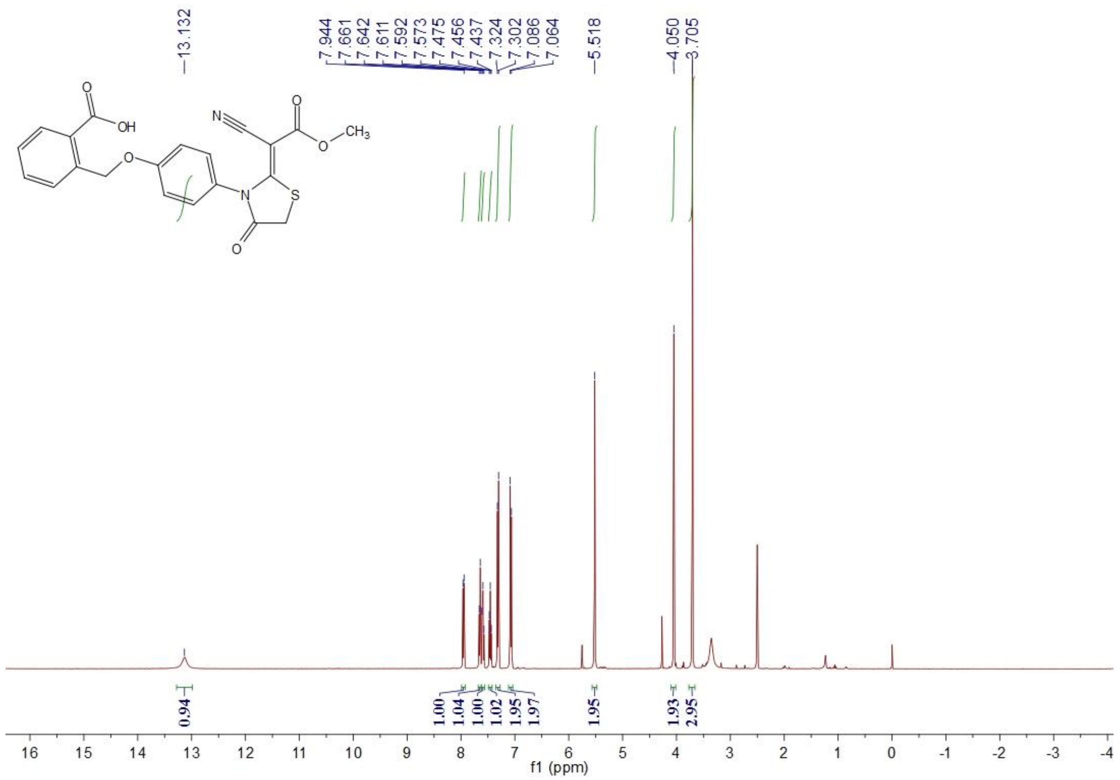

FigureS44.^{1}H-NMR of compound **25.**

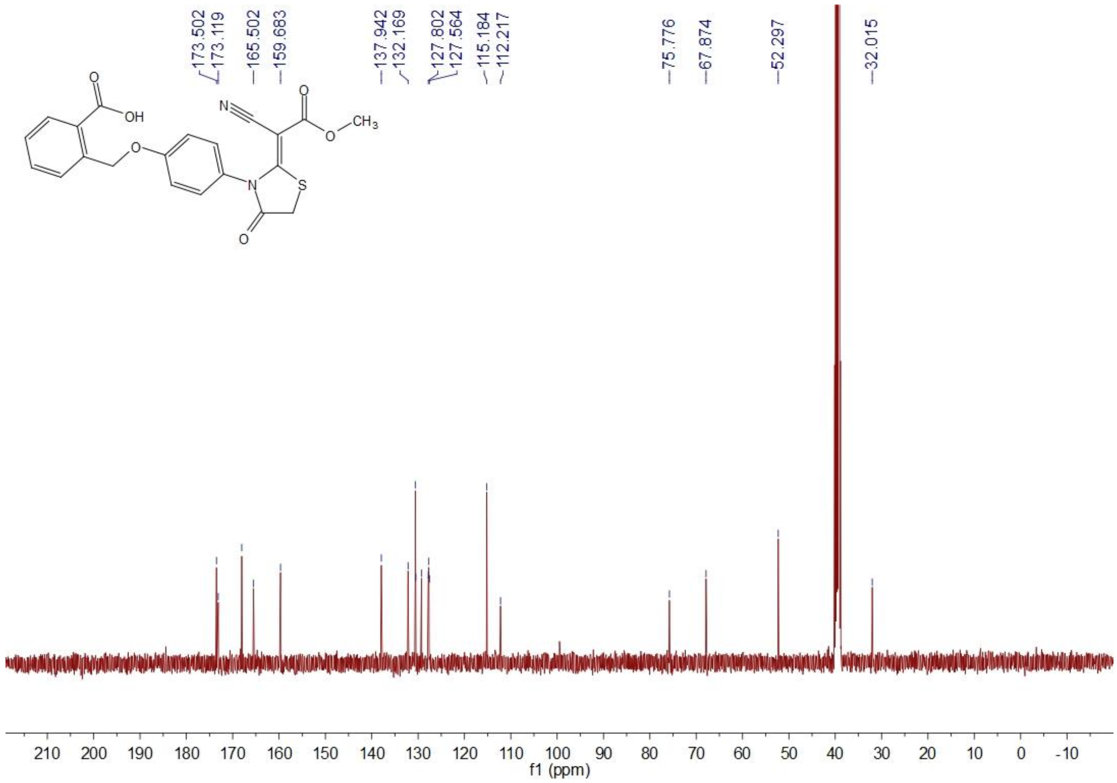

FigureS45.^{13}C-NMR of compound **25.**

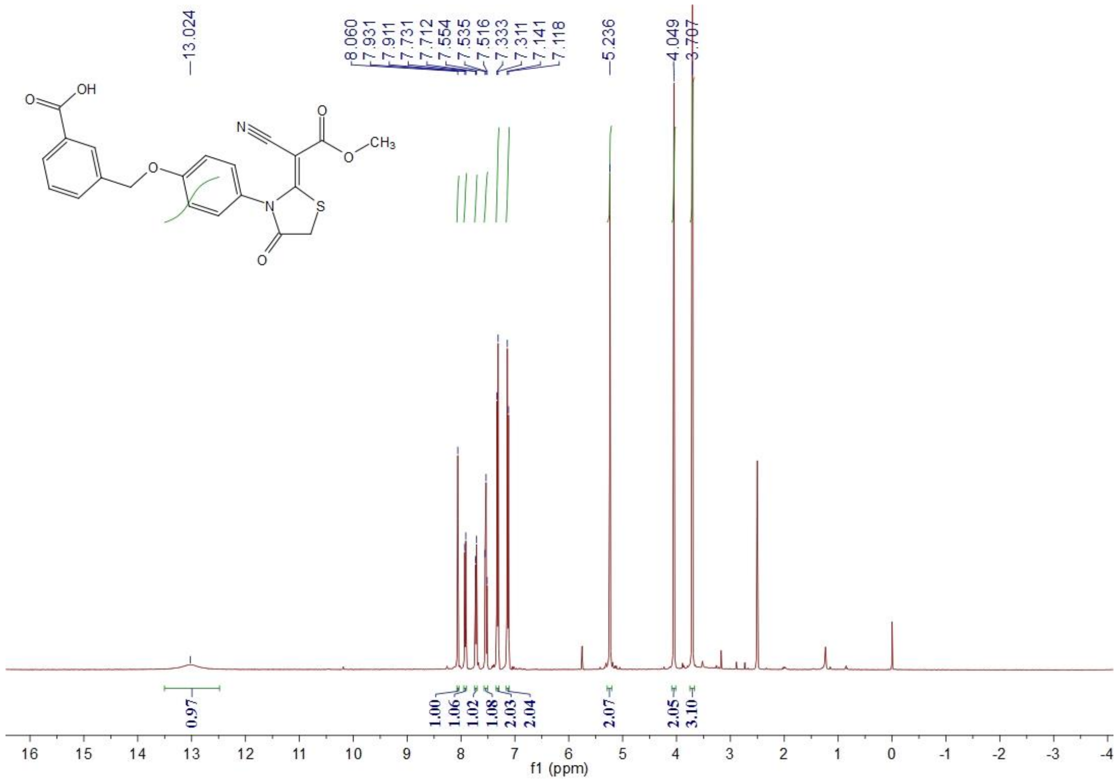

FigureS46.^{1}H-NMR of compound **26.**

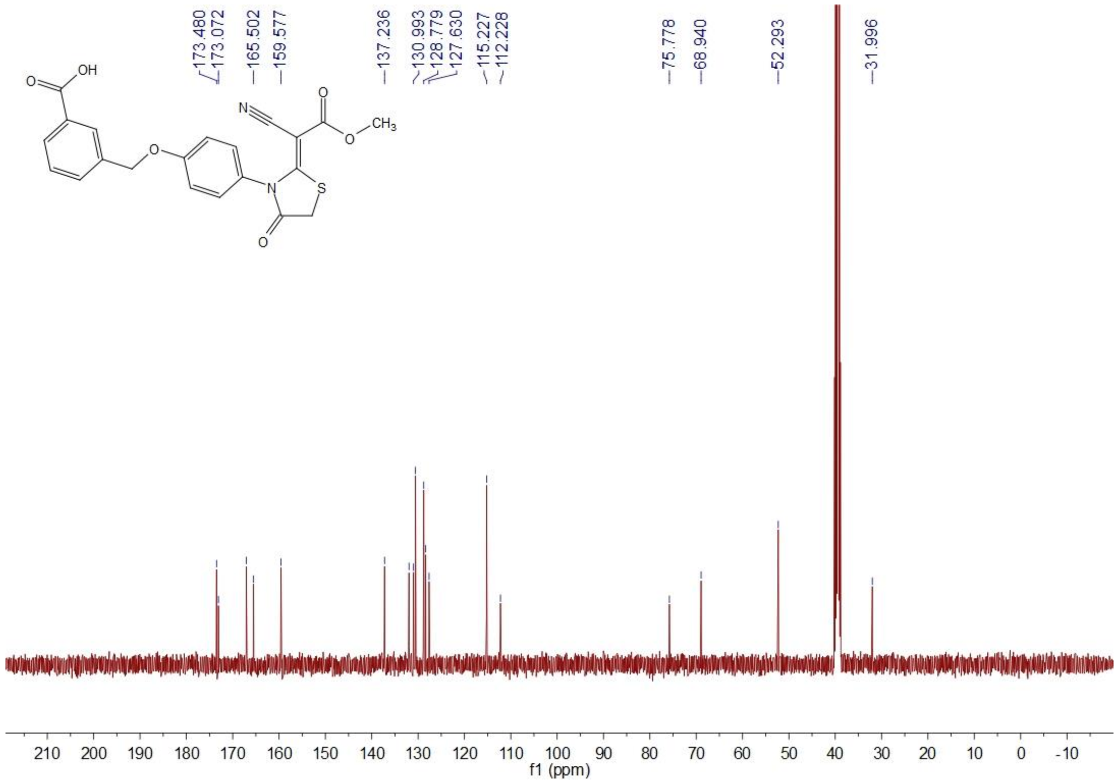

FigureS47.^{13}C-NMR of compound **26.**

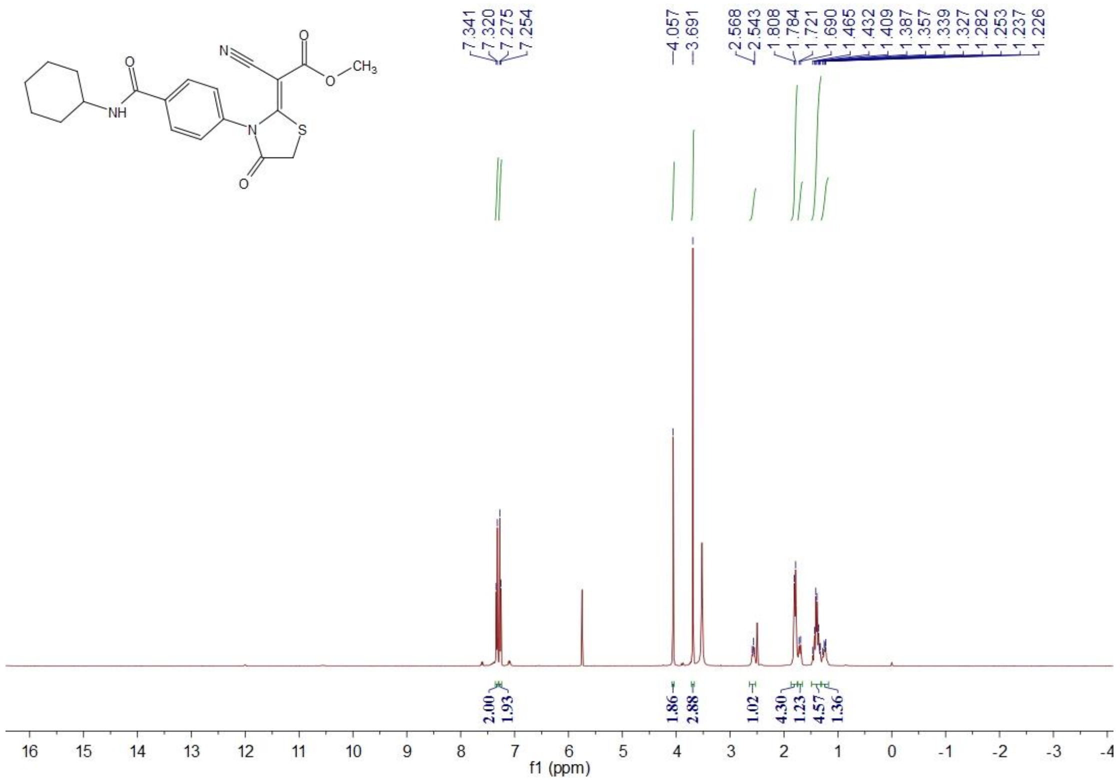

FigureS48.^{1}H-NMR of compound **27.**

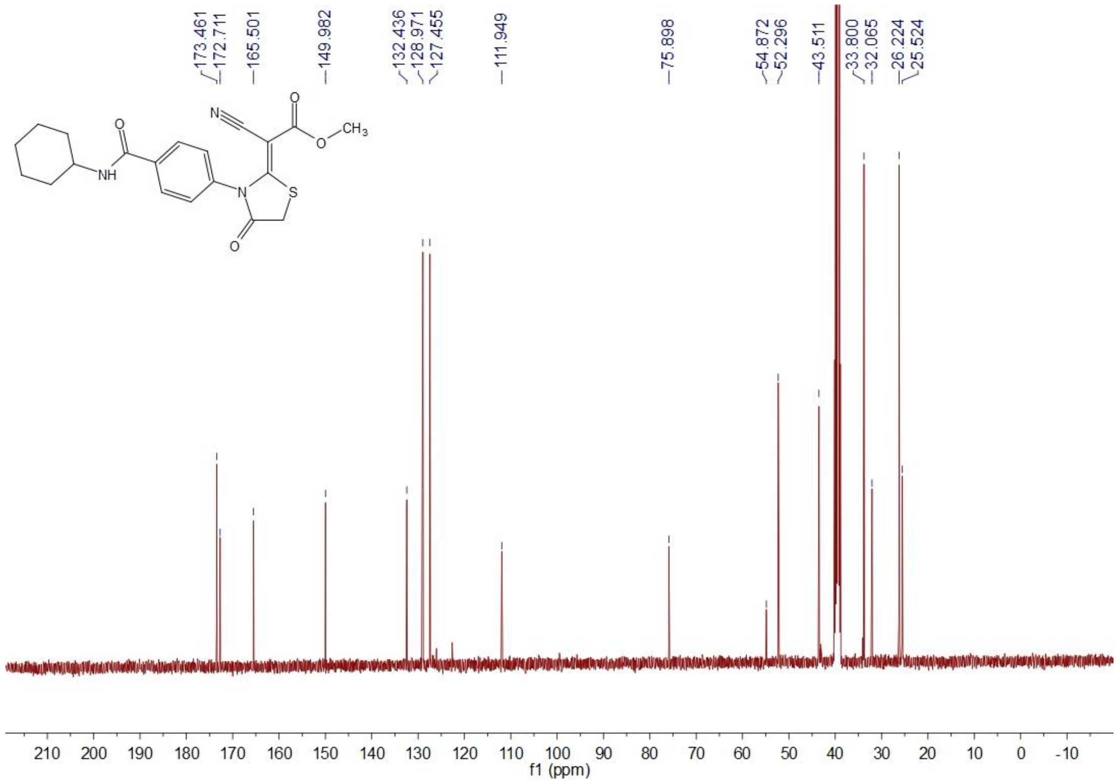

FigureS49.13C-NMR of compound **27.**

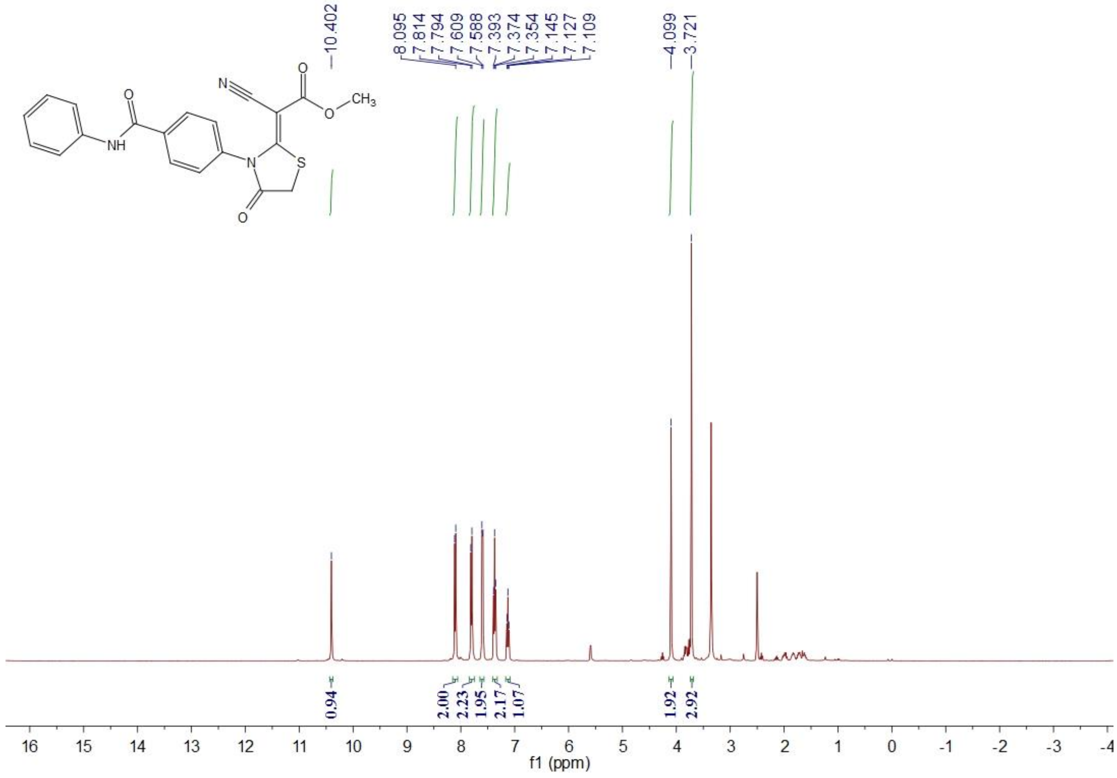

FigureS50.1H-NMR of compound **28.**

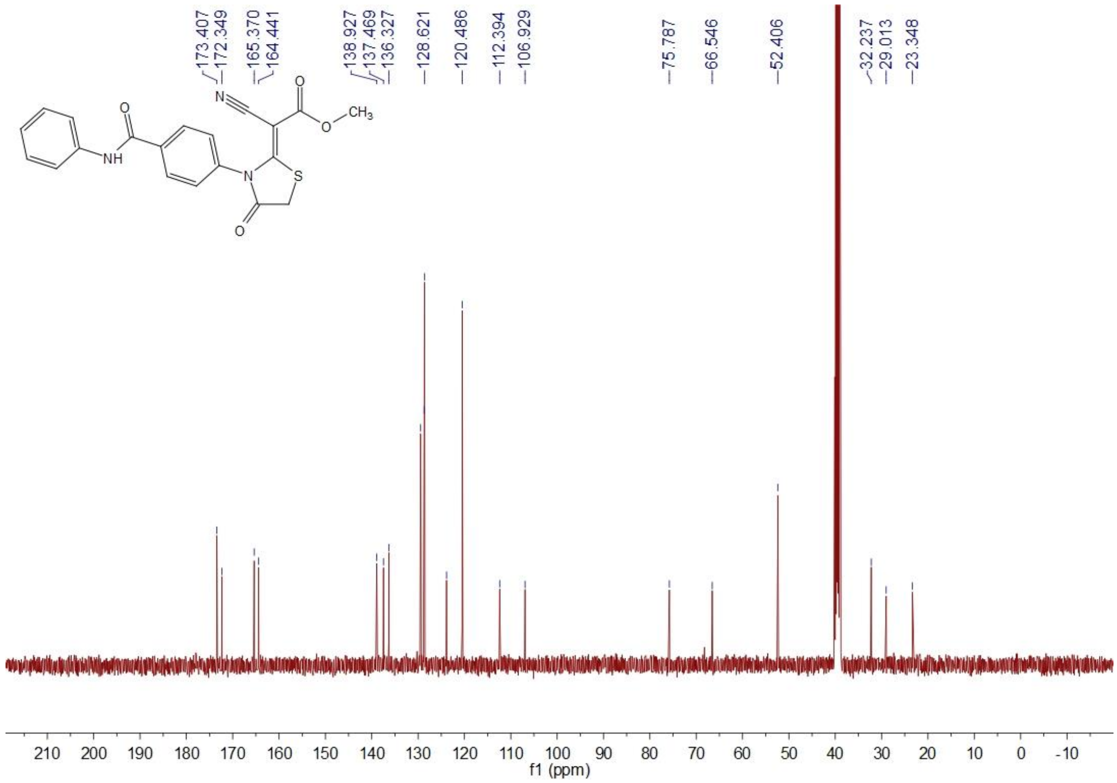

FigureS51.^{13}C-NMR of compound **28.**

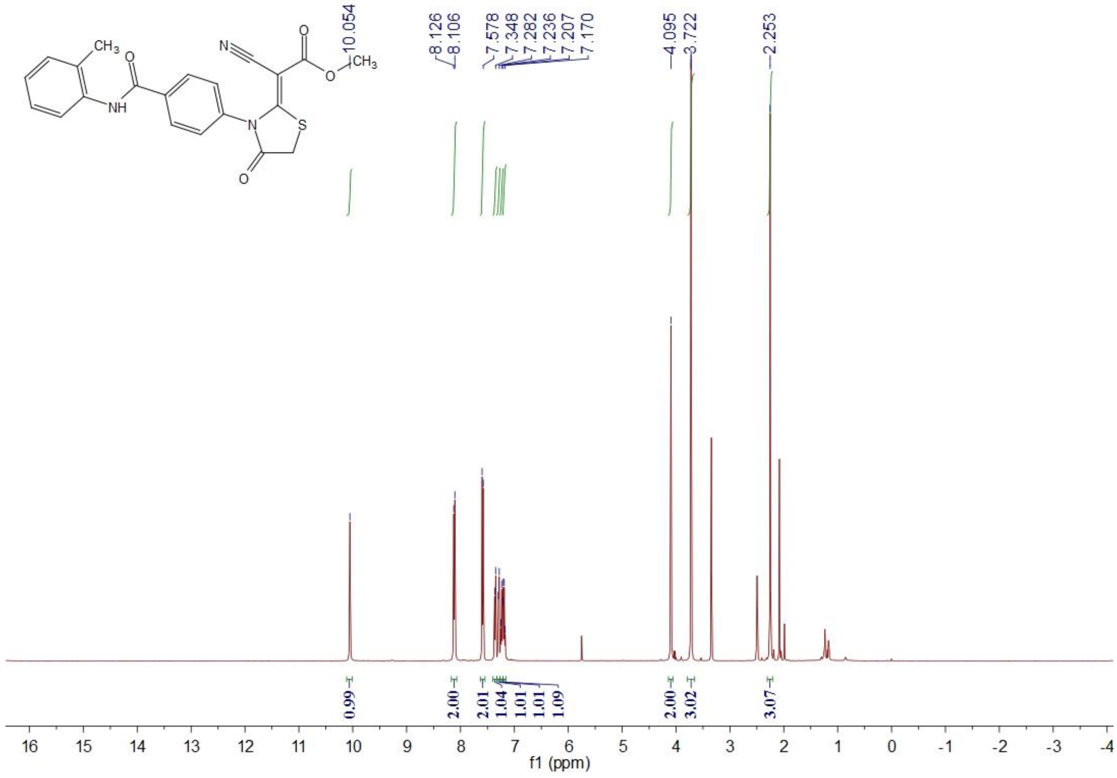

FigureS52.^{1}H-NMR of compound **29.**

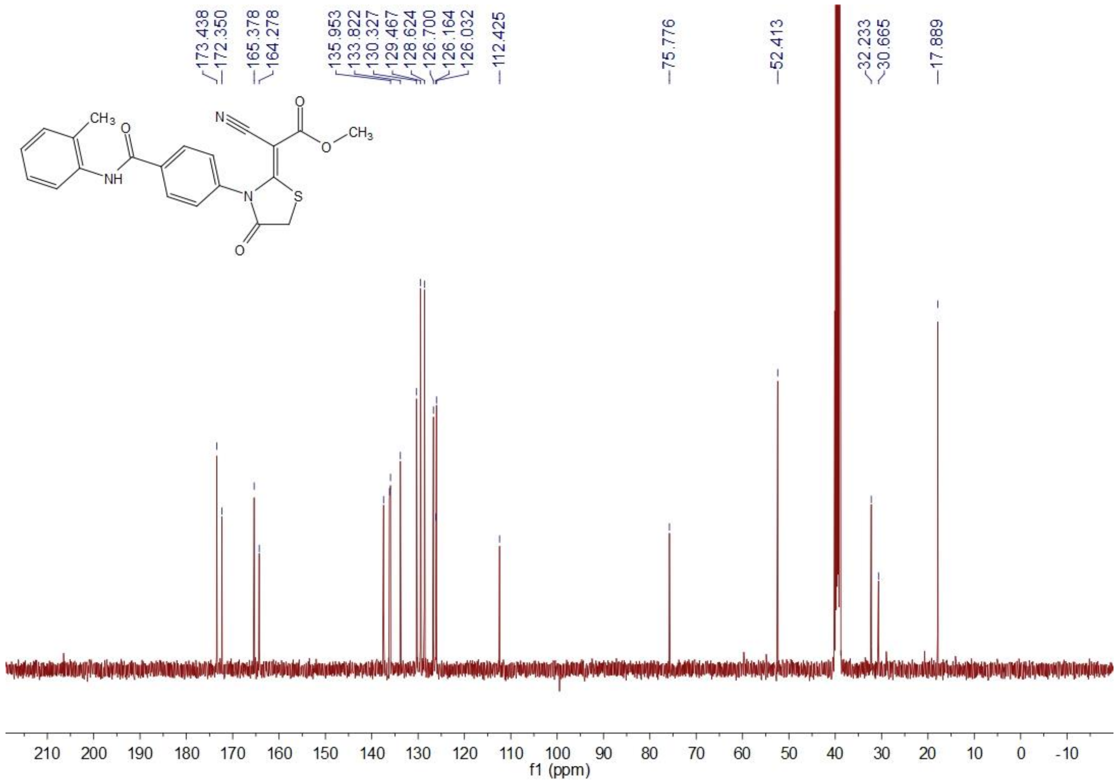

FigureS53.^{13}C-NMR of compound **29.**

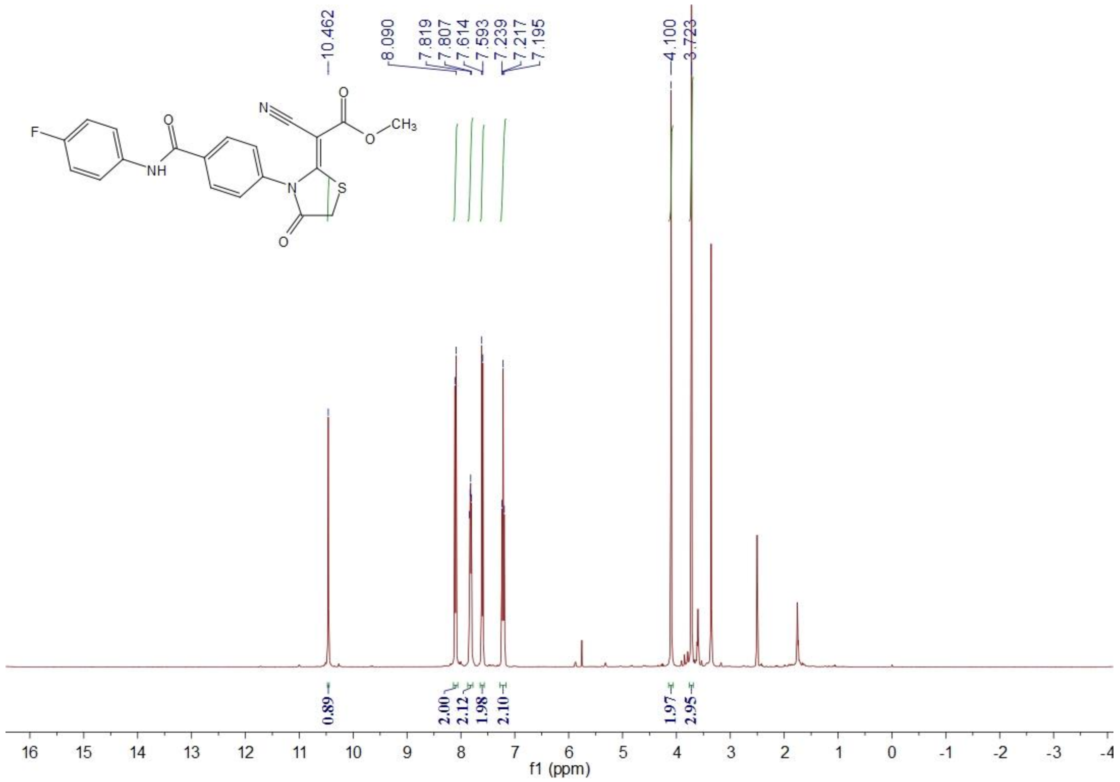

FigureS54.^{1}H-NMR of compound **30.**

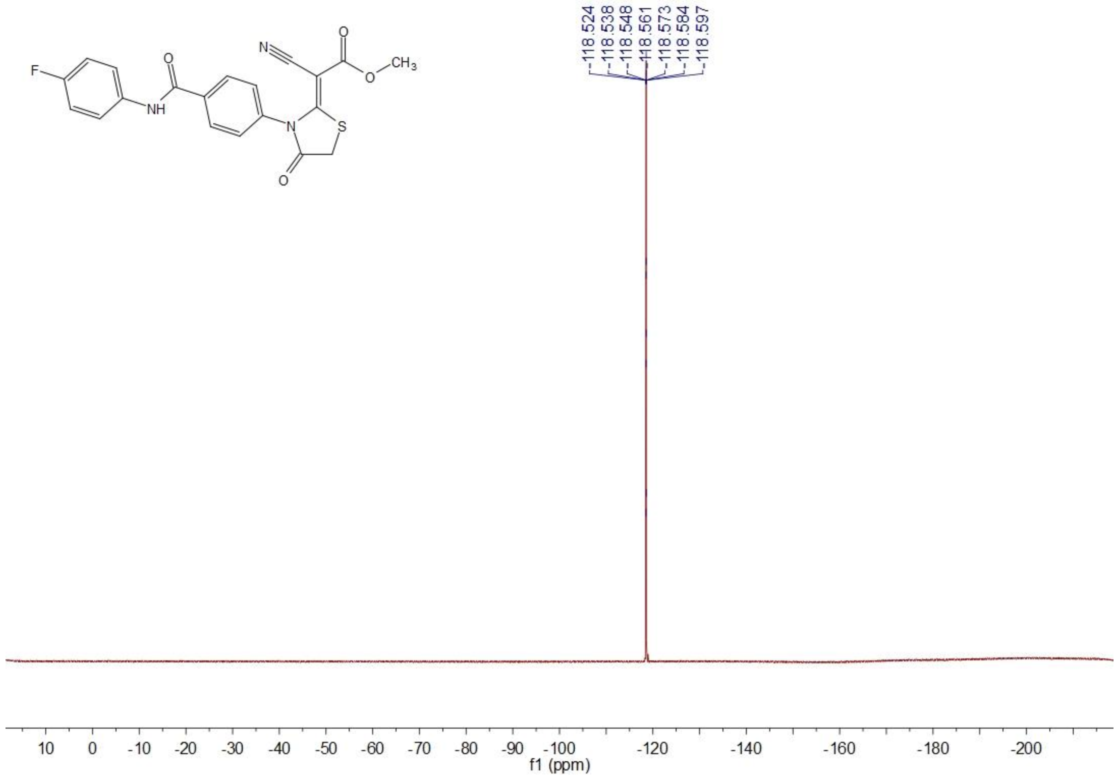

FigureS55.^{19}F-NMR of compound **30.**

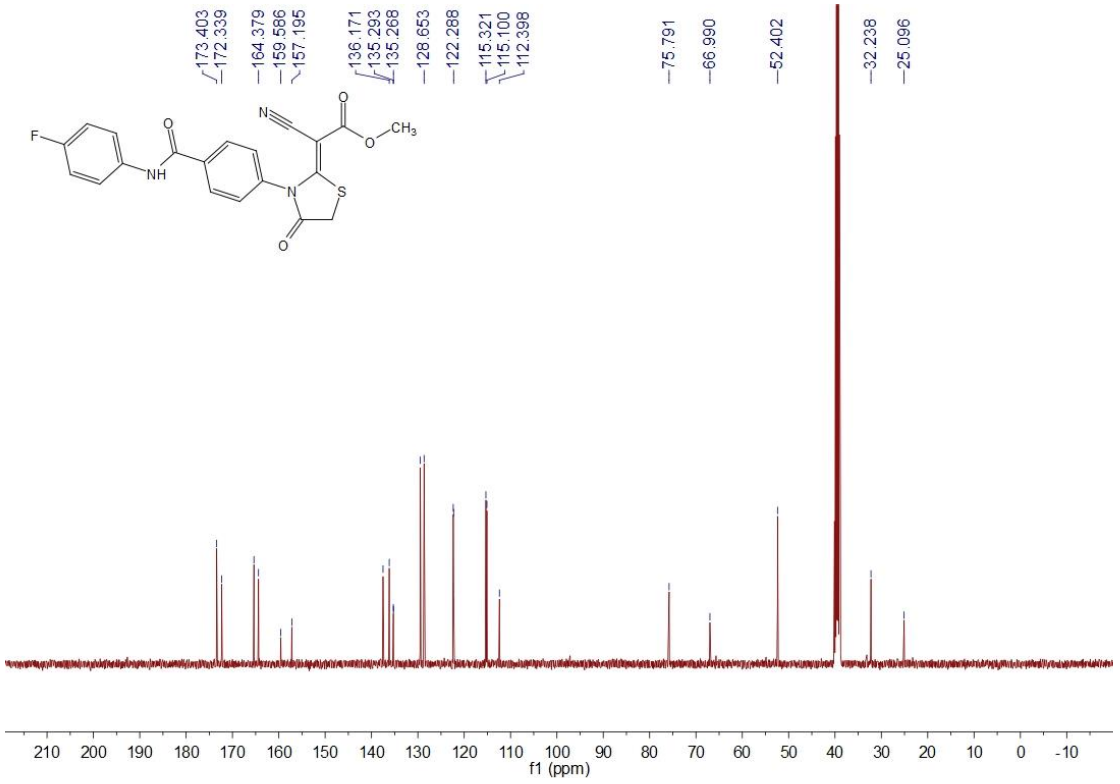

FigureS56.^{13}C-NMR of compound **30.**

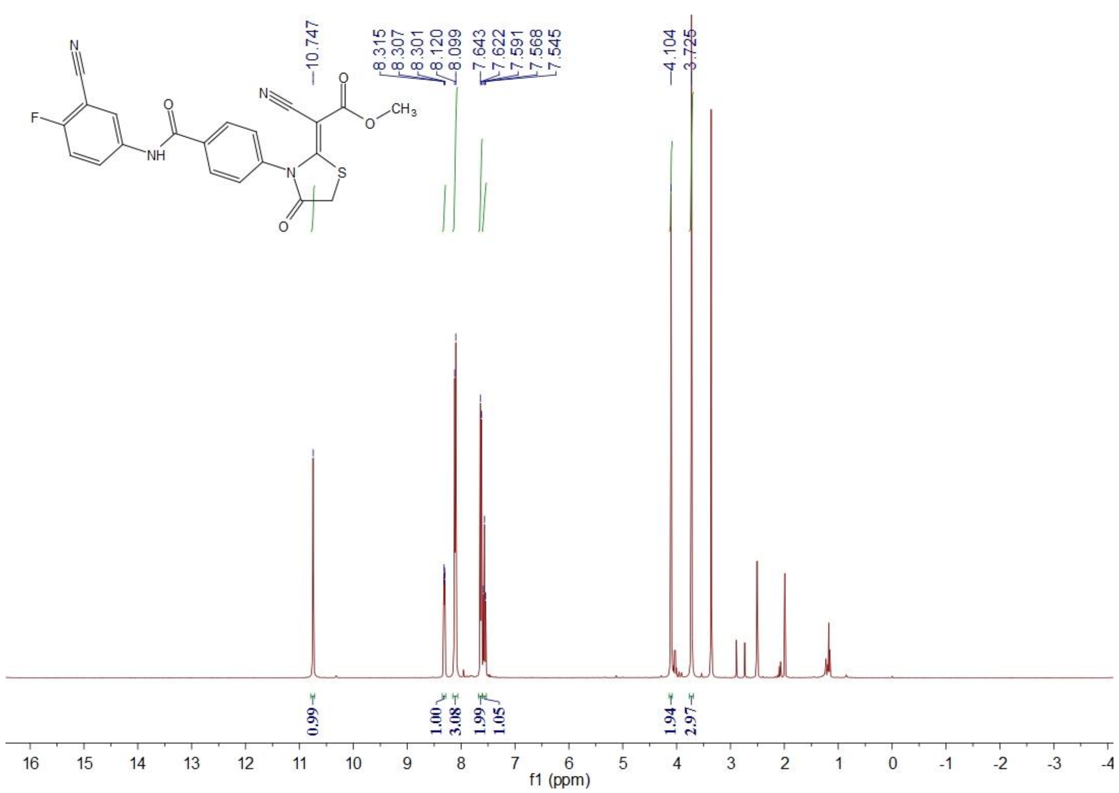

FigureS57.^{1}H-NMR of compound **31.**

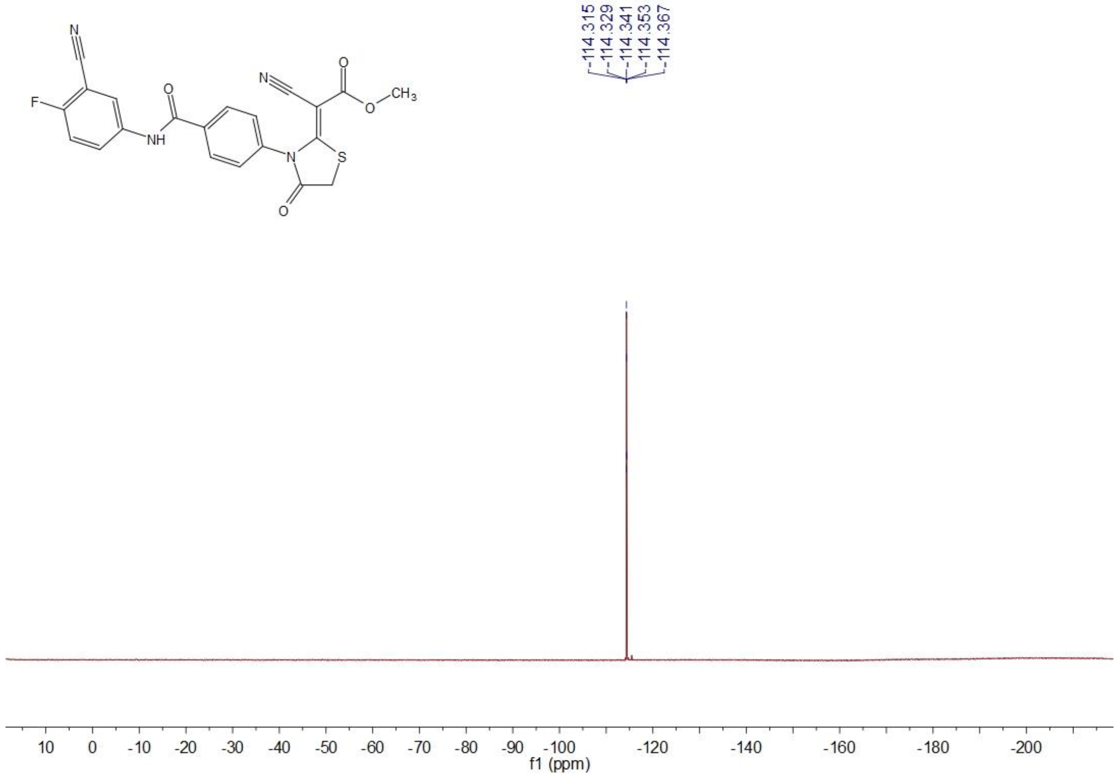

FigureS58.^{19}F-NMR of compound **31.**

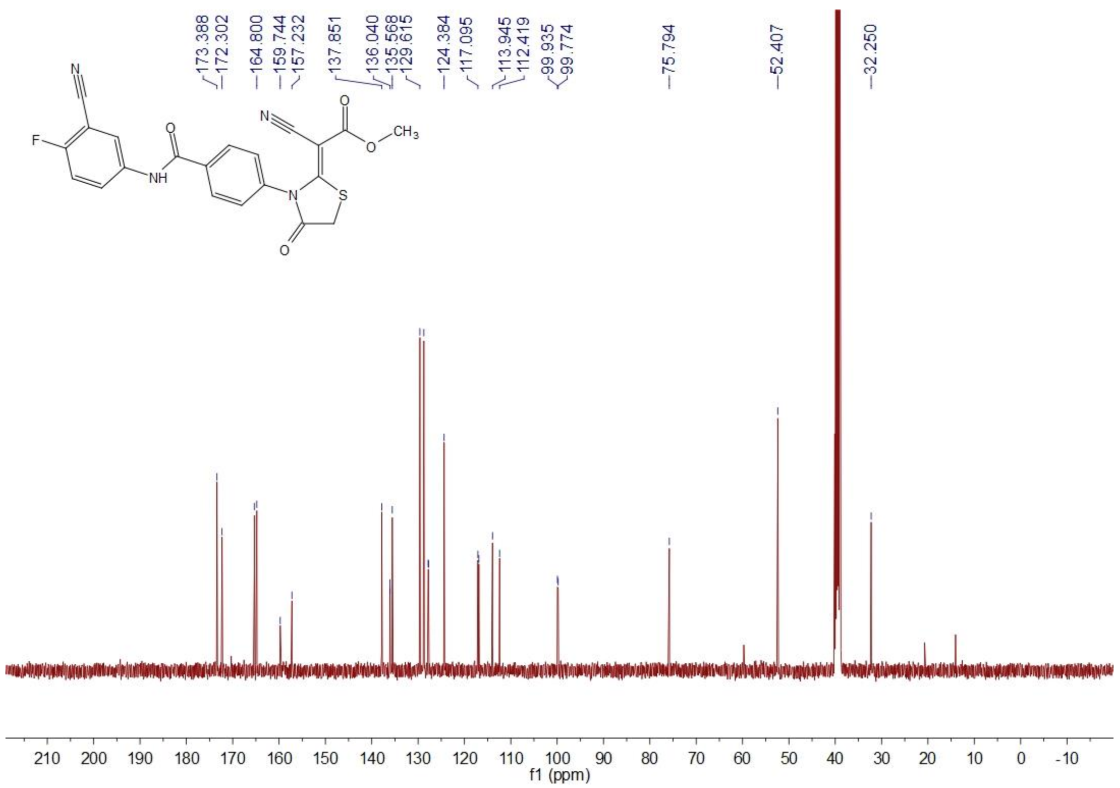

FigureS59.^{13}C-NMR of compound **31.**

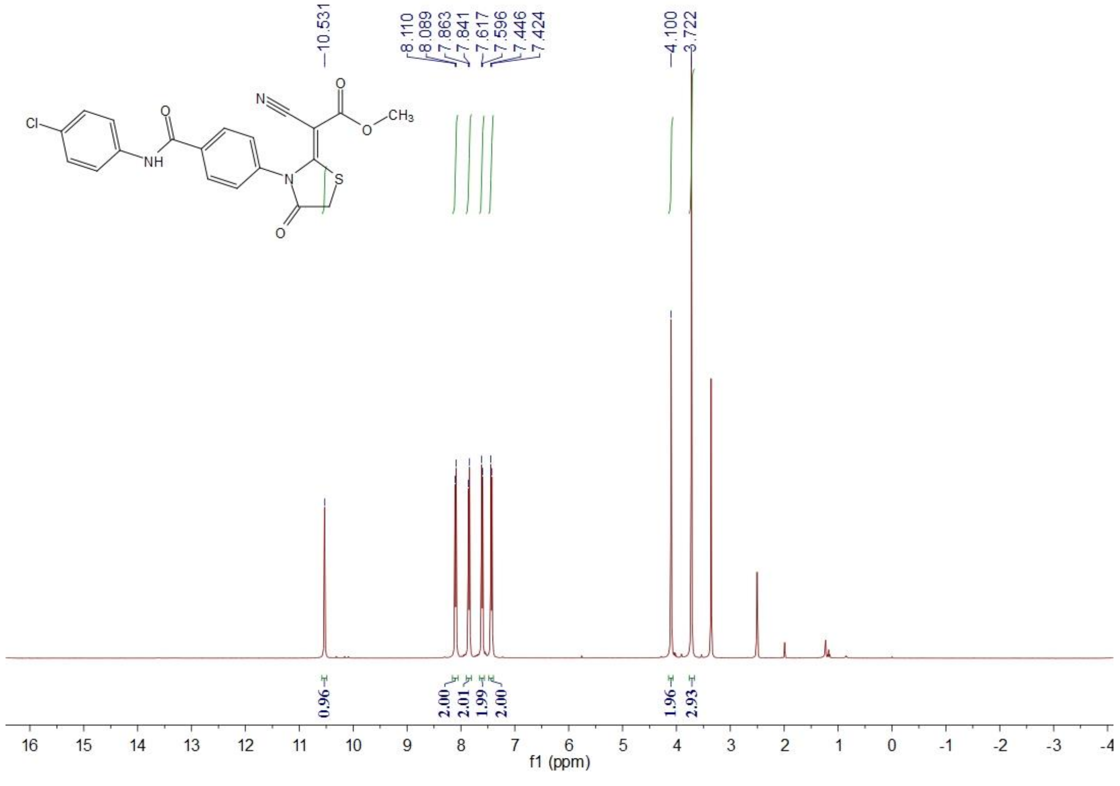

FigureS60.^{1}H-NMR of compound **32.**

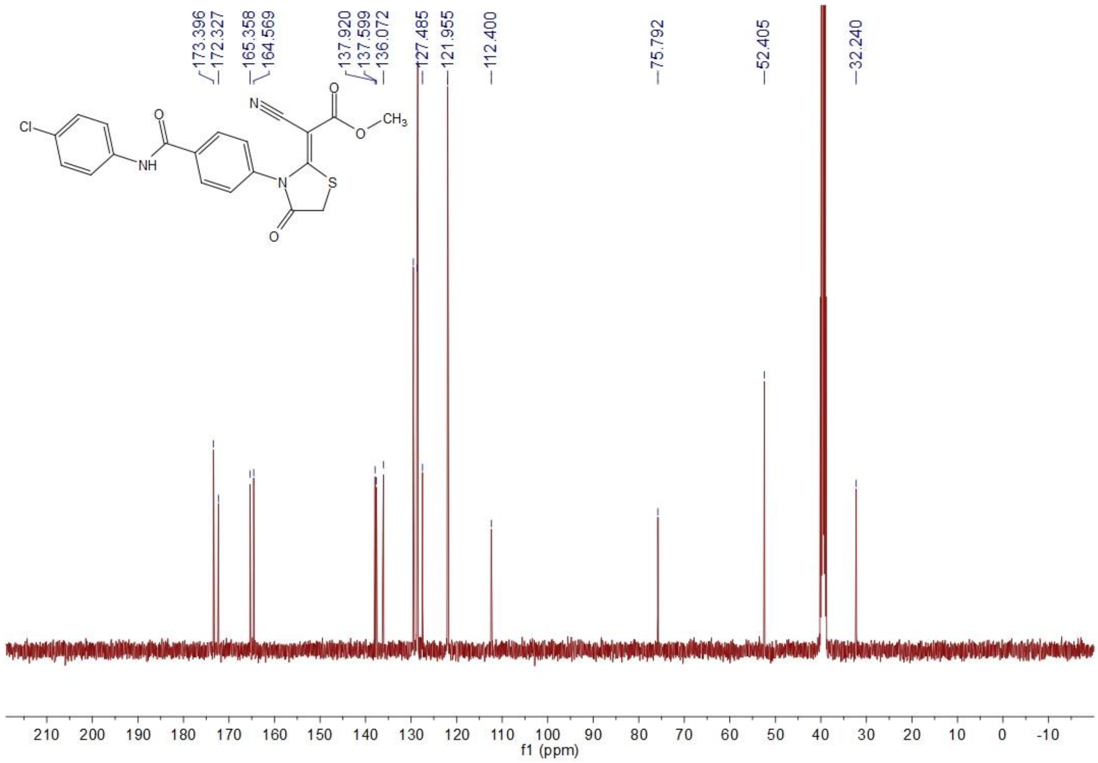

FigureS61.^{13}C-NMR of compound **32.**

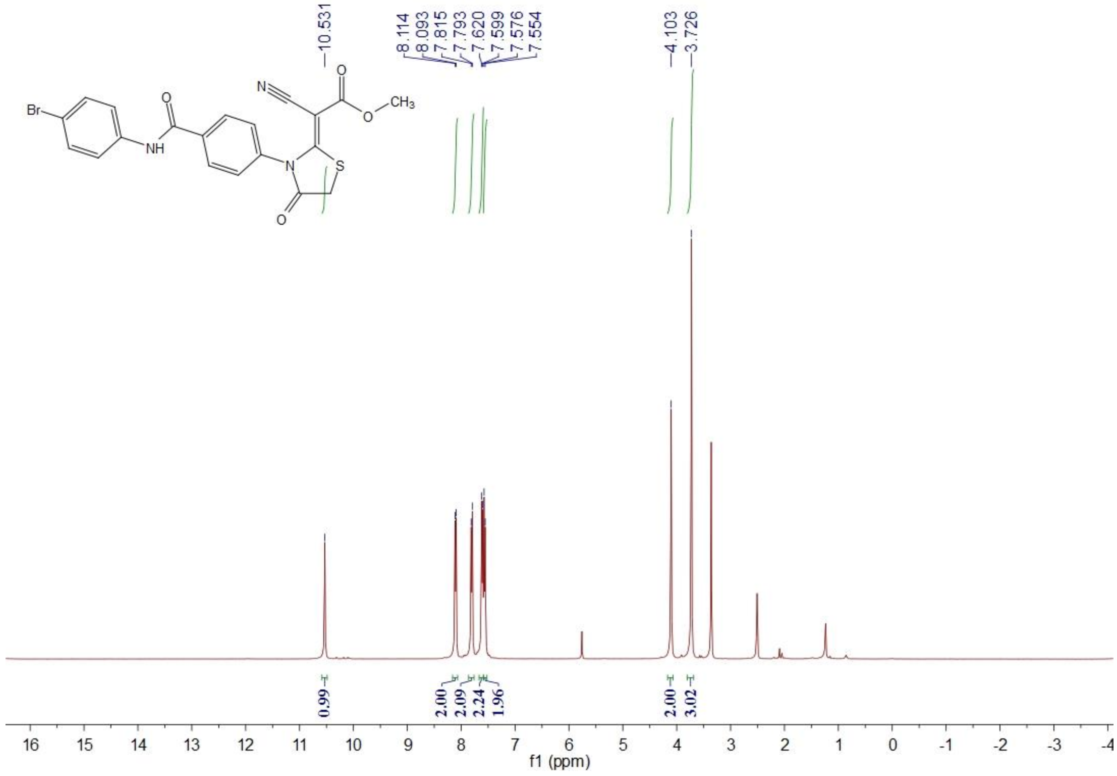

FigureS62.^{1}H-NMR of compound **33.**

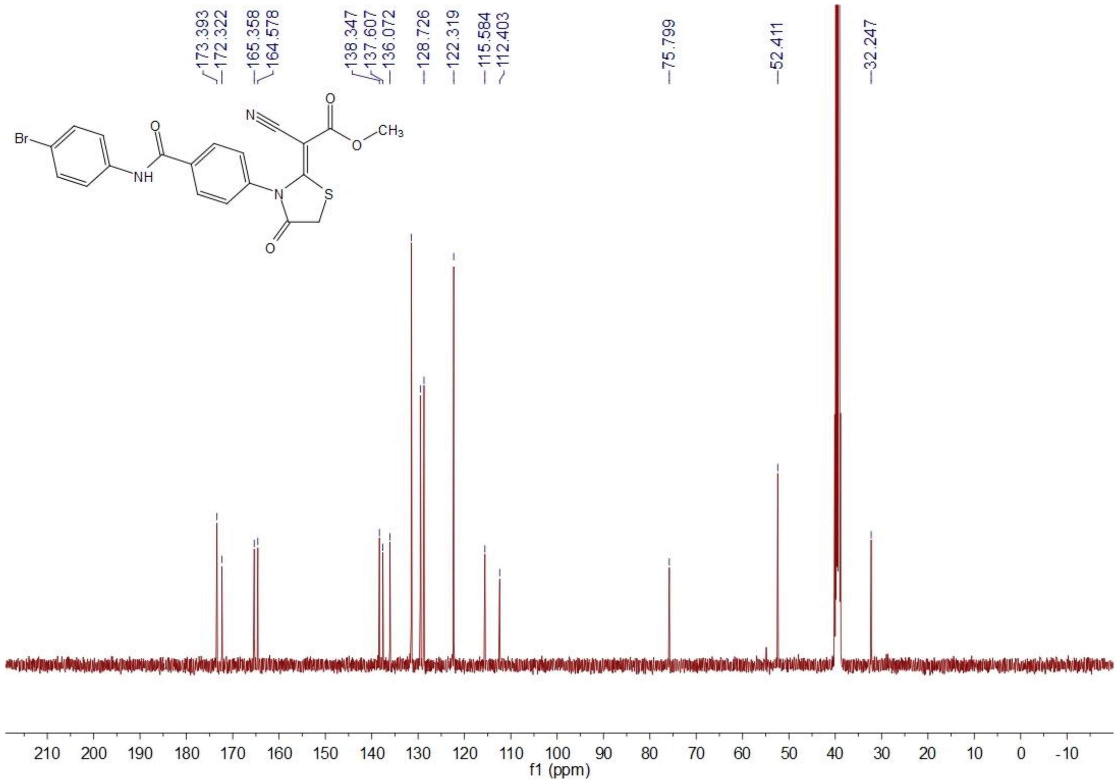

FigureS63.^{13}C-NMR of compound **33.**

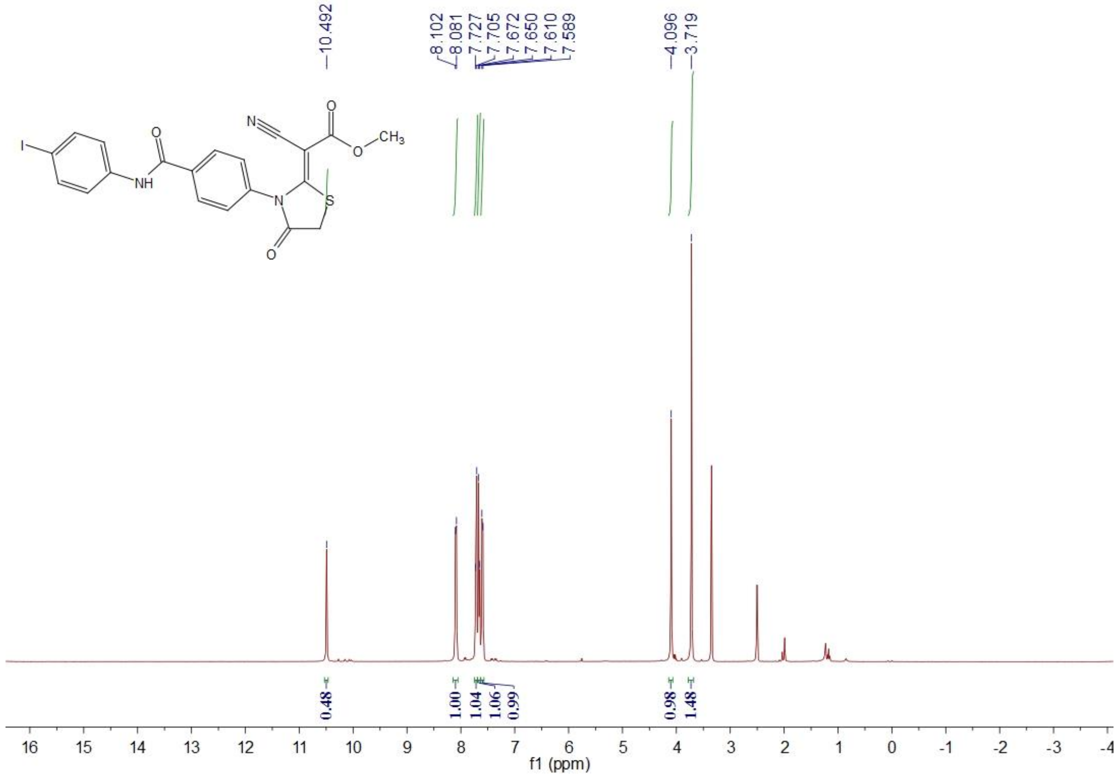

FigureS64.^{1}H-NMR of compound **34.**

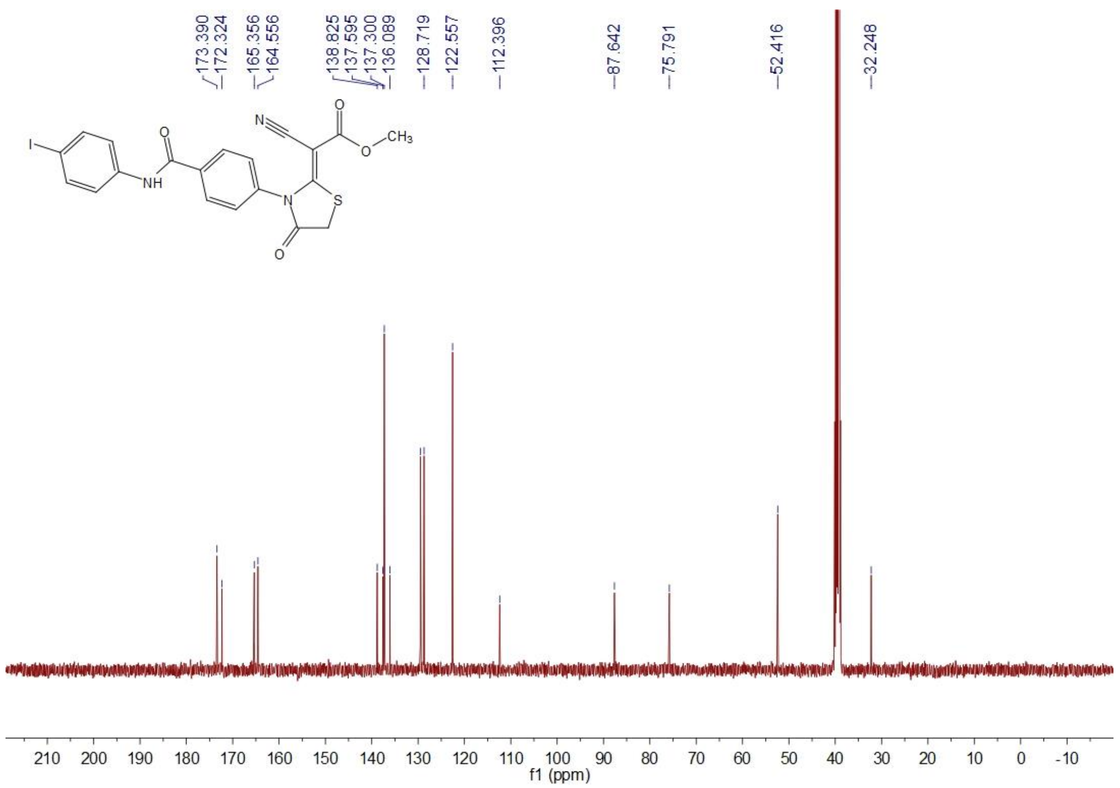

FigureS65.^{13}C-NMR of compound **34.**

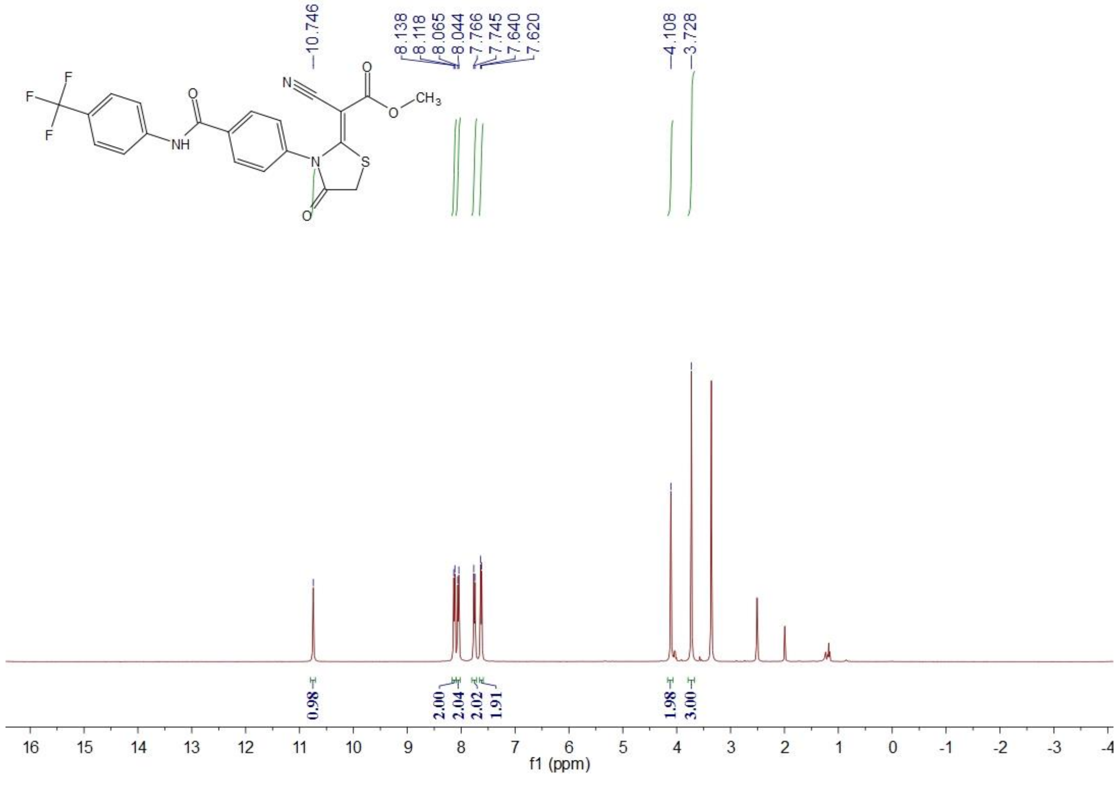

FigureS66.^{1}H-NMR of compound **35.**

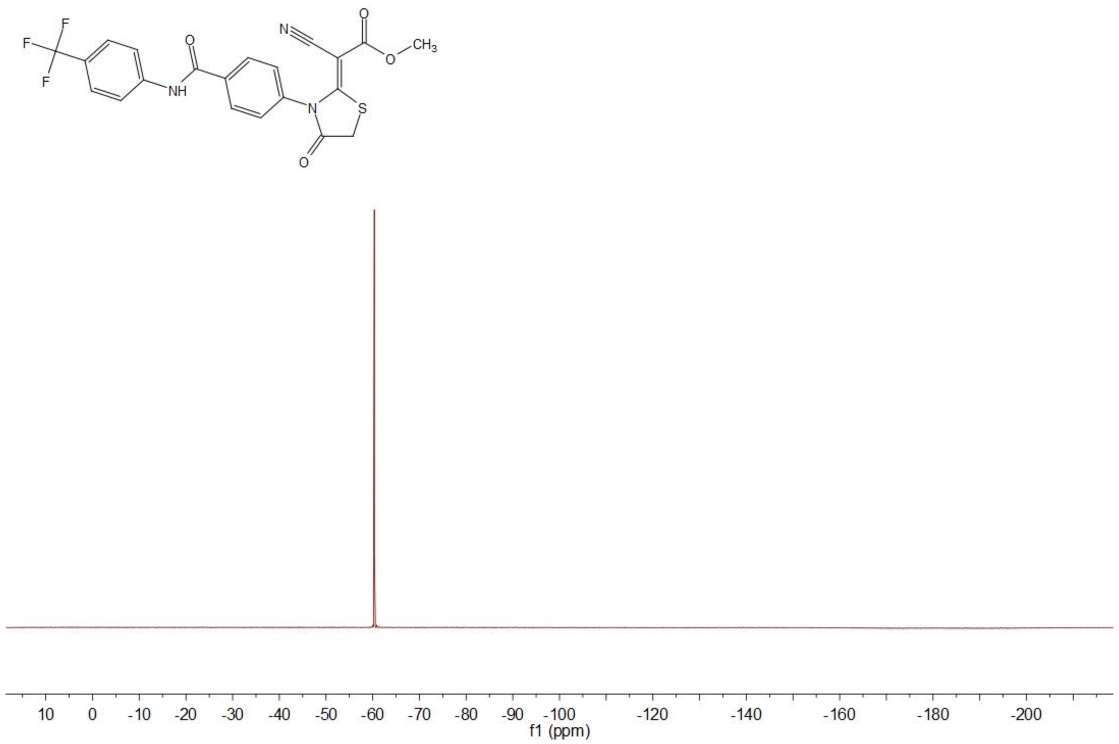

FigureS67.^{19}F-NMR of compound **35.**

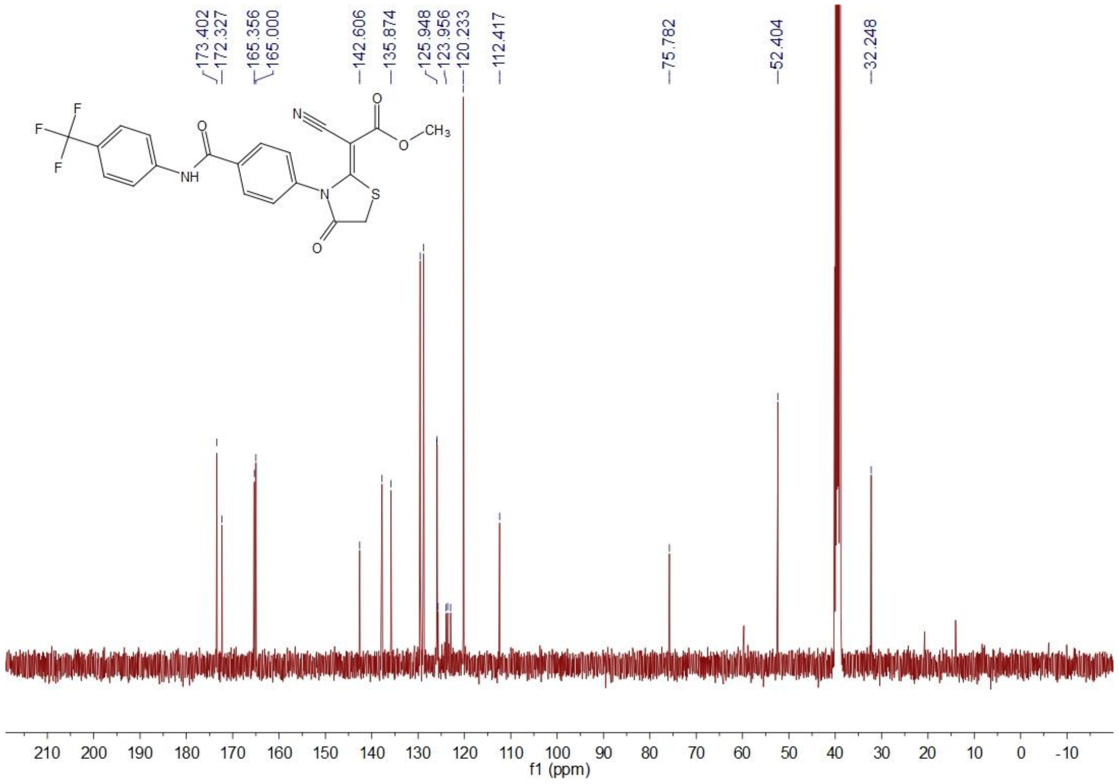

FigureS68.^{13}C-NMR of compound **35.**

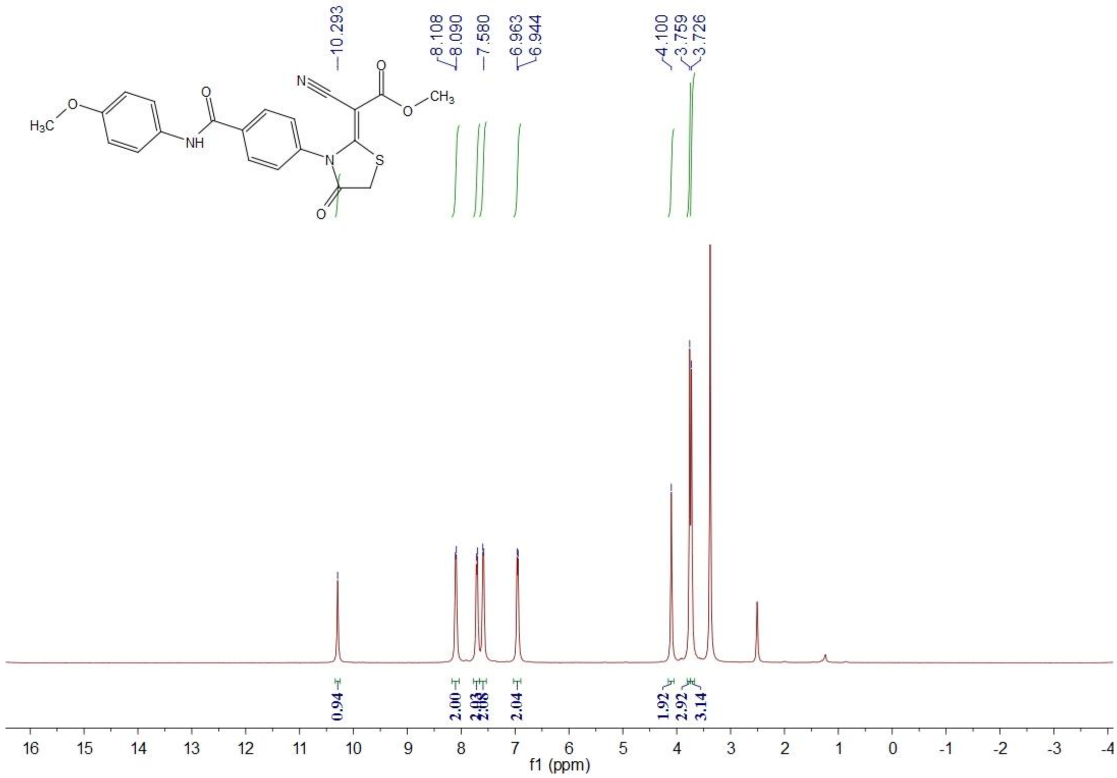

FigureS69.^{1}H-NMR of compound **36.**

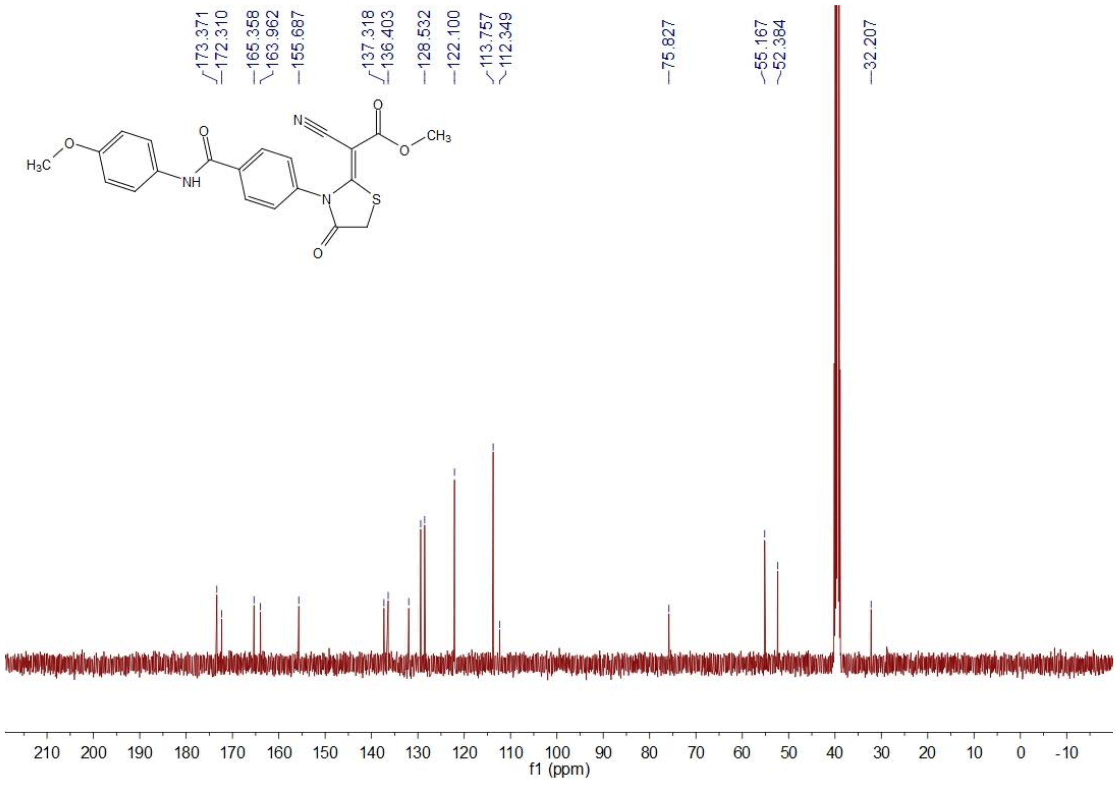

FigureS70.^{13}C-NMR of compound **36**

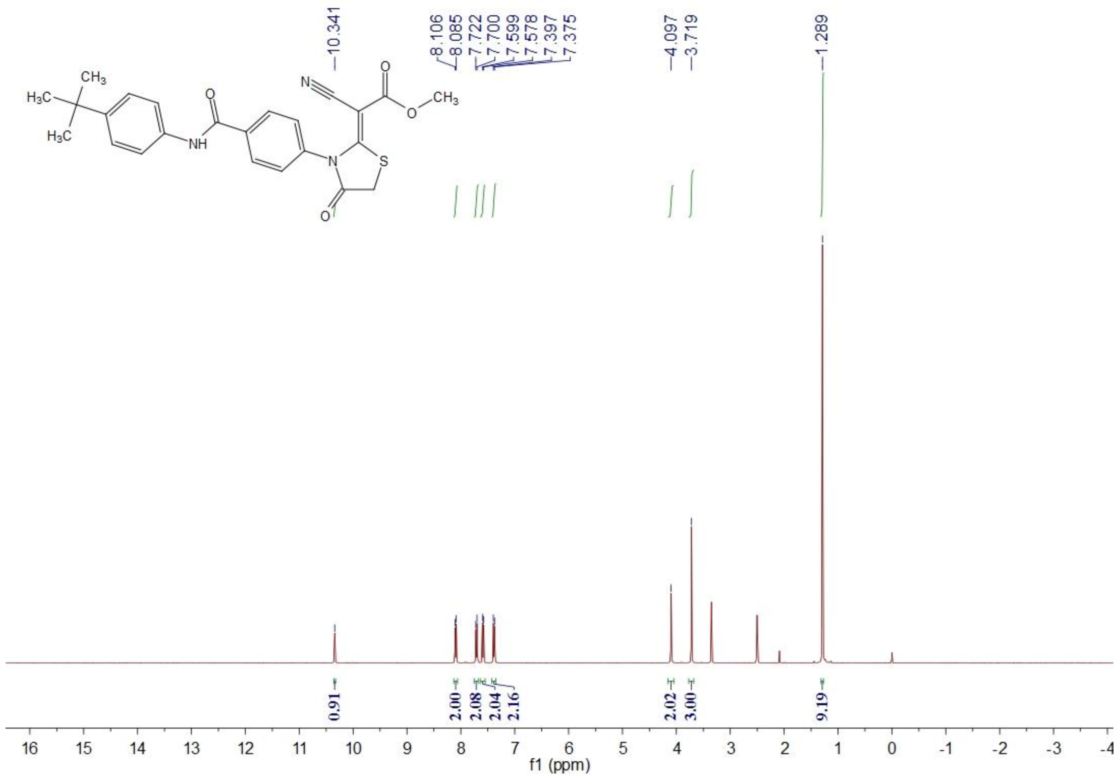

FigureS71.^{1}H-NMR of compound **37.**

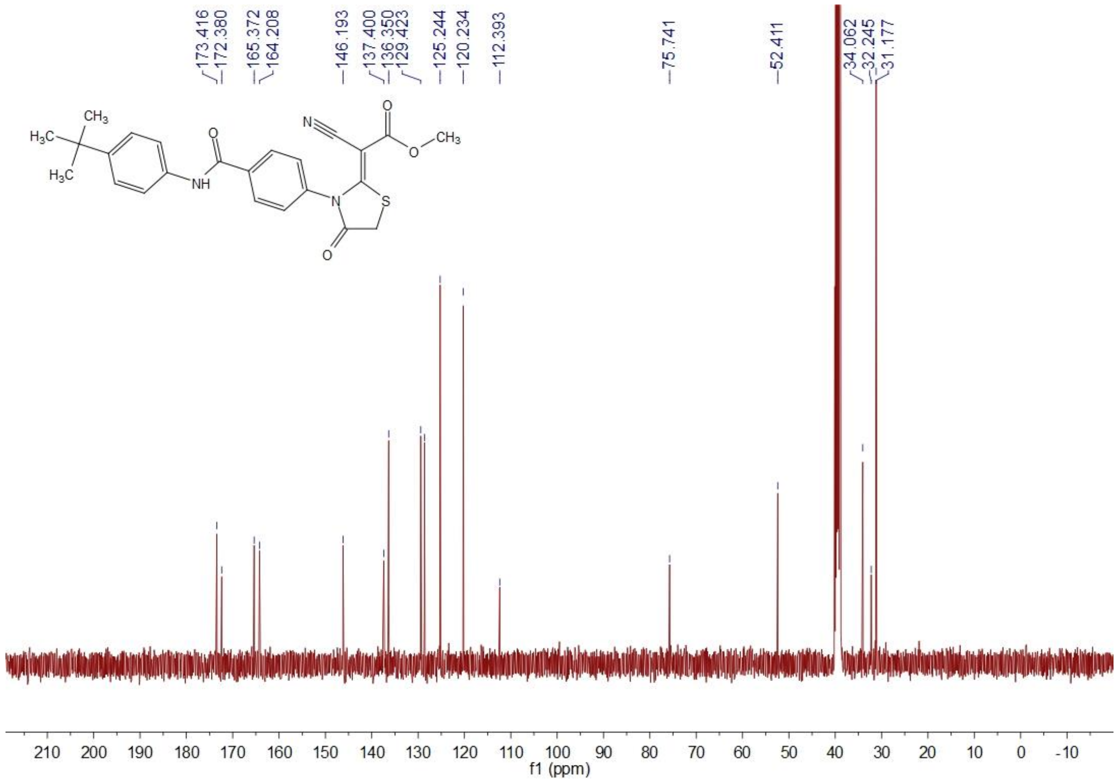

FigureS72.^{13}C-NMR of compound **37.**

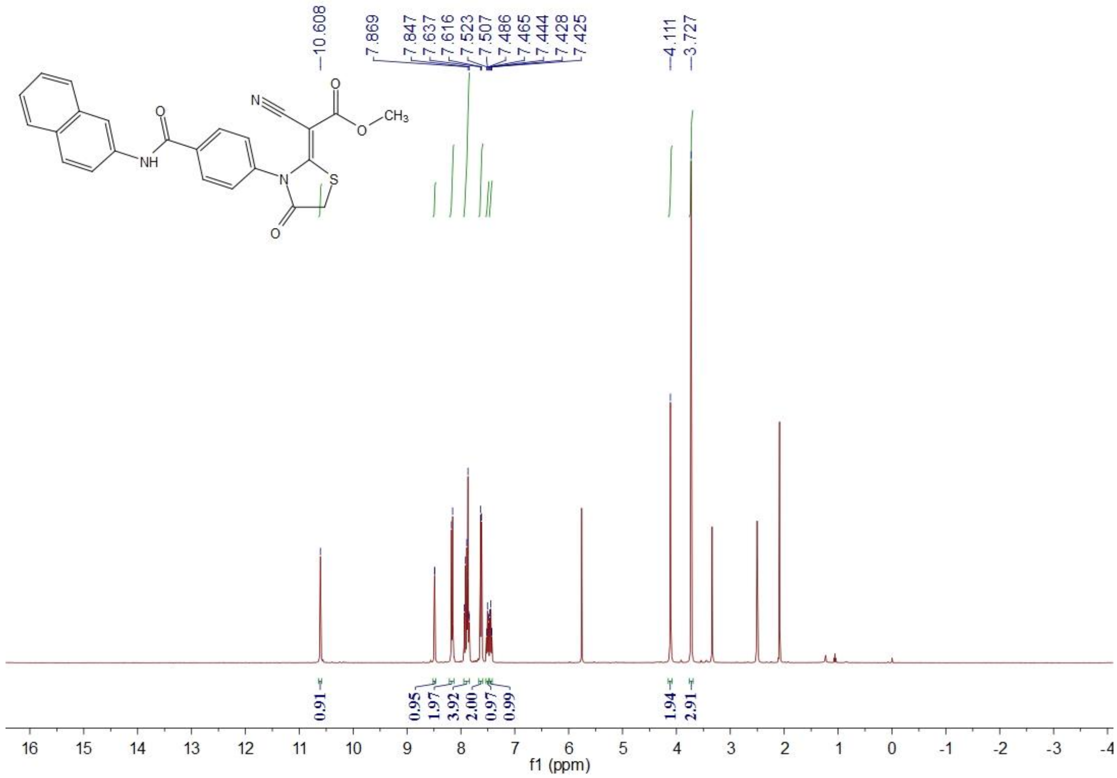

FigureS73.^{1}H-NMR of compound **38.**

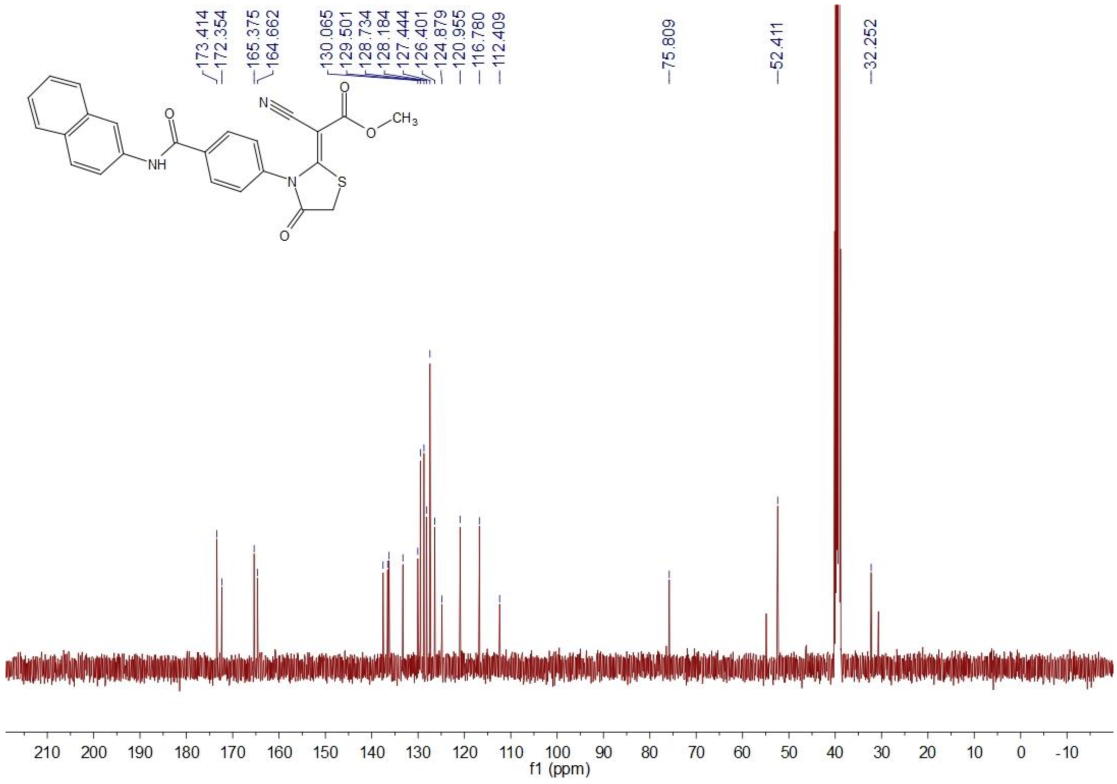

FigureS74.^{13}C-NMR of compound **38.**

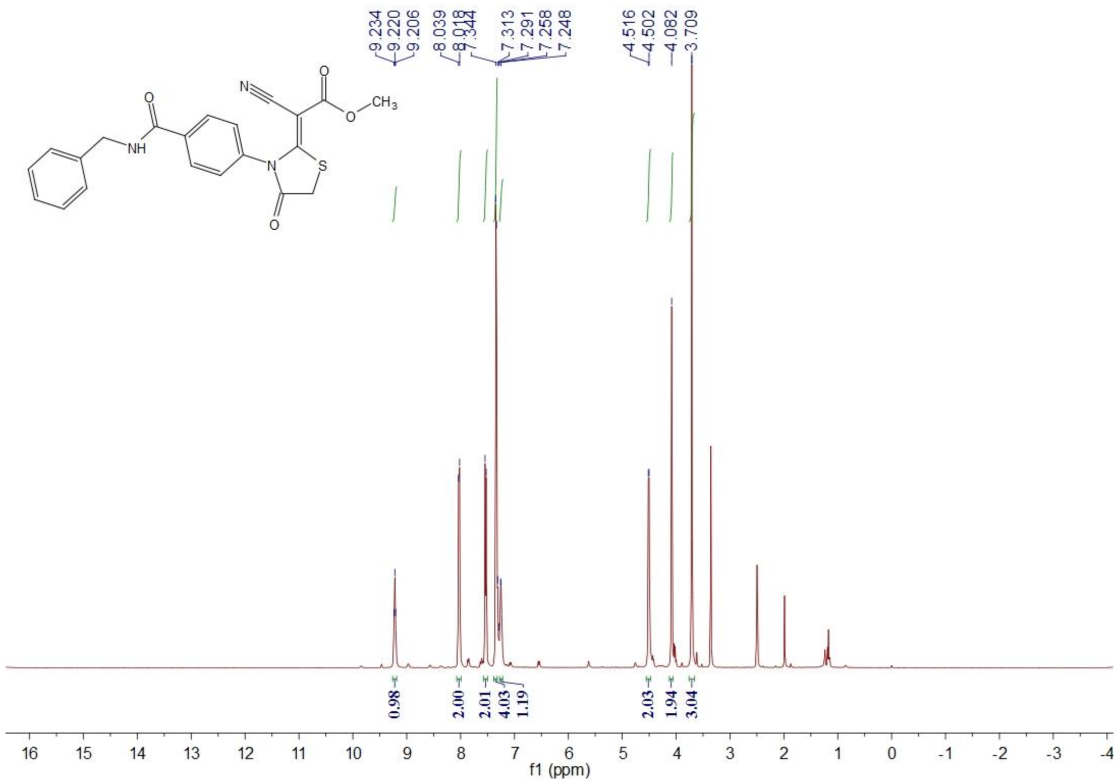

FigureS75. ^{1}H-NMR of compound **39.**

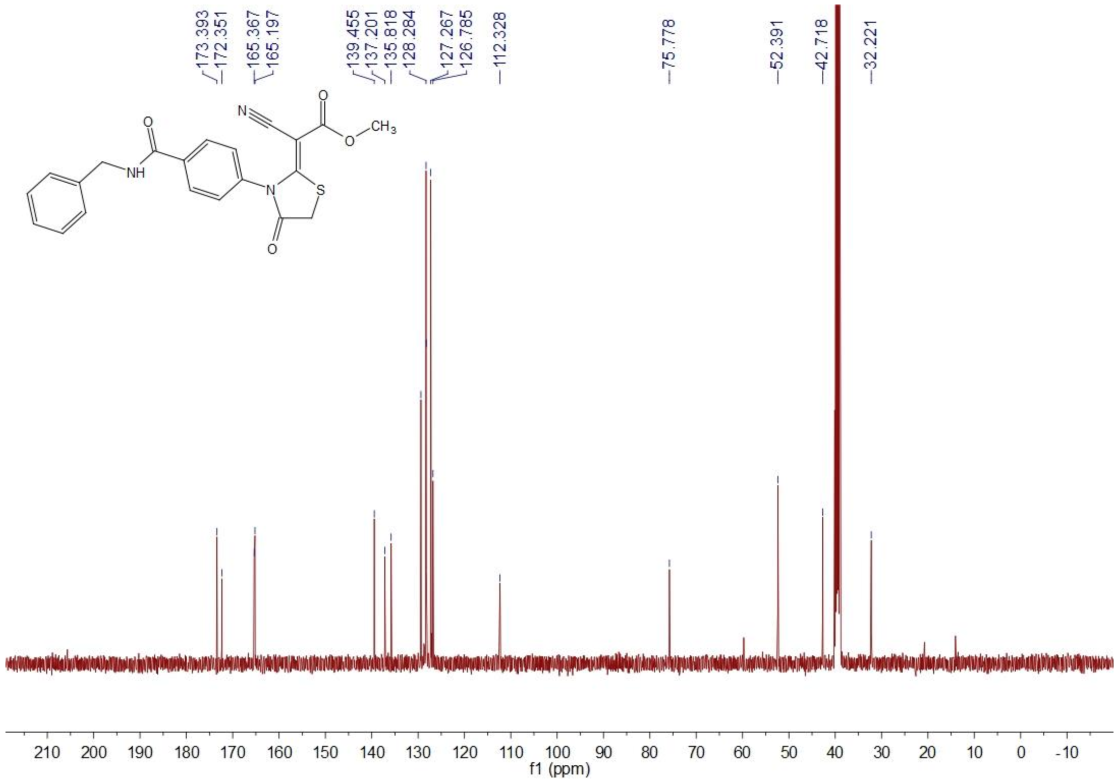

FigureS76. ^{13}C-NMR of compound **39.**

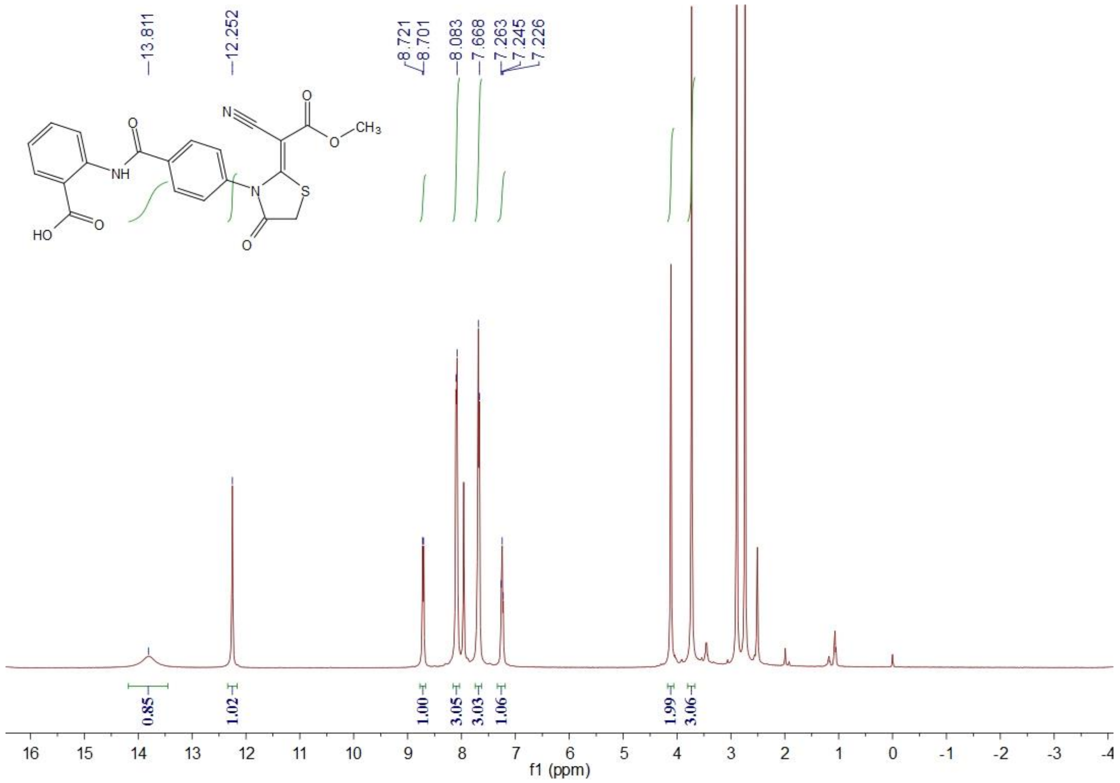

FigureS77.^{1}H-NMR of compound **40.**

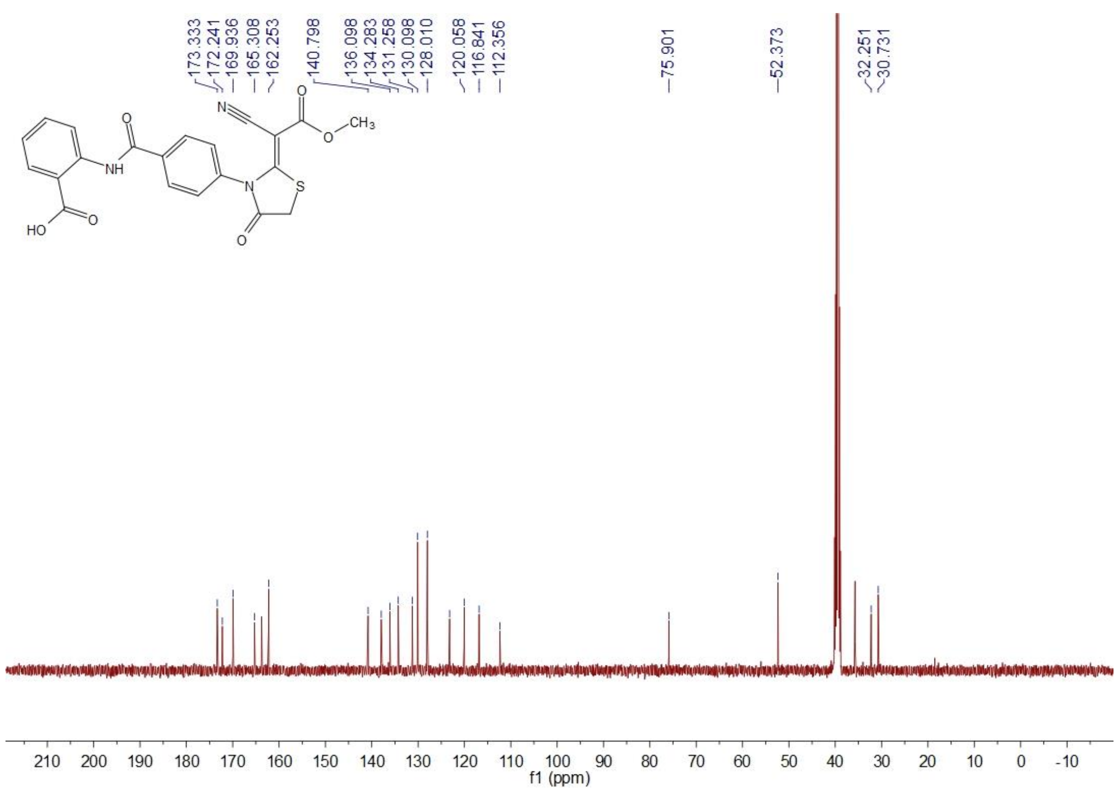

FigureS78.^{13}C-NMR of compound **40.**

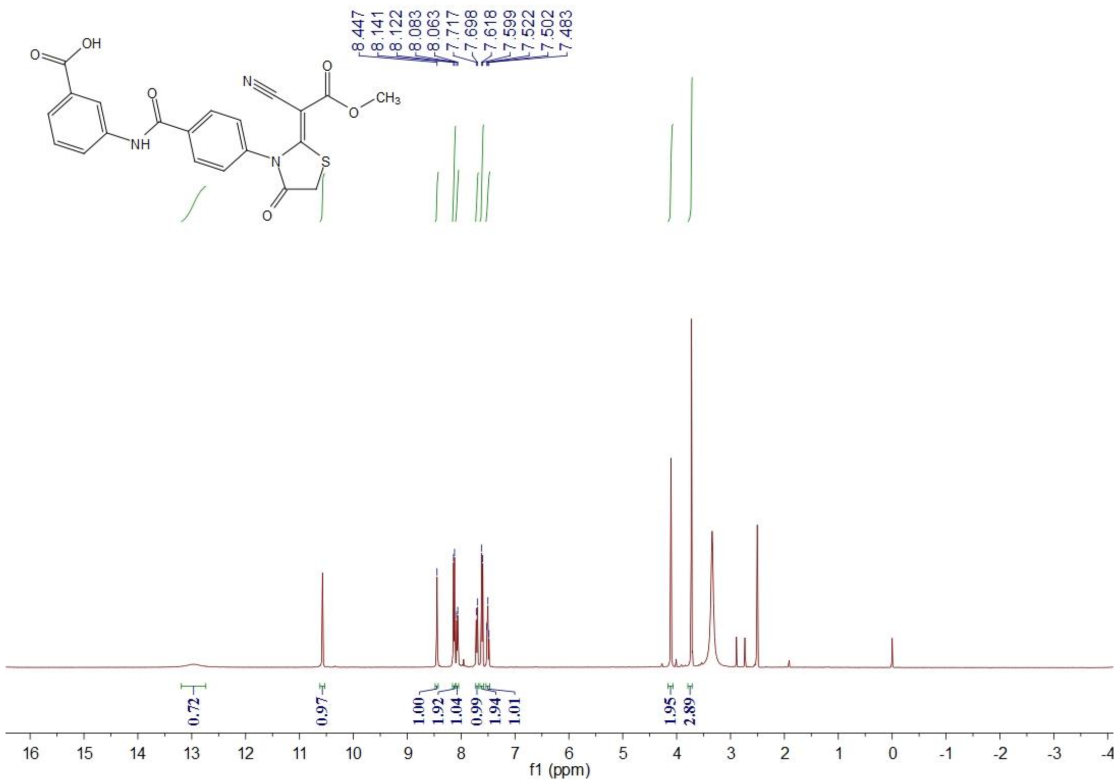

FigureS79.^{1}H-NMR of compound **41.**

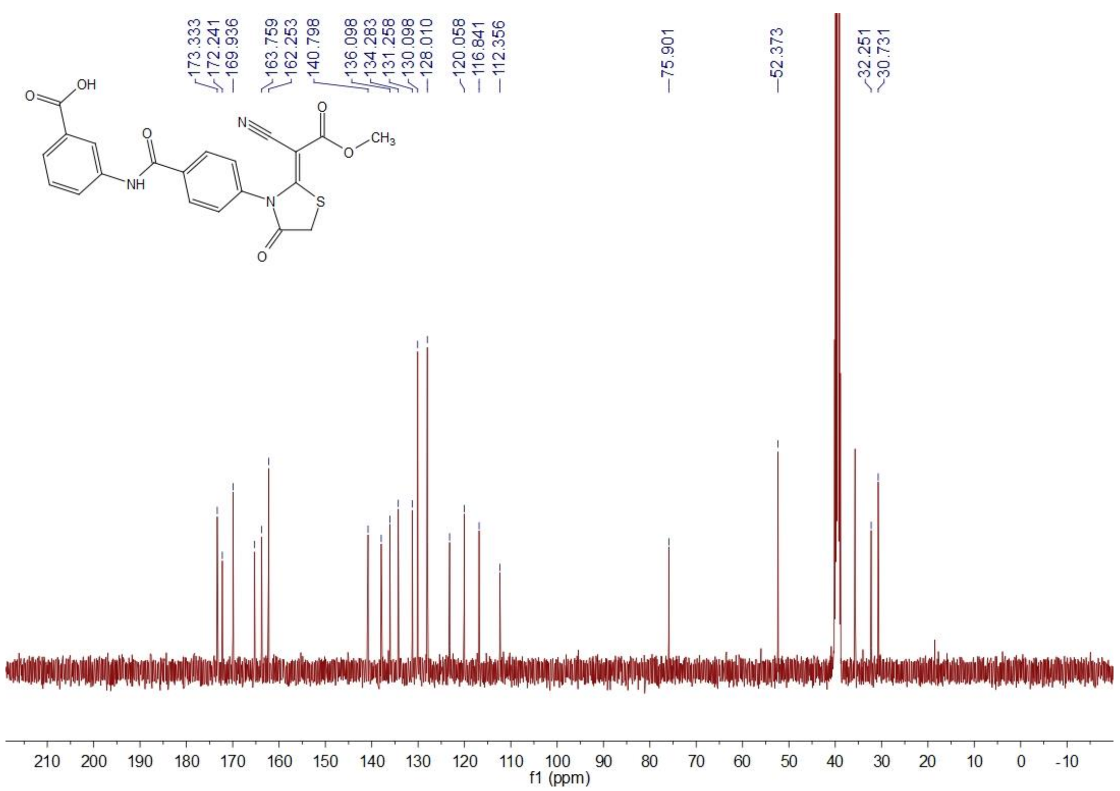

FigureS80.^{13}C-NMR of compound **41.**

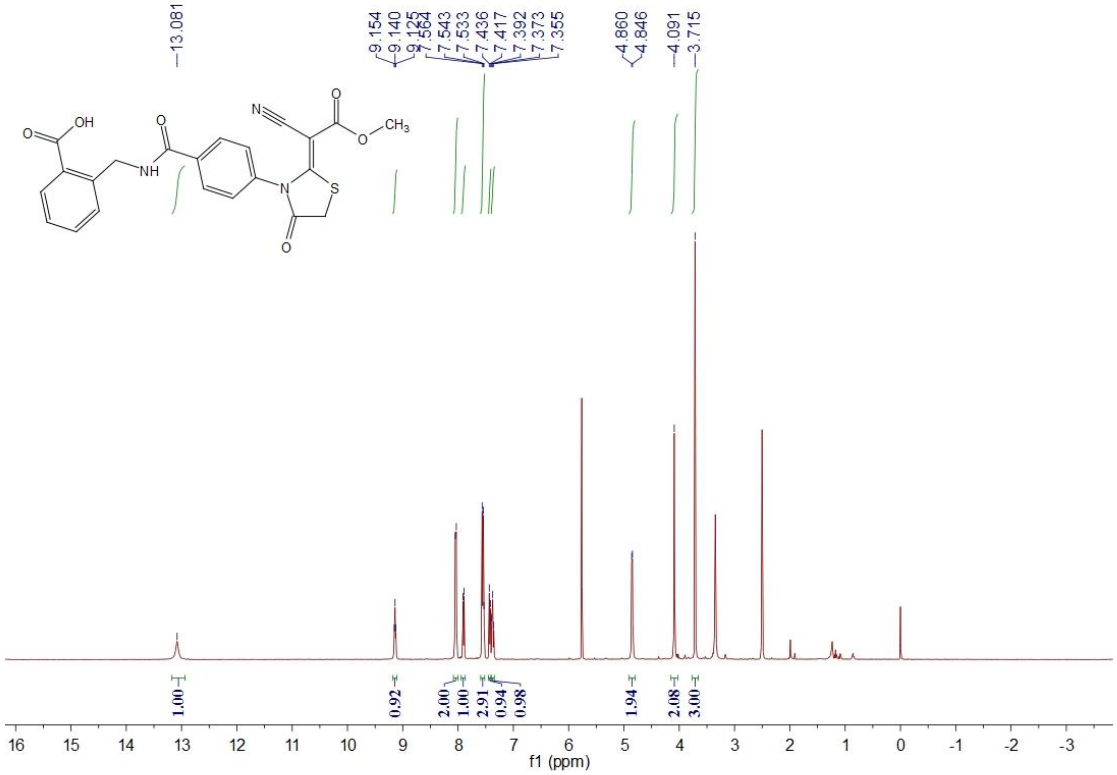

FigureS81.^{1}H-NMR of compound **42.**

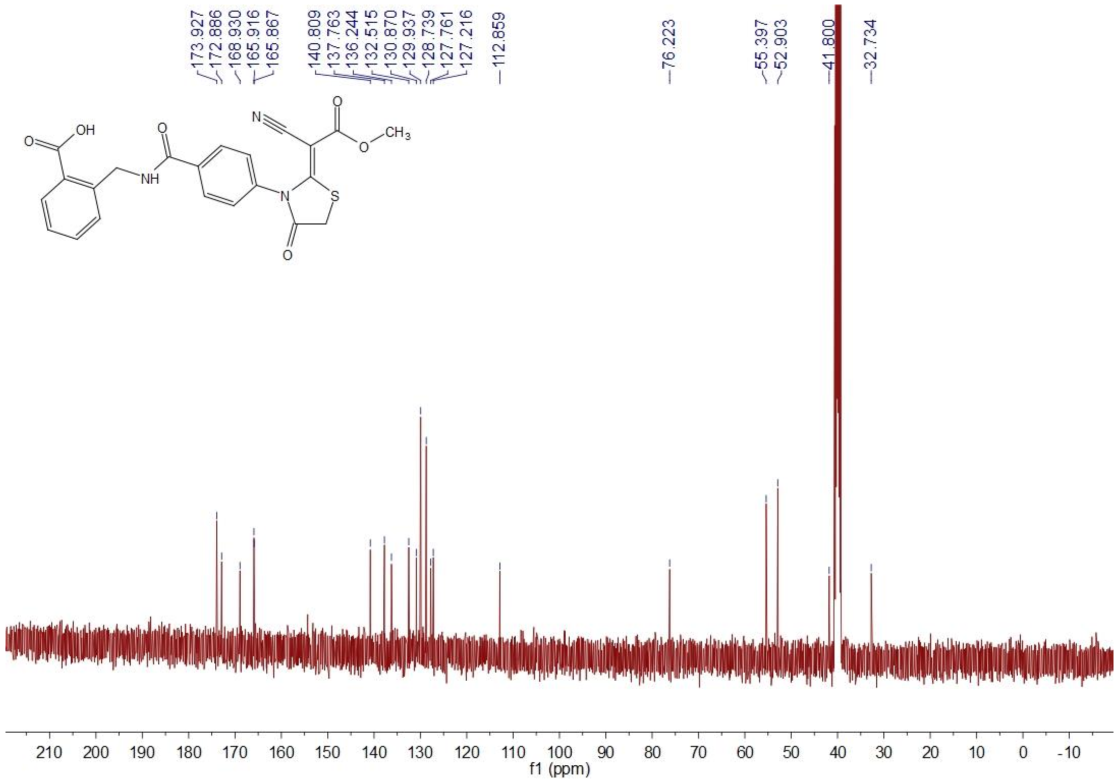

FigureS82.^{13}C-NMR of compound **42.**

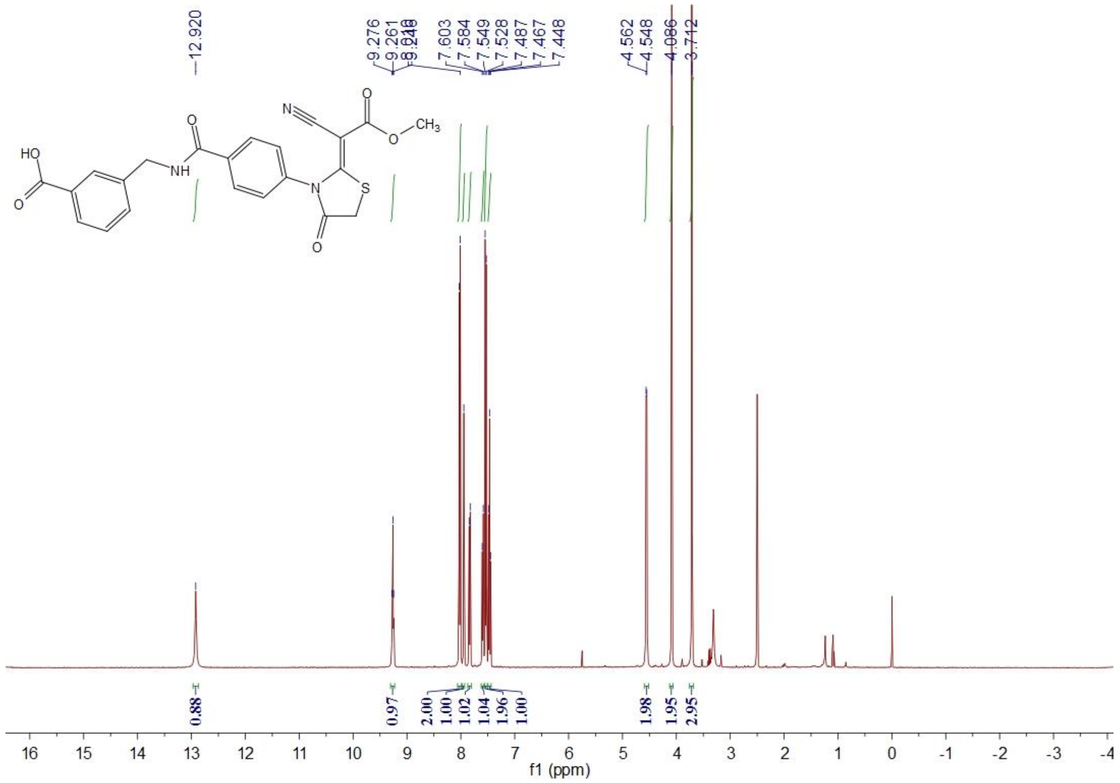

FigureS83.^{1}H-NMR of compound **43.**

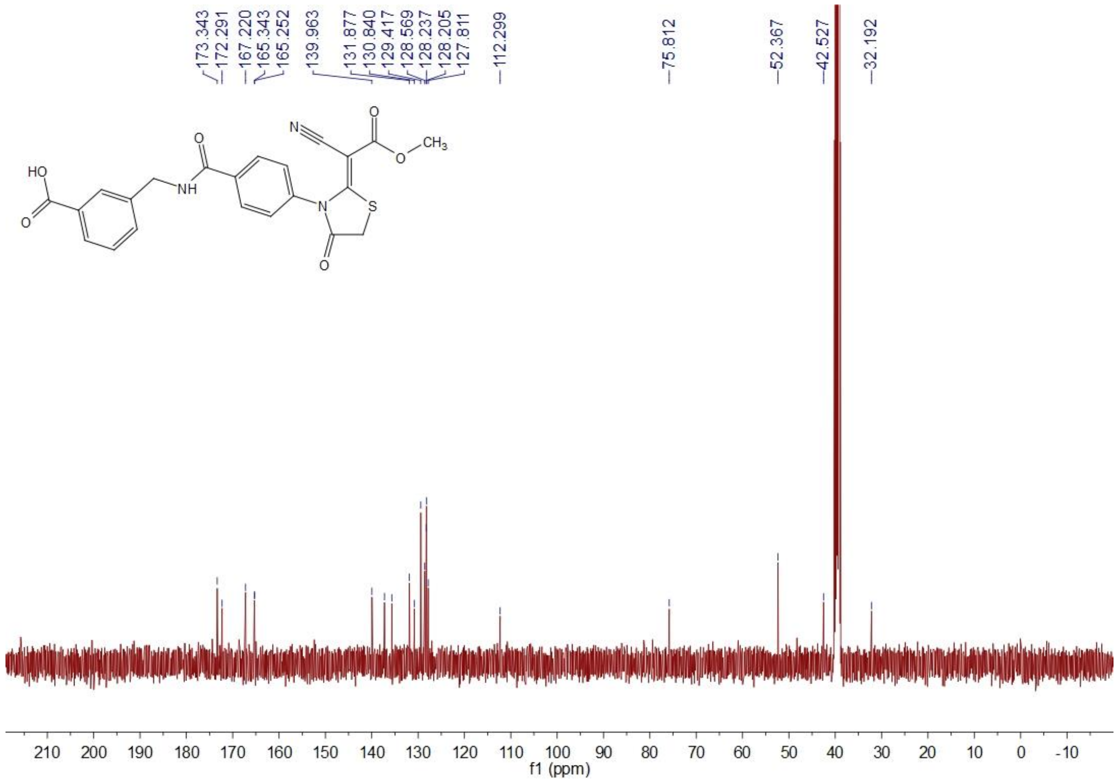

FigureS84.^{13}C-NMR of compound **43.**

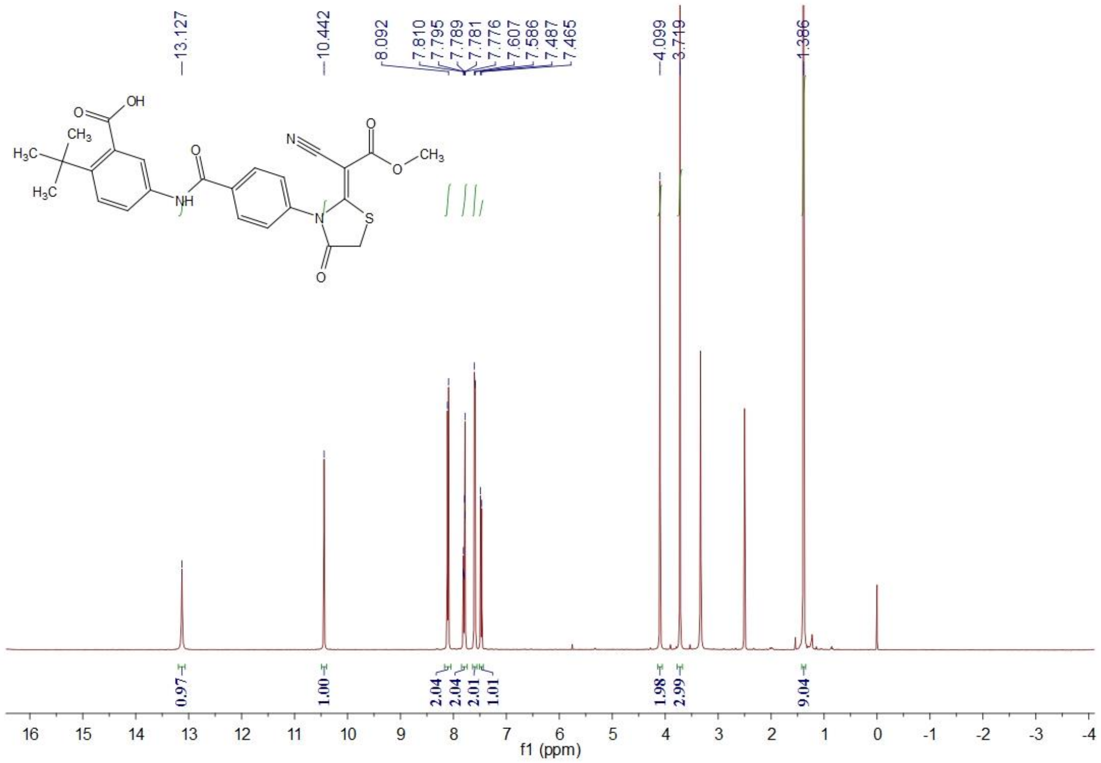

FigureS85.^{1}H-NMR of compound **44.**

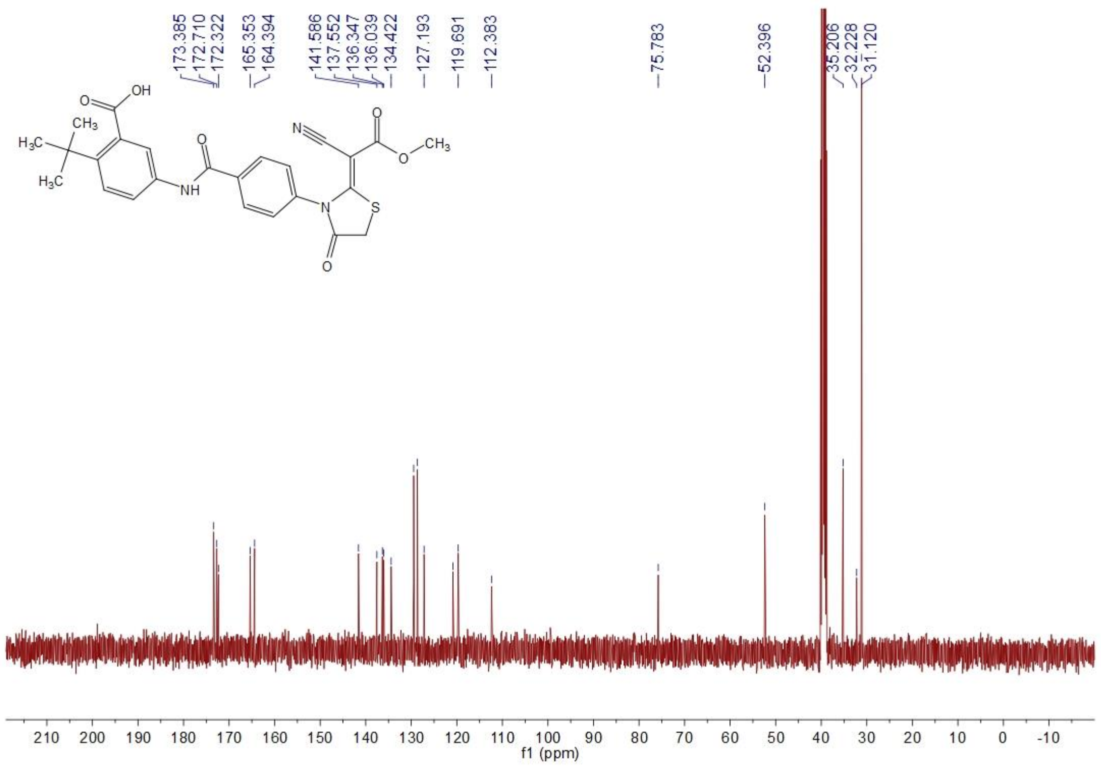

FigureS86. ^{13}C-NMR of compound **44**.

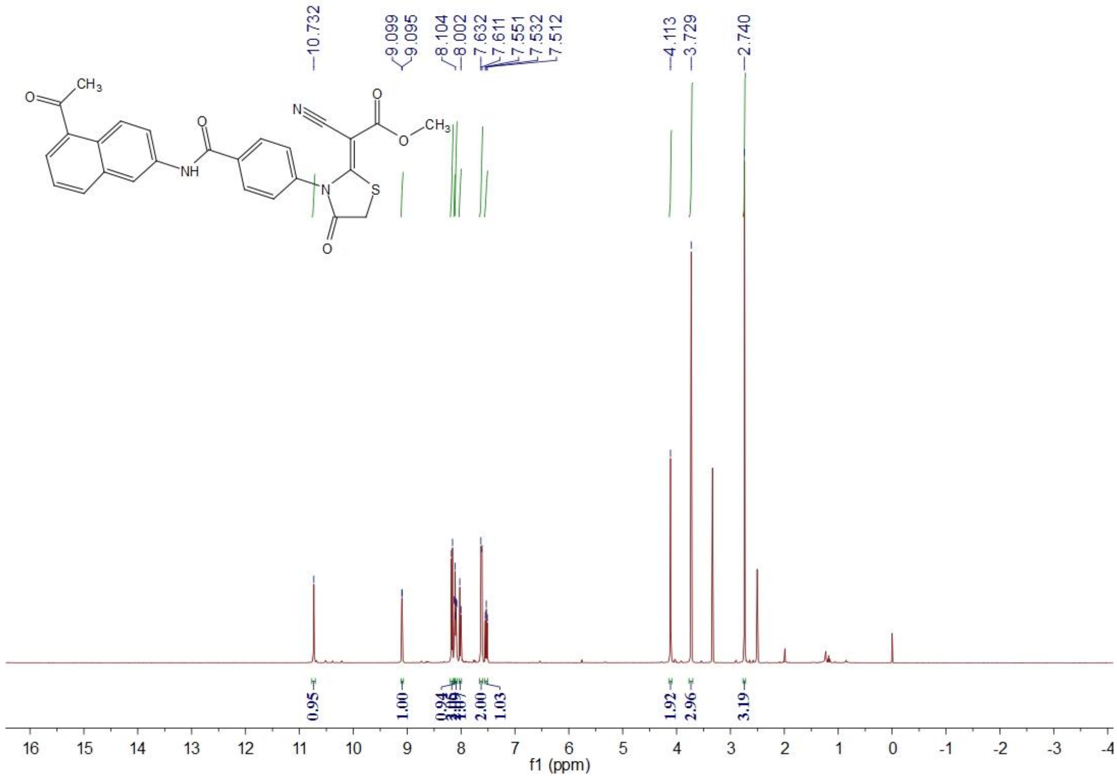

FigureS87.^{1}H-NMR of compound **45.**

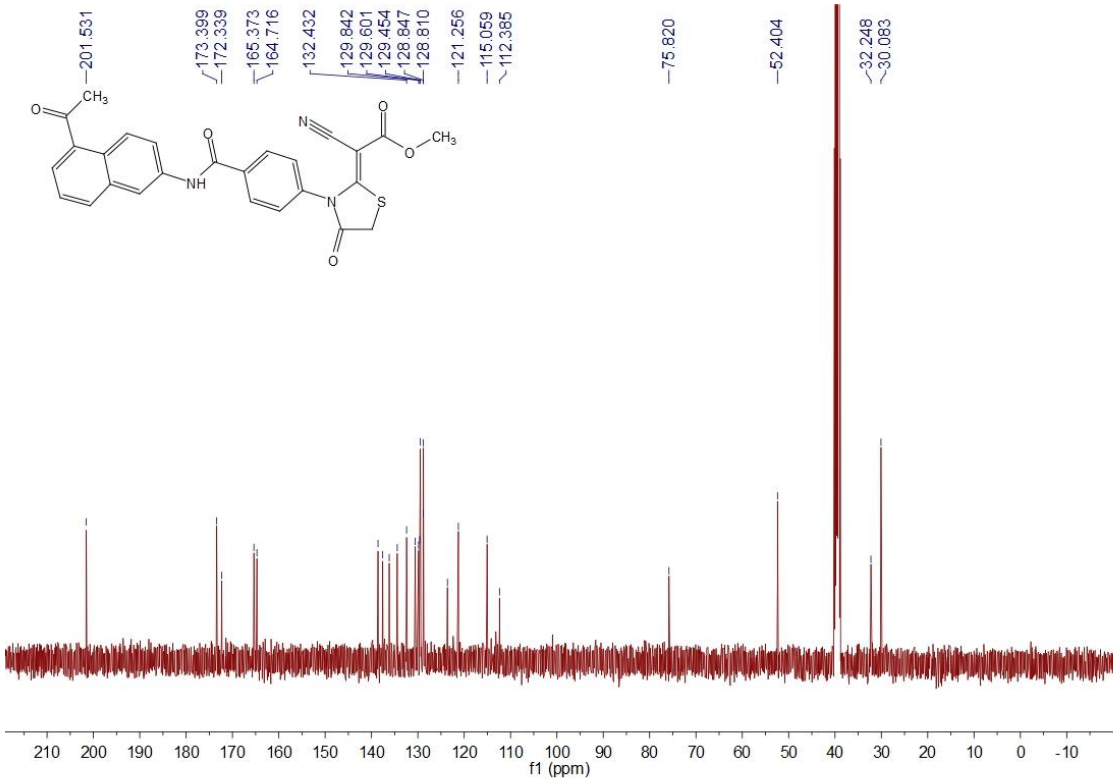

FigureS88. ^{13}C-NMR of compound **45**.

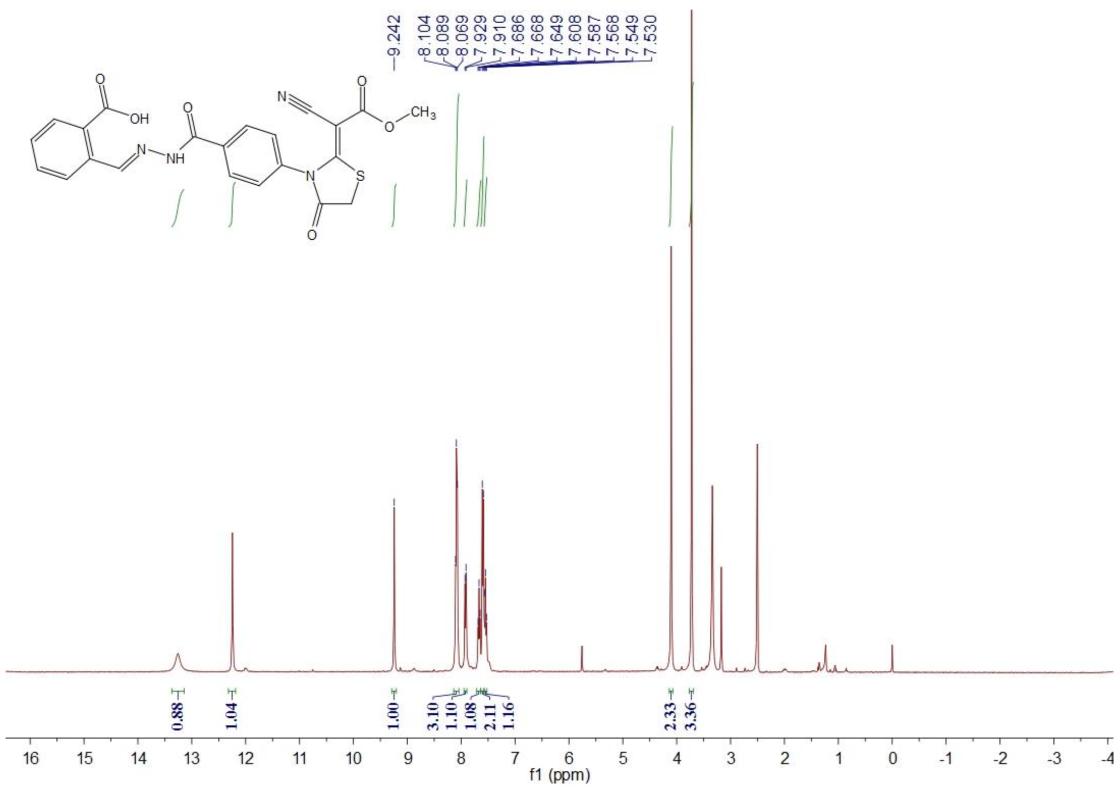

FigureS89.^{1}H-NMR of compound **46.**

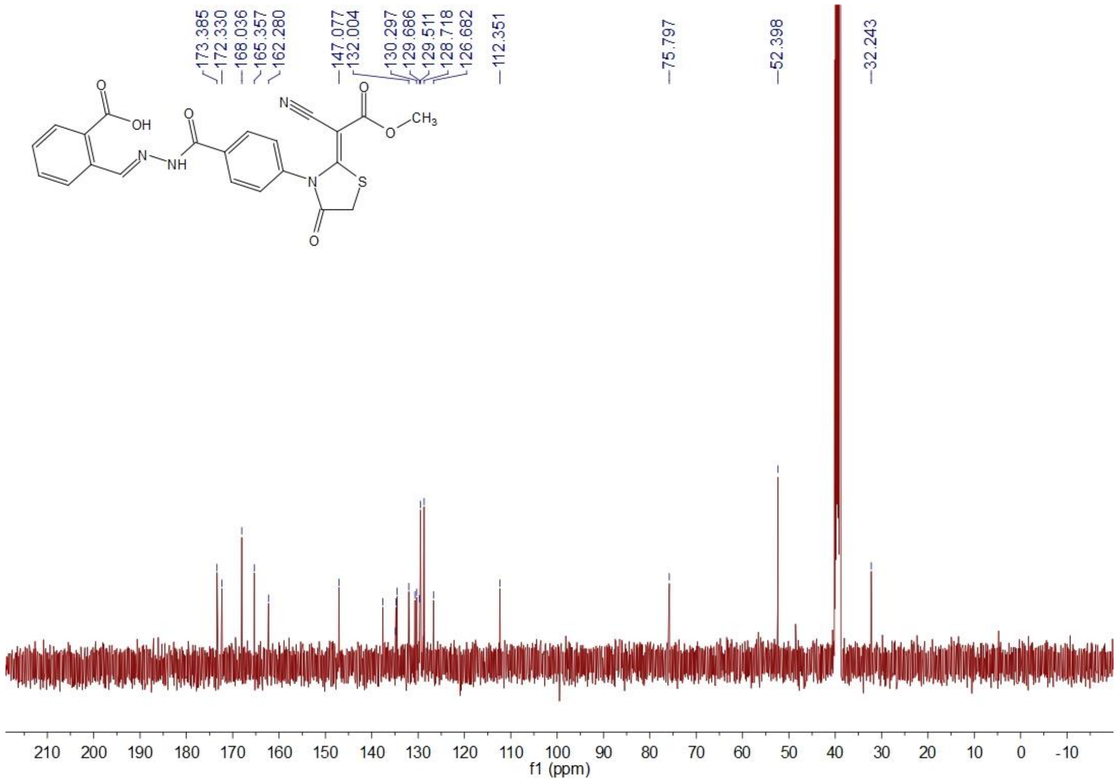

FigureS90. ^{13}C-NMR of compound **46**.
